# 6-Amino-4-aryl-7-phenyl-3-(phenylimino)-4,7-dihydro-3H-[1,2]dithiolo[3,4-b]pyridine-5-carboxamides: Synthesis, Biological Activity, Quantum Chemical Studies and In Silico Docking Studies

**DOI:** 10.3390/ijms25020769

**Published:** 2024-01-07

**Authors:** Victor V. Dotsenko, Alexander V. Bespalov, Anna E. Sinotsko, Azamat Z. Temerdashev, Vladimir K. Vasilin, Ekaterina A. Varzieva, Vladimir D. Strelkov, Nicolai A. Aksenov, Inna V. Aksenova

**Affiliations:** 1 Department of Organic Chemistry and Technologies, Kuban State University, 149 Stavropolskaya St., 350040 Krasnodar, Russia; bespalov-alex@mail.ru (A.V.B.); warzieva_rina@mail.ru (E.A.V.);; 2 Department of Chemistry, North Caucasus Federal University, 1a Pushkin St., 355017 Stavropol, Russia; radioanimation@rambler.ru (N.A.A.); inna-aksenova00@rambler.ru (I.V.A.); 3 Department of Analytical Chemistry, Kuban State University, 149 Stavropolskaya St., 350040 Krasnodar, Russia; temerdashevaz@gmail.com; 4 Department of Bioorganic Chemistry, Kuban State Technological University, 2 Moskovskaya St., 350072 Krasnodar, Russia

**Keywords:** heterocyclization, Michael addition, dithiomalondiamides, dithiolo[3,4-b]pyridines, DFT calculations, reaction mechanism studies, herbicide safeners, molecular docking, ADMET properties

## Abstract

New [1,2]dithiolo[3,4-b]pyridine-5-carboxamides were synthesized through the reaction of dithiomalondianilide (N,N′-diphenyldithiomalondiamide) with 3-aryl-2-cyanoacrylamides or via a three-component reaction involving aromatic aldehydes, cyanoacetamide and dithiomalondianilide in the presence of morpholine. The structure of 6-amino-4-(2,4-dichloro- phenyl)-7-phenyl-3-(phenylimino)-4,7-dihydro-3H-[1,2]dithiolo[3,4-b]pyridine-5-carboxamide was confirmed using X-ray crystallography. To understand the reaction mechanism in detail, density functional theory (DFT) calculations were performed with a Grimme B97-3c composite computational scheme. The results revealed that the rate-limiting step is a cyclization process leading to the closure of the 1,4-dihydropyridine ring, with an activation barrier of 28.8 kcal/mol. Some of the dithiolo[3,4-b]pyridines exhibited moderate herbicide safening effects against 2,4-D. Additionally, ADMET (Absorption, Distribution, Metabolism, Excretion, Toxicity) parameters were calculated and molecular docking studies were performed to identify potential protein targets.

## 1. Introduction

**Scheme 1:**
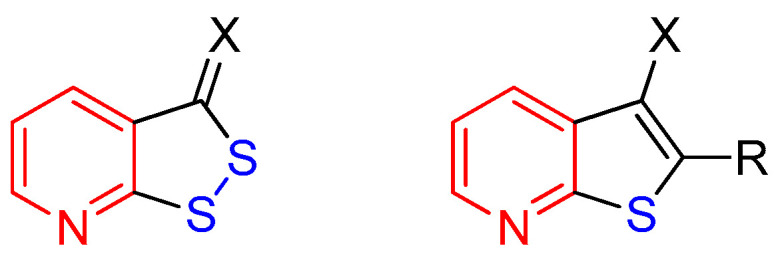
The structures of [1,2]dithiolo[3,4-b]pyridines and thieno[2,3-b]pyridines.

Because of their unique properties, sulfur-containing heterocyclic compounds attract the interest of researchers. Thus, a number of derivatives of thiophene, 1,2-dithiol, thiazole, thiadiazole, thiopyran, thiadiazine, etc., have applications in medical chemistry and optoelectronics or as agrochemicals (for reviews see [1,2,3,4,5,6,7,8,9,10,11,12,13,14,15]). The condensed heterocyclic system of [1,2]dithiolo[3,4-b]pyridine (Scheme 1) is of interest as a structural analog of other sulfur-containing heterocycles, such as thieno[2,3-b]pyridines. However, in contrast to the well-studied thienopyridines (for reviews see [16,17,18,19,20,21]), only a few papers have dealt with the synthesis and properties of [1,2]dithiolo[3,4-b]pyridines [22,23,24,25,26,27,28,29].

**Scheme 2:**
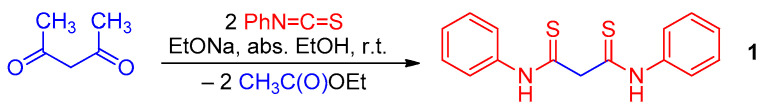
Preparation of dithiomalondianilide **1**.

Recently, we have described the synthesis of functionalized [1,2]dithiolo[3,4-b]pyri- dines based on dithiomalondianilide **1** [30,31,32]. The starting thioamide **1** is readily available from the reaction of acetylacetone with phenyl isothiocyanate in the presence of sodium ethoxide (Scheme 2) [33,34].

Dithiomalondianilide **1** is actively used as an S,S- or S,N-bidentate complexing agent [35,36,37,38,39,40,41,42,43], a lubricant [44,45] and a corrosion inhibitor [46]. However, the synthetic potential of compound **1** as a reagent for heterocyclic chemistry has not yet been fully disclosed, and heterocyclization reactions were reported in only a few papers [47,48,49,50,51,52,53,54,55,56,57,58]. As cyclic disulfides, 1,2-dithiol derivatives are of special interest in biological studies since such disulfides are particularly prone to redox reactions (for the chemistry and biological activity of disulfides, see review papers [59,60,61,62,63]). Our interest in the chemistry of dithiomalondianilide and [1,2]dithiolo[3,4-b]pyridines has subsequently led us to examine the reaction of dithiomalondianilide **1** with 2-cyanoacrylamides and to study the reaction mechanism in detail.

## 2. Results and Discussion

### 2.1. Synthesis

**Scheme 3:**
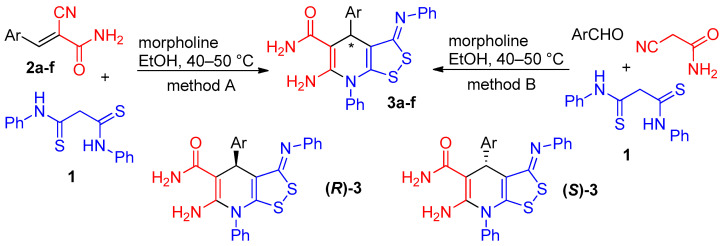
Synthesis of compounds **3**.

We found that 3-aryl-2-cyanoacrylamides **2a-f** react with dithiomalondianilide **1** in hot ethanol in the presence of morpholine to form the previously undescribed 6-amino-4-aryl-7-phenyl-3-(phenylimino)-4,7-dihydro-3H-[1,2]dithiolo[3,4-b]pyridine-5-carboxamides **3a-f**, with yields of 37–54% (Scheme 3, Method A). Dithiolopyridines **3** can also be prepared in a one-pot manner via a reaction of aromatic aldehydes, cyanoacetamide and dithiomalondianilide **1** under similar conditions (Scheme 3, Method B); however, in this case, the yields of products **3** are somewhat lower (22–43%, Table 1). The reaction proceeded smoothly in EtOH; other solvents, such as *i*-PrOH or acetone, produced rather unsatisfactory results. The nature of amines (morpholine, piperidine and triethylamine) does not significantly affect the yields of products. The prepared [1,2]dithiolo[3,4-b]pyridines **3a-f** have a chiral center C-4 and are racemic mixtures of (*R*)- and (*S*)-enantiomers.

**Figure 1:**
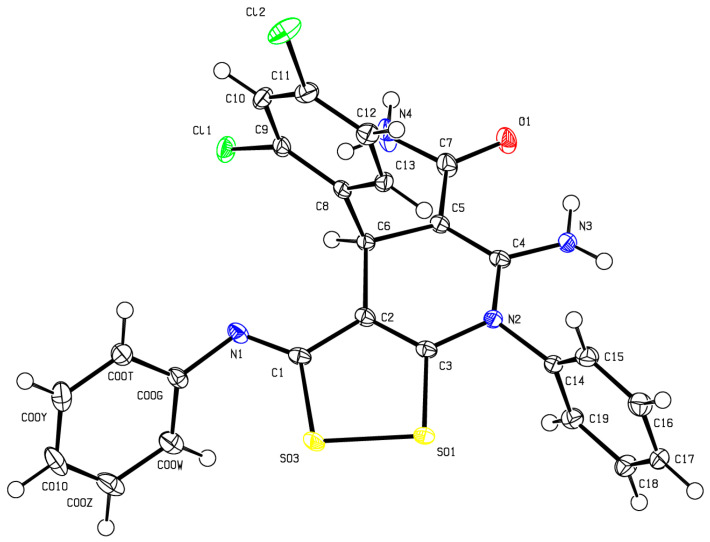
ORTEP drawing of X-ray structure for 6-amino-4-(2,4-dichlorophenyl)-7-phenyl-3-(phenylimino)-4,7-dihydro-3H-[1,2]dithiolo[3,4-b]pyridine-5-carboxamide **3d** (CCDC deposition number **2310349**).

The structures of compounds **3** were confirmed via Fourier-transform infrared spectroscopy (FT-IR), High Resolution Mass Spectrometry (HRMS) and Nuclear magnetic resonance (NMR) spectroscopy, including ^1^H NMR, ^13^C DEPTQ (Distorsionless Enhancement by Polarization Transfer), ^1^H–^1^H COSY (Correlated Spectroscopy), ^1^H–^13^C HSQC (Heteronuclear Single Quantum Coherence) and ^1^H–^13^C HMBC (Heteronuclear Multiple Bond Correlation) experiments (See Electronic Supplementary Material File). Moreover, the structure of 6-amino-4-(2,4-dichlorophenyl)-7-phenyl-3-(phenylimino)- 4,7-dihydro-3H-[1,2]dithiolo[3,4-b]pyridine-5-carboxamide **3d** was confirmed via X-ray diffraction analysis (Figure 1).

Thus, the ^1^H NMR spectra of compounds **3** show characteristic signals of two non-equivalent phenyl groups, singlets of H-4 protons at δ 4.94–5.64 ppm, broadened peaks of NH_2_ (δ 6.26–6.63 ppm) and CONH_2_ (δ 7.30–7.58 ppm) protons. In the FTIR spectra of [1,2]dithiolo[3,4-b]pyridines **3**, no C≡N absorption bands were observed, and the bands corresponding to amide C=O and C=N vibrations were detected. ^13^C NMR spectra revealed no C=S signals, but C=N carbon signals appeared at δ = 162.1–163.3 ppm.

The compounds **3** are colored in shades of yellow (from canary yellow to mustard yellow), sparingly soluble in boiling acetone or ethyl acetate, soluble in DMF or DMSO and insoluble in alcohols.

### 2.2. The Studies of the Reaction Mechanism

**Scheme 4:**
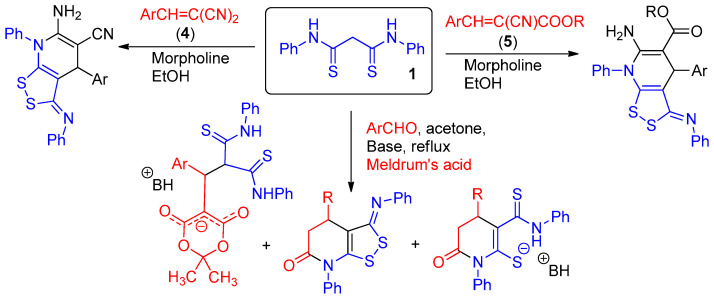
Preparation of [1,2]dithiolo[3,4-b]pyridines via Michael-type reaction of dithiomalondianilide **1** with activated alkenes as Michael acceptors.

In recent papers [30,31,32], we have described the preparation of [1,2]dithiolo[3,4-b]pyridines via the reaction of dithiomalondianilide **1** with activated Michael substrates, such as substituted acrylonitriles **4**,**5** (Scheme 4), but no detailed study of the reaction mechanism has been carried out.

Here, we would like to consider a possible mechanism for the formation of the [1,2]dithiolo[3,4-b]pyridine ring system using the reaction of compound **1** with 2-cyanoacrylamides **2** as an example. It is obvious that an oxidizing agent is required for the successful formation of the 1,2-dithiol ring. Two reagents, either oxygen in the air or an unsaturated nitrile, can play the role of an oxidizing agent. In a number of studies concerning the synthesis of pyridine derivatives starting from unsaturated nitriles (e.g., arylmethylidene malononitriles **4**), it was shown that the partially saturated pyridine intermediate easily reacts with unsaturated nitriles to yield α,β-saturated nitriles and their corresponding oxidation products, such as nicotinonitriles [64,65,66,67,68] (see Scheme S1, Electronic Supplementary Material File).

It was reported that when unsaturated nitriles were reacted in a two-fold excess, the yields of the target pyridines were increased sharply [67,68]. Although no formation of disulfides was observed in the above reactions, activated nitriles can potentially play the role of oxidizing agents for 1,2-dithiol ring closure reactions.

As a model reaction, we studied the reaction of 2-cyano-4-(methoxyphenyl)acryl- amide **2f** with dithiomalondianilide **1** in the presence of morpholine in EtOH. Compound **2f** was prepared beforehand via a Knoevenagel reaction of 4-methoxybenzaldehyde with cyanoacetamide in the presence of morpholine in EtOH at 50 °C.

**Scheme 5:**
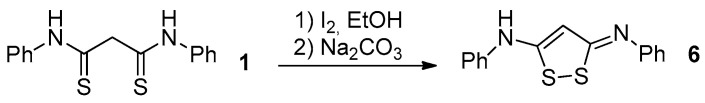
Preparation of 1,2-dithiol **6** via oxidation of dithiomalondianilide **1**.

To elucidate the nature of the oxidizing agent, the reaction between **1** and **2f** was carried out using four different variants:

(1) Under argon flow to prevent the effects of oxygen, at a ratio of **2f**:**1** = 2:1;
(2) Under an air stream at a ratio **2f**:**1** = 1:1 (Table 1, entry 6);
(3) Under atmospheric air at a ratio of **2f**:**1** = 2:1;
(4) The product of the iodine-mediated oxidation of dithiomalondianilide **1**, 1,2-dithiol **6** [47,48,69] (Scheme 5), was reacted with acrylamide **2f** under atmospheric air.

We found that in reaction (1), only trace amounts of product **3f** was detected via TLC (thin layer chromatography) and NMR after 3 h. In experiments (2) and (3), we observed the formation of a precipitate of product **3f**, though in reaction (3), TLC and HRMS showed the presence of considerable amounts of starting acrylamide **2f** in the crude product. Finally, in experiment (4), no formation of **3f** was detected, and the starting material remained unreacted. The yields of pure dithiolopyridine **3f** in experiments (2) and (3) were 54% and 55%, respectively. Thus, no significant increase in the yields was observed when a twofold excess of acrylamide **2f** was used. This fact indirectly negates the role of acrylamide **2f** as an oxidant in this reaction. Overall, from the conducted experiments (1–4), we can conclude that a) oxygen in the air is the oxidizing agent in this reaction and that b) the formation of the pyridine ring precedes the oxidation that forms the 1,2-dithiol fragment (since the product of dithiomalondianilide **1** oxidation, 1,2-dithiol **6,** did not react with acrylamide **2f** under the reported conditions).

**Scheme 6:**
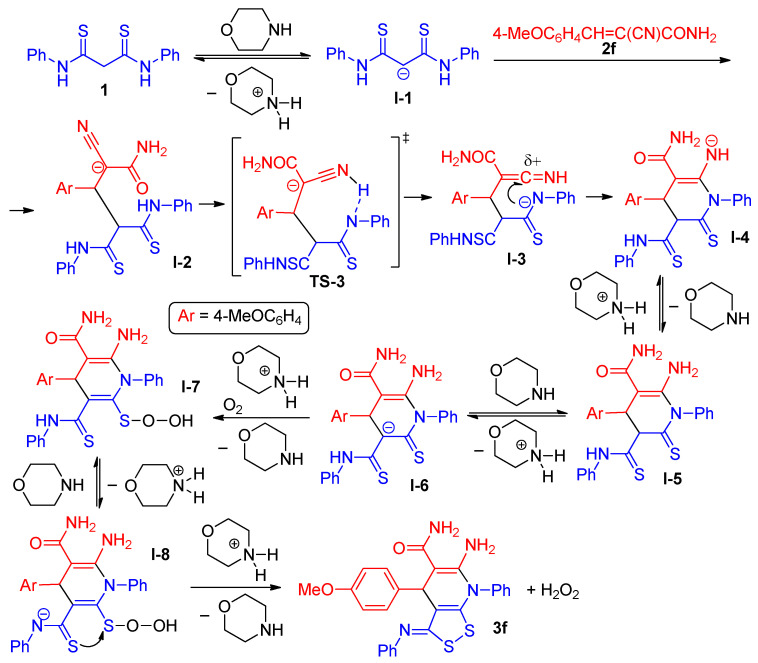
The proposed mechanism of the formation of the [1,2]dithiolo[3,4-b]pyridine ring system.

With these preliminary data in hand, we investigated the reaction mechanism in more detail. Quantum chemical study of the mechanism was performed using the ORCA 5.0.4 software package [70,71]. The search of transition states, determination of reaction trajectories, calculations of vibrational frequencies and Gibbs free energy were examined using density functional theory (DFT) with Grimme’s B97-3c composite calculation scheme [72,73], which is based on the combination of the B97 GGA functional and the def2-mTZVP basis set with the D3BJ dispersion correction [74]. Transition states were searched using relaxed scan and nudged elastic band (NEB) methods [75]. The geometry of transition states was confirmed by the presence of an imaginary vibrational frequency corresponding to the reaction coordinate. All the calculations were performed with non-specific solvation using the CPCM (Conductor-like Polarizable Continuum Model) model (with ethanol as the solvent) [76]. ChemCraft 1.8 software was used to visualize the molecular geometry and vibrational frequencies. According to the results of the quantum chemical studies, we proposed a mechanism for the formation of the [1,2]dithiolo[3,4-b]pyridine system (Scheme 6).

As we can see, the entire process of the formation of the bicyclic ring system can be divided into two main stages: heterocyclization to form dihydropyridine ring and oxidative cyclization to form the cyclic disulfide moiety. Since the reaction was carried out in the presence of excessive amounts of base (morpholine), we considered the model system of dithiomalondianilide **1** + the Michael acceptor (2-cyano-3-(4-methoxyphenyl)acryl- amide **2f**) + morpholine at the first stage. An oxygen molecule was added to the model system in the second stage. In order to determine the optimal geometry of the initial state, the most stable conformation of dithiomalondianilide **1** was determined.

**Figure 2:**
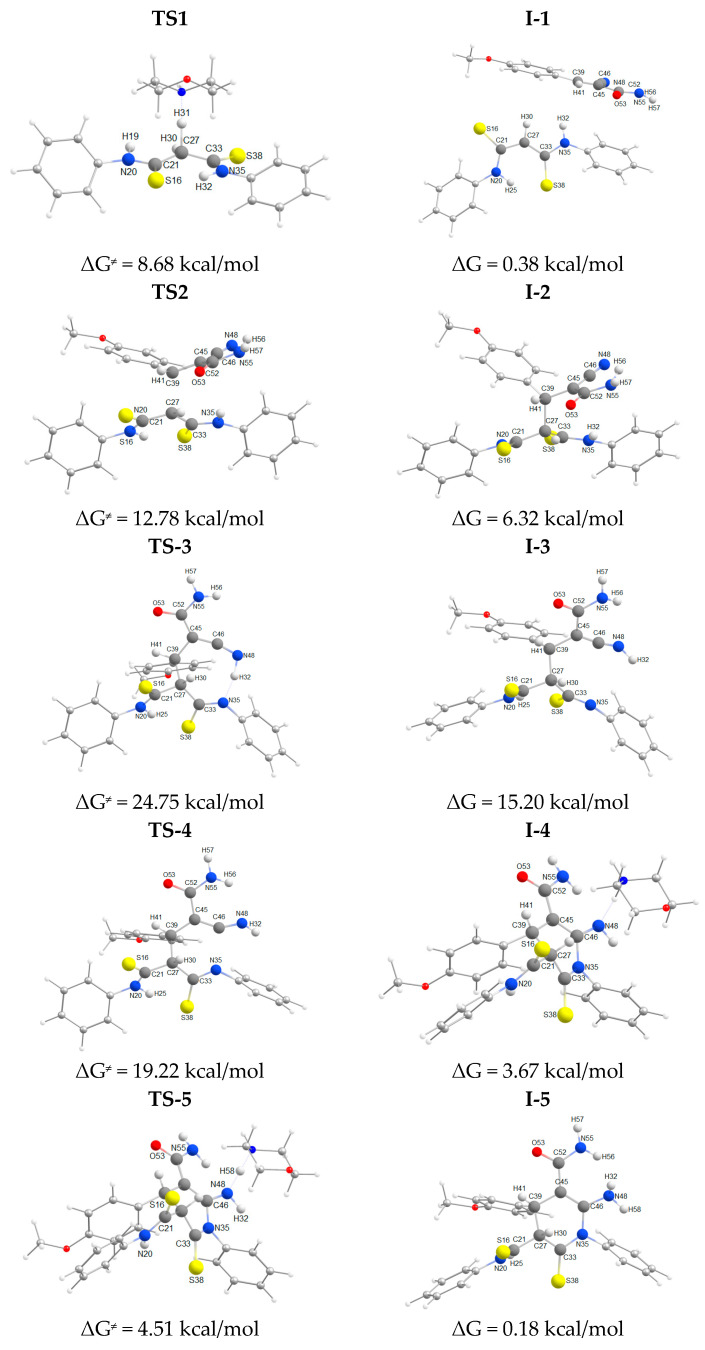
Molecular structures of intermediates **I-1**–**I-5** and transition states **TS1**–**TS5** (geometry and energy optimized at the B97-3c level). Molecules not involved in a particular elementary act are not shown in the figure for ease of perception and better visualization.

The first step of the cascade reaction mechanism involves the deprotonation of the C27 atom (Scheme 6, Figure 2) of the dithiomalondianilide **1** molecule followed by the Michael addition of the resulting anion to the C39 atom of 2-cyano-3-(4-methoxyphenyl)- acrylamide **2f**. The molecular structures of the intermediates (**I**) and transition states (**TS**) are given in Figure 2.

**Figure 3:**
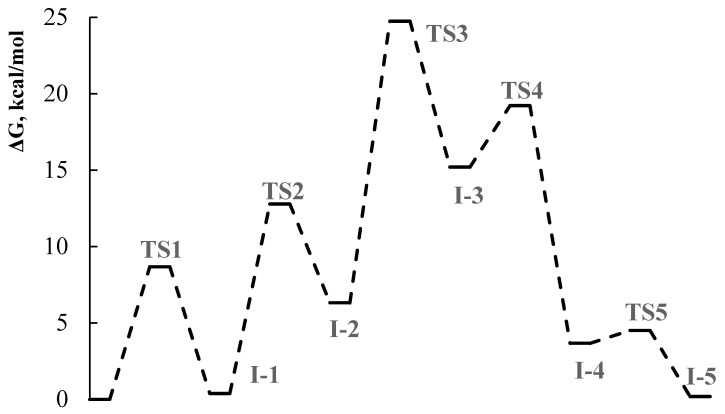
Energy profile for pyridine ring formation in EtOH (B97-3c calculations).

**Figure 4:**
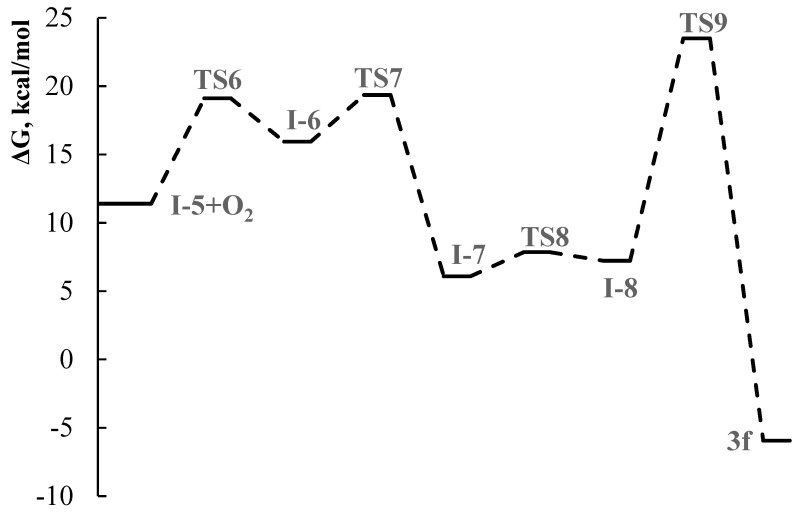
Energy profile for disulfide bond formation in EtOH (B97-3c calculations).

Next, within the resulting Michael adduct **I-2,** an intramolecular proton transfer from the N35 atom to the N48 atom occurs, yielding an eight-membered cyclic transition state **TS3**. This process has the highest activation energy and, therefore, should be considered the rate-limiting step. After the protonation of the N48 atom, an intramolecular nucleophilic attack of the N35 atom on the C46 atom occurs to close a pyridine ring. Thus, the resulting anion **I-5** then undergoes a secondary protonation of the N48 atom to form intermediate **I-6**. The energy profile of this process is shown in Figure 3, and the energy profile for oxidative cyclization to form the 1,2-dithiol fragment is given in Figure 4.

**Figure 5:**
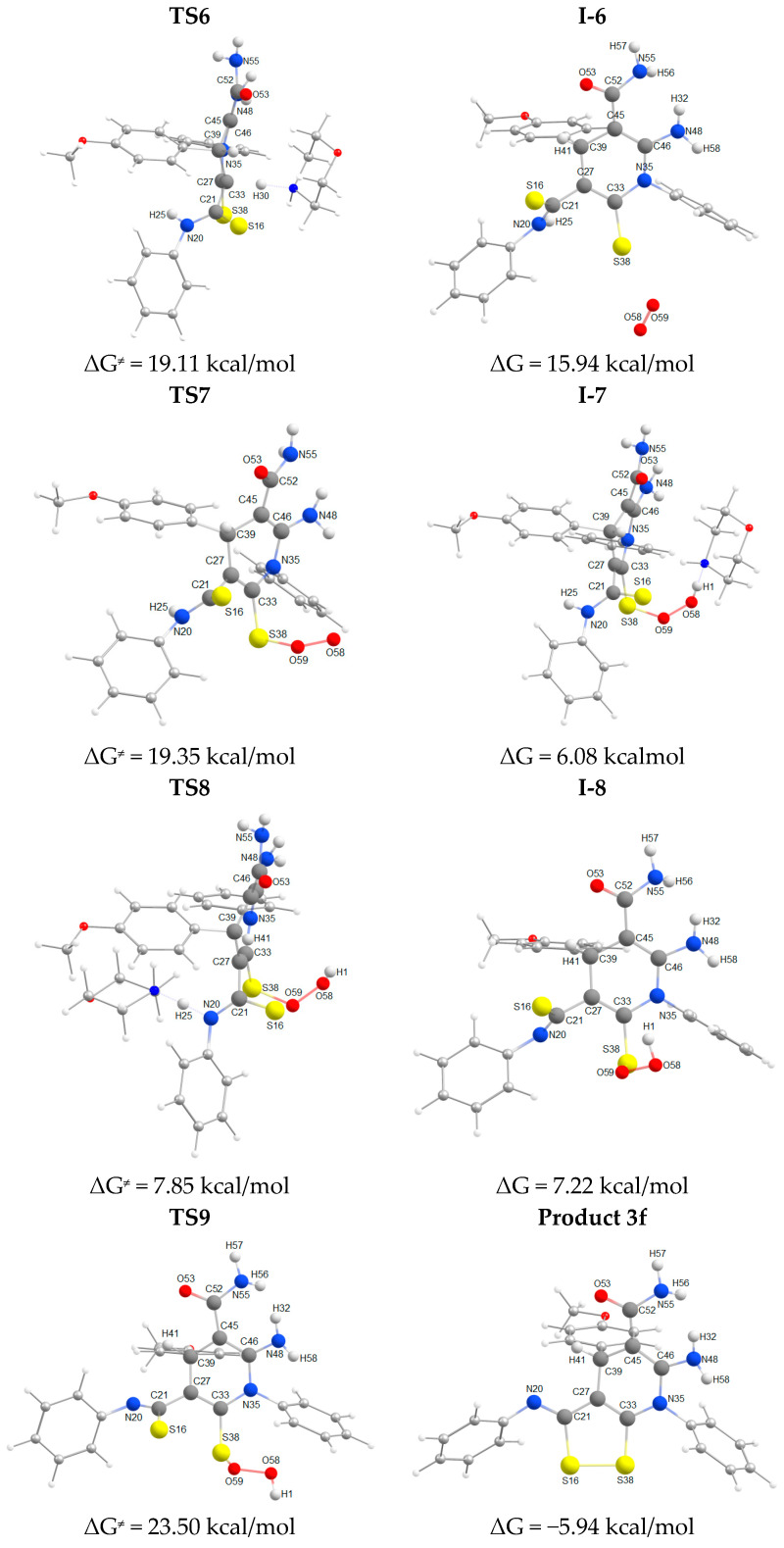
Molecular structures of intermediates **I-6**–**I-8**, dithiolopyridine **3f** and transition states **TS6**–**TS9** (geometry and energy optimized at the B97-3c level).

The oxidation of intermediate **I-5** (Scheme 6, Figure 3) involves an initial deprotonation of the C27 atom (intermediate **I-6** is formed) followed by the addition of molecular oxygen to the S38 atom to form a peroxysulfenic acid (intermediate **I-7**) (Figure 5). After the deprotonation of the CSNHPh N20 nitrogen (intermediate **I-8** is formed), an intramolecular nucleophilic substitution at the S38 sulfur atom of the hydroperoxide anion occurs to form a disulfide bond in the final product **3f**. In the process of nucleophilic substitution, the hydroperoxide anion is protonated, releasing hydrogen peroxide, which also acts as an oxidizing agent.

Overall, during the first stage of dihydropyridine ring formation, the highest activation energy (24.8 kcal/mol) corresponds to the intramolecular cyclization that occurred at the cyano group. Apparently, this is the rate-limiting step for the entire process since the highest activation barrier at the second stage (the formation of the disulfide bond/1,2-dithiol ring closure) is somewhat lower (23.5 kcal/mol).

### 2.3. ADMET Calculations and Molecular Docking Studies

Despite the relatively poor study of dithiolopyridines and their properties, it is known that some 3H-[1,2]dithiolo[3,4-b]pyridines showed antimycobacterial activity [27] and moderate antibacterial activity [22]. As close structural analogs/bioisosters of thieno[2,3-b]pyridines, [1,2]dithiolo[3,4-b]pyridines are of interest as promising candidates for bioscreening. The compounds **3a-f** were evaluated for compliance with Lipinski’s “rule of five” [77], and the ADMET parameters were estimated using these free online resources: the SwissADME website (http://www.swissadme.ch/, accessed on 30 December 2023), the GUSAR service (http://www.way2drug.com/gusar/acutoxpredict.html, accessed on 30 December 2023), the OSIRIS Property Explorer service (https://www.organic-chemistry.org/prog/peo/, accessed on 30 December 2023), and the AdmetSAR website (http://lmmd.ecust.edu.cn/admetsar2, accessed on 30 December 2023).

The analysis for compliance with Lipinski’s “rule of five” is intended to predict drug -likeness and bioavailability for an oral therapeutic agent using its physicochemical properties. The following parameters were calculated: *c*Log*P* (logarithm of the distribution coefficient between *n*-octanol and water, *log*(c_octanol_/c_water_), solubility (*log* S), topological polar surface area (TPSA), a number of toxicological characteristics, including the risks of side effects (mutagenic, oncogenic and reproductive effects) and a similarity parameter with known drugs (drug-likeness); a general assessment of the pharmacological potential of the compound (drug score) was also carried out. Since (*R*)- and (*S*)-stereoisomers of compound **3** revealed the same values in the in silico calculations, the results presented in Table S12 (Electronic Supplementary Material File) apply equally to both enantiomers.

As we can see from the Table S12, for all compounds except **3b** and **3d**, the *c*Log*P* values do not exceed 5.0, which indicates good absorption and permeability (which was expected) [77]. However, most of the compounds **3** do not fit the Lipinski’s rule of five in terms of molecular weight (MW ≥ 500 Da). Furthermore, these compounds **3** had poor calculated solubility (*log*S < −4) and did not pass the Veber filter (TPSA ≥ 140 Å^2^) [78]. The results of the ADMET calculations are given in Tables S13–S24 (Electronic Supplementary Material File). In general, good gastrointestinal absorption and blood–brain barrier permability were predicted for all the compounds **3a-f**. However, the calculations revealed probable hepatotoxicity and mitochondrial toxicity. At the same time, low acute oral toxicity was predicted. The calculation derived from the GUSAR service assigns all compounds to categories 4 (LD_50_ > 300–2000 mg/kg) or 5 (LD_50_ > 2000–5000 mg/kg) according to OECD criteria [79]. Overall, these compounds **3** do not meet certain oral availability criteria as lead molecules, e.g., poor solubility, some violations for Ghose filter (MW > 480, molar refractivity (MR) > 130), Veber filter (violation: TPSA > 140 Å^2^), Egan filter (violation: TPSA > 131.6 Å^2^), Muegge filter (violation: TPSA > 150 Å^2^) and insaturation parameter (fraction of Csp^3^ < 0.25).

For both the (*R*)- and (*S*)-enantiomers of each of the 3H-[1,2]dithiolo[3,4-b]pyridines **3a-f**, the most likely protein targets were predicted using the GalaxyWEB server (https://galaxy.seoklab.org/index.html, accessed on 30 December 2023) [80,81]. We used GalaxySagittarius-AF, the newest protein–ligand docking protocol that uses AlphaFold models for drug-like compounds. The 3D structures of (*R*)- and (*S*)-enantiomers of compounds **3a-f** were pre-optimized via molecular mechanics in the MM2 force field for geometry optimization and energy minimization. The molecular docking studies were performed in binding compatibility prediction mode using PDB + AlphaFold models and re-ranking using docking mode. Table S25 (Supplementary Materials File) shows the docking results for 25 target-ligand complexes for both isomers of **3a-f** with the minimum binding free energy ΔG_bind_ and the best protein–ligand interaction score. The predicted protein targets are marked using identifiers in the Protein Data Bank (PDB) and in the UniProt database.

**Figure 6:**
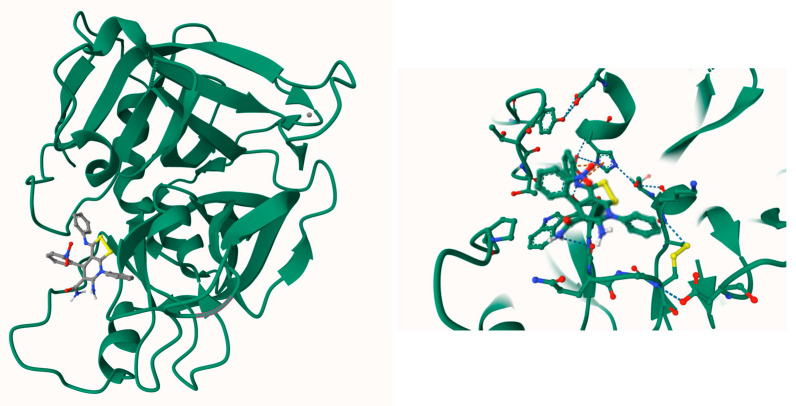
Position of compound (*R*)-**3a** in the site of coagulation factor VII (PDB ID 4yt6_H) via molecular docking.

As we can see from Table S25, for both the (*R*)- and (*S*)-enantiomers of 3H-[1,2]dithiolo[3,4-b]pyridines **3a-f**, a similar pool of protein targets is predicted, but the scoring function values are different. The most common targets for 3H-[1,2]dithiolo[3,4-b]pyridines **3a-f** are human trypsin 1 (PDB ID 1trn_A), with ΔG_bind_ values ranging from −19.65 kcal/mol to −21.415 kcal/mol; urokinase-type plasminogen activator (PDB ID 3khv), with ΔG_bind_ = −21.003…−24.09 kcal/mol; coagulation factor VII (PDB ID 4yt6_H), with ΔG_bind_ = −18.771…−21.267 kcal/mol; focal adhesion kinase 1 (FAK1, PDB ID 4gu6_A), with ΔG_bind_ = −23.786…−25.831 kcal/mol; proto-oncogene tyrosine-protein kinase LCK (PDB ID 3ac8_A), with ΔG_bind_ = −21.315…−23.956 kcal/mol; serine/threonine-protein kinase B-raf (PDB ID 4mnf), with ΔG_bind_ = −20.983…−24.744 kcal/mol; and sterile alpha motif and leucine zipper containing kinase ZAK (PDB ID 7yaw), with ΔG_bind_ = −23.539…−25.157 kcal/mol. One of the protein–ligand complexes was visualized using the GalaxySagittarius-AF software package (https://galaxy.seoklab.org/index.html, accessed on 30 December 2023) and is shown in Figure 6.

### 2.4. Agrochemical Studies

Recently, some 3-aminothieno[2,3-b]pyridines [82,83,84] and related 3-imino-3H- [1,2]dithiolo[3,4-b]pyridines [32] were recognized as effective 2,4-D herbicide safeners and plant growth regulators.

2,4-D (2,4-dichlorophenoxyacetic acid) is an herbicide that displays a relatively low toxicity in humans and is actively used for the chemical weeding of cultivated plants [85]. The chemical weeding of crops is an important element in the protection of agricultural crops from weeds because weeds reduce the yield of the most important crops by 15–25%. However, it should be noted that herbicides, as plant-killing agents, are toxic not only to weeds but also to the sunflower, and the widely used 2,4-dichlorophenoxyacetic acid is highly toxic to the latter. For example, a dose of 0.5–0.8 kg/ha of the active ingredient 2,4-D is recommended for weed control in crops of resistant cereals, and for the sunflower, a dose as small as 15–18 g/ha of the active ingredient leads to a 40–60% reduction in the crop yield.

One possible solution to this problem is to use so-called herbicide antidotes, or herbicide safeners. Herbicide safeners (for reviews, see [86,87,88]) are agrochemicals that are able to neutralize phytotoxins in plants via different mechanisms, thus protecting crop plants from herbicide injury. Herbicide safeners are harmless to crop plants but do not affect the activity of herbicides against weeds.

The compounds **3b**, **3d** and **3f**, as close structural analogs of known active antidotes [32,82,83,84], were examined as herbicide safeners on sunflower seedlings using the reported procedure [82]. The antidote effect *A* was determined as a ratio of the length of the hypocotyls (or roots) of sunflower seedlings in the “herbicide + antidote” experiments to the length in the reference group (where the seedlings were treated with 2,4-D only) (Equation (1)):

*A* = (*L*_exp_/*L*_ref_) × 100%, (1)

where *L*_exp_ is the organ length (mm) in the group of seedlings treated with the herbicide and antidote and *L*_ref_ is the organ length (mm) in the reference group of sunflower seedlings.

We found that dithiolopyridines **3b**, **3d** and **3f** exhibited a weak to moderate 2,4-D antidote effect in the laboratory experiments (Table 2).

As we can see from Table 2, 3H-[1,2]dithiolo[3,4-b]pyridines **3b**, **3d** and **3f**, as herbicide safeners, reduced the negative effects of 2,4-D on sunflower seedling hypocotyls by 16–32% and on sunflower seedling roots by 16–33%.

## 3. Materials and Methods

^1^H and ^13^C DEPTQ NMR spectra and 2D NMR experiments were recorded in solutions of DMSO-d_6_ on Bruker AVANCE-III HD (Bruker BioSpin AG, Fällanden, Switzerland) and Agilent 400/MR (Agilent Technologies, Santa Clara, CA, USA) instruments (at 400 MHz for ^1^H and 101 MHz for ^13^C nuclei). Residual solvent signals were used as internal standards in DMSO-d_6_ (2.49 ppm for ^1^H and 39.50 ppm for ^13^C nuclei). Single crystal X-ray diffraction analysis of compound **3d** was performed on an automatic four-circle diffractometer Agilent Super Nova, Dual, Cu at zero, Atlas S2. High-resolution mass spectra (HRMS) were registered with a Bruker MaXis Impact (Bruker Daltonics, Bremen, Germany) spectrometer (electrospray ionization; HCO_2_Na–HCO_2_H was used for calibration). The samples were dissolved in MeCN under moderate heating (37–38 °C) and ultrasonication. See Electronic Supplementary Material file for NMR, FTIR and HRMS spectral charts and X-ray analysis data.

FT-IR spectra were measured on a Bruker Vertex 70 instrument (Bruker Optics GmbH & Co. KG, Ettlingen, Germany) equipped with an ATR sampling module. Elemental analyses were carried out using a Carlo Erba 1106 Elemental Analyzer (Carlo Erba Strumentazione, Cornaredo, Italy). Reaction progress and purity of isolated compounds were controlled via TLC on Sorbfil-A plates (Imid Ltd., Krasnodar, Russia), and the eluent was acetone:hexane 2:1 or ethyl acetate. Developed TLC plates were stained with UV light and iodine vapors.

N,N′-Diphenyldithiomalondiamide (dithiomalondianilide) **1** was prepared from acetylacetone and phenyl isothiocyanate as reported in [33,34]. (*E*)-3-Aryl-2-cyanoacrylamides **2a-f** were prepared via reaction of 2-cyanoacetamide with aromatic aldehydes in the presence of catalytic amounts of morpholine in EtOH at 40–50 °C [89] or in water in the presence of surfactants [90]. All other reagents and solvents were purchased from commercial vendors (BioInLabs, Rostov-on-Don, Russia) and used as received.

**General procedure for the preparation of 6-amino-4-aryl-7-phenyl-3-(phenyl- imino)-4,7-dihydro-3H-[1,2]dithiolo[3,4-b]pyridine-5-carboxamides 3a-f (Method A)**. A vial was charged, under air, with dithiomalondianilide **1** (500 mg, 1.75 mmol), 3-aryl-2-cyanoacrylamide **2a-f** (1.75 mmol) and EtOH (15 mL). The mixture was treated with morpholine (0.23 mL, 1.75 mmol) at 40–50 °C (internal temperature) under vigorous stirring. Complete dissolution of starting materials and formation of a deep-yellow solution occurred for a very short time (a matter of minutes). Usually, a yellow precipitate begins to separate within half an hour. The reaction mixture was stirred for another 2–3 h (TLC control) and left to stand at room temperature until crystallization was complete. The precipitated product was filtered off, washed with EtOH and purified either via flash chromatography (silica gel, eluent–acetone) or recrystallization from acetone or acetone–EtOH mixtures to yield pure dithiolopyridines **3a-f** as yellow crystalline solids.

**General procedure for the preparation of 6-amino-4-aryl-7-phenyl-3-(phenyl- imino)-4,7-dihydro-3H-[1,2]dithiolo[3,4-b]pyridine-5-carboxamides 3a-f (Method B)**. Morpholine (0.23 mL, 1.75 mmol) was added to a mixture of 2-cyanoacetamide (150 mg, 1.75 mmol) and substituted benzaldehyde (1.75 mmol) in EtOH (6–8 mL). The reaction mixture was stirred at ~60 °C for 5 min, and then dithiomalondianilide **1** (500 mg, 1.75 mmol) and EtOH (10 mL) were added. The resulting mixture was stirred at 40–50 °C for 2–3 h and monitored via TLC until the starting reagents were consumed. After the reaction was complete, the reaction mixture was left to stand at room temperature for complete crystallization. The precipitated product was filtered off, washed with EtOH and purified as described above to yield pure dithiolopyridines **3a-f** as yellow crystalline solids.

*6-Amino-4-(2-nitrophenyl)-7-phenyl-3-(phenylimino)-4,7-dihydro-3H-[1,2]dithiolo[3,4-b]-pyridine-5-carboxamide* (**3a**). The yield was 54% (method A) or 43% (method B). ^1^H NMR (400 MHz, DMSO-*d*_6_): 5.64 (s, 1H, H-4), 6.63 (br s, 2H, NH_2_), 6.73 (d, ^3^J = 7.5 Hz, 2H, H-2 H-6 Ph), 7.03–7.07 (m, 1H, H-4 Ph), 7.28–7.32 (m, 2H, H-3 H-5 Ph), 7.44–7.49 (m, 1H, H-4 Ar), 7.58 (br s, CONH_2_), 7.64–7.69 (m, 5H, Ph), 7.78–7.83 (m, 3H, Ar). ^13^C NMR (101 MHz, DMSO-*d*_6_): 34.6 (C-4), 77.6 (C-5), 111.1 (C-3a), 120.0 (2C, C-2 C-6 Ph), 123.4 (C-3 Ar), 124.2 (C-4 Ph), 127.9 (C-4 Ar), 129.4 (2C, C-3 C-5 Ph), 130.2 (CH Ph), 130.4 (CH Ph), 131.0 (C-6 Ar), 134.2 (C-5 Ar), 135.5 (C-1 Ph), 140.0 (C-1 Ar), 148.5 (C-NO_2_), 150.5 (C-1 Ph), 152.0 (C-6), 154.4 (C-7a), 163.3 (C-3), 171.1 (CONH_2_). FTIR, ν_max_, cm^−1^: 3439, 3350, 3171, 3119, 3063 (N-H, C-H); 1668 (C=O); 1645 (C=N); 1533 (NO_2 asym_); 1357 (NO_2 sym_). HRMS (ESI) *m/z*: calculated for C_25_H_20_N_5_O_3_S_2_ [M + H]^+^: 502.1008, found 502.1023 (Δ 2.99 ppm).

*6-Amino-4-(4-chlorophenyl)-7-phenyl-3-(phenylimino)-4,7-dihydro-3H-[1,2]dithiolo[3,4-b]pyridine-5-carboxamide* (**3b**). The yield was 40% (method A) or 33% (method B). ^1^H NMR (400 MHz, DMSO-*d*_6_): 5.24 (s, 1H, H-4), 6.55 (br s, 2H, NH_2_), 6.84 (d, ^3^*J* = 7.5 Hz, 2H, H-2 H-6 Ph), 7.07–7.10 (m, 1H, H-4 Ph), 7.30–7.39 (m, 6H, H-3 H-5 Ph, 2H Ar, CONH_2_ overlapped), 7.43 (d, ^3^*J* = 8.3 Hz, 2H, H Ar), 7.60–7.67 (m, 5H, Ph). ^13^C NMR (101 MHz, DMSO-*d*_6_): 38.6 (C-4), 78.0 (C-5), 112.4 (C-3a), 120.0 (2C, C-2 C-6 Ph), 124.2 (C-4 Ph), 127.8 (2C, CH Ar), 129.5 (2C, CH Ar), 129.7 (2C, C-3 C-5 Ph), 130.1 (CH Ph), 130.5 (CH Ph), 131.0 (CH Ph), 135.4 (C–Cl), 135.6 (C-1 Ph), 146.6 (C-1 Ar), 150.8 (C-1 Ph), 152.0 (C-6), 154.5 (C-7a), 163.1 (C-3), 171.3 (CONH_2_). FTIR, ν_max_, cm^−1^: 3406, 3300, 3151, 3055 (N-H, C-H); 1662 (C=O, C=N). HRMS (ESI) *m/z*: calculated for C_25_H_20_ClN_4_OS_2_ [M + H]^+^: 491.0767, found 491.0780 (Δ 2.65 ppm).

*6-Amino-4-(3-nitrophenyl)-7-phenyl-3-(phenylimino)-4,7-dihydro-3H-[1,2]dithiolo[3,4-b]pyridine-5-carboxamide* (**3c**). The yield was 37% (method A) or 27% (method B). ^1^H NMR (400 MHz, DMSO-*d*_6_): 5.32 (s, 1H, H-4), 6.58 (br s, 2H, NH_2_), 6.89 (d, ^3^*J* = 7.5 Hz, 2H, H-2 H-6 Ph), 7.06–7.10 (m, 1H, H-4 Ph), 7.32–7.36 (m, 2H, H-3 H-5 Ph), 7.40 (br s, CONH_2_), 7.58–7.67 (m, 6H, H-5 Ar + Ph), 8.01 (br d, ^3^*J* = 7.5 Hz, H-6 Ar), 8.08 (dd, ^3^*J* = 8.2 Hz, ^3^*J* = 1.4 Hz, H-4 Ar), 8.58–8.59 (m, 1H, H-2 Ar). ^13^C NMR (101 MHz, DMSO-*d*_6_): 38.7 (C-4), 78.7 (C-5), 111.2 (C-3a), 120.0 (2C, C-2 C-6 Ph), 121.3 (C-4 Ar), 122.6 (C-2 Ar), 124.3 (C-4 Ph), 129.4 (C-5 Ar), 129.7 (2C, C-3 C-5 Ph), 130.0 (CH Ph), 130.4 (CH Ph), 130.9 (CH Ph), 134.1 (C-6 Ar), 135.7 (C-1 Ph), 147.3 (C-1 Ar), 148.0 (C-NO_2_), 150.9 (C-1 Ph), 151.4 (C-6), 154.7 (C-7a), 163.2 (C-3), 171.2 (CONH_2_). FTIR, ν_max_, cm^−1^: 3493, 3458, 3444, 3402, 3342, 3211, 3057 (N-H, C-H); 1647 (C=O, C=N), 1575 (NO_2 asym_); 1348 (NO_2 sym_). HRMS (ESI) *m/z*: calculated for C_25_H_20_N_5_O_3_S_2_ [M + H]^+^: 502.1008, found 502.1023 (Δ 2.99 ppm).

*6-Amino-4-(2,4-dichlorophenyl)-7-phenyl-3-(phenylimino)-4,7-dihydro-3H-[1,2]dithiolo[3,4-b]pyridine-5-carboxamide* (**3d**). The yield was 39% (method A) or 31% (method B). According to NMR, after recrystallization from acetone–EtOH mixture the product appeared as solvate **3d**:EtOH = 2:1. ^1^H NMR (400 MHz, DMSO-*d*_6_): 1.05 (t, ^3^*J* = 6.8 Hz, 3H, EtOH), 3.43–3.46 (m, 2H, EtOH), 4.33 (t, ^3^*J* = 5.0 Hz, 1H, EtOH), 5.31 (s, 1H, H-4), 6.26 (br s, 2H, NH_2_), 6.79 (d, ^3^*J* = 7.5 Hz, 2H, H-2 H-6 Ph), 7.02–7.06 (m, 1H, H-4 Ph), 7.28–7.31 (m, 4H, H-3 H-5 Ph, CONH_2_), 7.39 (dd, ^3^*J* = 8.2 Hz, ^4^*J* = 1.7 Hz, H-5 Ar), 7.48 (d, ^4^*J* = 1.7 Hz, H-3 Ar), 7.60–7.64 (m, 5H, Ph), 7.73 (d, ^3^*J* = 8.2 Hz, H-6 Ar). ^13^C NMR (101 MHz, DMSO-*d*_6_): 18.5 (CH_3_ EtOH), 38.7 (C-4), 56.0 (CH_2_ EtOH), 77.9 (C-5), 109.8 (C-3a), 120.0 (2C, C-2 C-6 Ph), 124.1 (C-4 Ph), 126.9 (C-5 Ar), 128.6 (C-3 Ar), 129.5 (2C, C-3 C-5 Ph), 130.29 (CH Ph), 130.32 (CH Ph), 130.9 (CH Ph), 131.4 (C-4 Ar), 133.0 (C–Cl Ar), 133.9 (C-6 Ar), 135.6 (C-1 Ph), 141.4 (C-1 Ar), 150.3 (C-1 Ph), 151.0 (C-6), 154.9 (C-7a), 162.1 (C-3), 171.3 (CONH_2_). FTIR, ν_max_, cm^−1^: 3473, 3363, 3333, 3144, 3051 (N-H, C-H); 1707 (C=O), 1633 (C=N). HRMS (ESI) *m/z*: calculated for C_25_H_18_Cl_2_N_4_NaOS_2_ [M + Na]^+^: 547.0197, found 547.0174 (Δ 4.2 ppm).

*6-Amino-4-(3,4-dimethoxyphenyl)-7-phenyl-3-(phenylimino)-4,7-dihydro-3H-[1,2]dithiolo[3,4-b]pyridine-5-carboxamide* (**3e**). The yield was 40% (method A) or 22% (method B). ^1^H NMR (400 MHz, DMSO-*d*_6_): 3.79 (s, 3H, MeO–Ar), 3.84 (s, 3H, MeO–Ar), 5.00 (s, 1H, H-4), 6.30 (br s, 2H, NH_2_), 6.82–6.90 (m, 5H, H Ph, H Ar), 7.06–7.10 (m, 1H, H-4 Ph), 7.28–7.33 (m, 4H, H-3 H-5 Ph, CONH_2_), 7.55–7.66 (m, 5H, H Ar).^13^C NMR (101 MHz, DMSO-*d*_6_): 38.0 (C-4), 54.9 (ArOCH_3_), 55.0 (ArOCH_3_), 79.1 (C-5), 110.8 (C-3a), 111.2 (CH Ar), 112.0 (CH Ar), 118.9 (CH Ar), 120.1 (2C, C-2 C-6 Ph), 124.3 (C-4 Ph), 129.6 (2C, C-3 C-5 Ph), 129.9 (2C, CH Ph), 130.4 (2C, CH Ph), 130.9 (C-4 Ph), 135.5 (C-1 Ph), 137.2 (C-1 Ar), 147.7 (C–OMe), 148.3 (C–OMe), 150.4 (C-1 Ph), 151.5 (C-6), 154.0 (C-8a), 163.0 (C=N), 171.1 (C=O). FTIR, ν_max_, cm^−1^: 3475, 3388, 3267, 3178, 3047 (N-H, C-H); 1668 (C=O), 1633 (C=N). Elemental analysis (C_27_H_24_N_4_O_3_S_2_, M 516.63): calculated (%): C, 62.77; H, 4.68; N, 10.84; found (%): C, 62.57; H, 4.90; N, 10.79.

*6-Amino-4-(4-methoxyphenyl)-7-phenyl-3-(phenylimino)-4,7-dihydro-3H-[1,2]dithiolo[3,4-b]pyridine-5-carboxamide* (**3f**). The yield was 54% (method A) or 35% (method B). ^1^H NMR (400 MHz, DMSO-*d*_6_): 3.74 (s, 3H, MeO–Ar), 4.94 (s, 1H, H-4), 6.26 (br s, 2H, NH_2_), 6.81 (d, ^3^*J* = 7.5 Hz, 2H, H-2 H-6 Ph), 6.93 (d, ^3^*J* = 8.4 Hz, 2H, H-Ar), 7.04–7.08 (m, 1H, H-4 Ph), 7.29–7.37 (m, 6H, H-3 H-5 Ph, H Ar, CONH_2_), 7.55–7.63 (m, 5H, H Ar). ^13^C DEPTQ NMR (101 MHz, DMSO-*d*_6_): 37.9 (C-4), 54.9 (OCH_3_), 78.0 (C-5), 113.0 (C-3a), 113.5 (2C, CH Ar), 120.1 (2C, C-2 C-6 Ph), 124.3 (C-4 Ph), 128.0 (2C, CH Ar), 129.5 (2C, C-3 C-5 Ph), 130.0 (2C, CH Ph), 130.3 (2C, CH Ph), 131.0 (C-4 Ph), 135.5 (C-1 Ph), 138.9 (C-1 Ar), 150.7 (C-1 Ph), 152.0 (C-6), 153.9 (C-8a), 157.5 (C–OMe), 163.1 (C=N), 171.1 (C=O). HRMS (ESI) *m/z*: calculated for C_26_H_22_N_4_O_2_S_2_ [M + H]^+^: 487.1262, found 487.1276 (Δ 2.87 ppm).

### 3.1. X-ray Studies for Single Crystals of ***3d***

Single crystals of 6-amino-4-(2,4-dichlorophenyl)-7-phenyl-3-(phenylimino)- 4,7-dihydro-3H-[1,2]dithiolo[3,4-b]pyridine-5-carboxamide **3d** (C_25_H_18_Cl_2_N_4_OS_2_, M 525.45 g/mol) were prepared via slow evaporation of saturated solution in DMSO. The crystal was kept at 100.01(10) K during data collection. Using Olex2 [91], the structure was solved with the olex2.solve structure solution program using Charge Flipping and refined with the SHELXL [92] package using Gauss–Newton minimisation.

The crystals are monoclinic, space group *I*2/a (no. 15), at 100.01(11) K: *a* = 11.97350(10) Å, *b* = 12.96920(10) Å, *c* = 34.4739(2) Å, α = 90°, *β* = 90.7890(10)°, γ = 90°, *V* = 5352.83(7) Å^3^, *Z* = 8, *μ*(Cu Kα) = 3.837 mm^−1^, *D_calc_* = 1.304 g/cm^3^, *F*(000) = 2160.0, 29,542 reflections measured (7.282° ≤ 2Θ ≤ 153.23°) and 5572 unique (*R*_int_ = 0.0263, R_sigma_ = 0.0158); these values were used in all calculations. The final *R*_1_ was 0.0352 (*I* > 2σ(*I*)) and *wR*_2_ was 0.0939 (all data).

A full set of crystallographic data has been deposited in the Cambridge Crystallographic Data Center (CCDC **2310349**).

### 3.2. Herbicide Safening Effect Studies

Germinated sunflower seeds (cv. Master) with 2–4 mm long embryo roots were placed in a solution of 2,4-D (10^–3^% by weight) for 1 h to achieve 40–60% inhibition of hypocotyls’ growth. After treatment, the seedlings were washed with pure water and placed into a solution of the corresponding compounds **3b**, **3d** and **3f** (concentrations 10^–2^, 10^–3^, 10^–4^ or 10^–5^% by weight, “herbicide + antidote” experiments). After 1 h, the seedlings were washed with pure water and placed on paper stripes (10 × 75 cm, 20 seeds per stripe). The stripes were rolled and placed into beakers with water (50 cm^3^). The reference group of seedlings (“herbicide” experiments) was kept in 2,4-D solution (10^–3^%) for 1 h and then in water for 1 h. The “control” seedlings were kept in water for 2 h. The temperature of all solutions was maintained at 28 °C. The seedlings were then thermostated for 3 days at 28 °C. Each experiment was performed in triplicate, and 20 seeds were used in each experiment. The results are given in Table 2.

## 4. Conclusions

In summary, we have synthesized new [1,2]dithiolo[3,4-b]pyridine-5-carboxamides using a base-promoted reaction of dithiomalondianilide with 3-aryl-2-cyanoacrylamides. The structure of the compounds was confirmed using spectral data and X-ray diffraction analysis. The mechanism of formation of the dithiolopyridine bicyclic ring system was examined in detail through DFT calculations with Grimme’s B97-3c composite scheme based on the combination of the B97 GGA functional and the def2-mTZVP basis set with the D3BJ dispersion correction. It was shown that the rate-limiting step is the closure of the pyridine ring, whereas further oxidation and the closure of 1,2-dithiol ring with oxygen in the air results in a lower energy barrier.

The calculation of ADMET parameters for the new compounds was carried out. It was shown that dithiolopyridines **3** do not fully meet the criteria of oral bioavailability. The results of molecular docking studies and a search for possible protein targets for new [1,2]dithiolo[3,4-b]pyridine-5-carboxamides show that the compounds are of interest as promising antitumor agents or as regulators of blood coagulation factor VII. Finally, in contrast to close structural analogs, [1,2]dithiolo[3,4-b]pyridine-5-carboxamides **3** were found to exhibit only moderate herbicide safening effects against 2,4-D in the laboratory experiments with sunflower seedlings.

## Figures and Tables

**Table 1:** The structure and yields of compounds **3**.

Entries	Product	Methods	Yields, %
entry 1	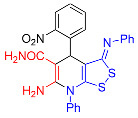 **3a**	A	54
		B	43
entry 2	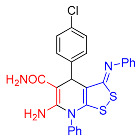 **3b**	A	40
		B	33
entry 3	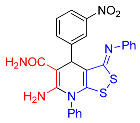 **3c**	A	37
		B	27
entry 4	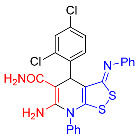 **3d**	A	39
		B	31
entry 5	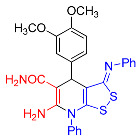 **3e**	A	40
		B	22
entry 6	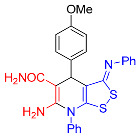 **3f**	A	54
		B	35

**Table 2:** The antidote effects of the compounds **3b**, **3d** and **3f** with respect to herbicide 2,4-D.

N	Compound	Organ	Antidote Effect *A* at Different Concentrations, % ^1^			
			10^−2^	10^−3^	10^−4^	10^−5^
1	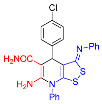 **3b**	roots	123	121	121	116
		hypocotyls	127	130	124	120
2	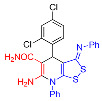 **3d**	roots	130	131	123	128
		hypocotyls	132	127	120	128
3	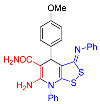 **3f**	roots	133	127	132	130
		hypocotyls	132	132	116	119

^1^ The differences are reliable at *p* = 0.95.

## Data Availability

The Electronic Supplementary Material.pdf file containing X-ray crystallography data and the ^1^H and ^13^C NMR, 2D NMR ^1^H-^13^C HSQC and ^1^H-^13^C HMBC, FTIR and HRMS spectral charts (Figures S1–S22, Tables S1–S25) can be found in the Supplementary Materials.

## Supplementary Materials

The following supporting information can be downloaded at https://www.mdpi.com/article/10.3390/ijms25020769/s1.
