# Supplementary material for: 6-Amino-4-aryl-7-phenyl-3-(phenylimino)-4,7-dihydro-3H-[1,2]dithiolo[3,4-b]pyridine-5-carboxamides: Synthesis, Biological Activity, Quantum Chemical Studies and In Silico Docking Studies
Source: Int J Mol Sci. 2024 Jan 7;25(2):769. doi: 10.3390/ijms25020769 (PMC10815501; doi:10.3390/ijms25020769)
Supplement: Supplementary file 1 [file ijms-25-00769-s001.zip › ijms-2782856-supplementary.pdf]

Electronic Supplementary Material file

6-Amino-4-aryl-7-phenyl-3-(phenylimino)-4,7-dihydro-3H-[1,2]dithiolo[3,4-b]pyridine-5-carboxamides: synthesis, biological activity, quantum chemical studies and *in silico* docking studies

Victor V. Dotsenko [1,2,]*, Alexander V. Bespalov [1,]*, Anna E. Sinotsko [1], Azamat Z. Temerdashev [3], Vladimir K. Vasilin [4], Ekaterina A. Varzieva [1], Vladimir D. Strelkov [1], Nicolai A. Aksenov [2] and Inna V. Aksenova [2]

[1]Department Organic Chemistry and Technologies, Kuban State University, 149 Stavropolskaya St., 350040 Krasnodar, Russia;
[2]Department of Chemistry, North Caucasus Federal University, 1a Pushkin St., 355017 Stavropol, Russia;
[3]Department of Analytical Chemistry, Kuban State University, 149 Stavropolskaya St., 350040 Krasnodar, Russia;
[4]Department of Bioorganic Chemistry, Kuban State Technological University, 2 Moskovskaya St., 350072, Krasnodar, Russia.
*****Correspondence: victor_dotsenko_@mail.ru (V.V.D.), bespalov-alex@mail.ru (A.V.B.).

Contents

Scheme S1. The reactions involving unsaturated nitriles both as building blocks for pyridine synthesis and as oxidizing agents 3
Figure S1. ^{1}H NMR spectrum of compound 3a, DMSO-d_6 (400 MHz) 4
Figure S2. ^{13}C NMR spectrum of 4
Figure S3. FTIR spectrum of compound 3a 5
Figure S4. HSQC 2D ^{1}H-^{13}C NMR spectrum of compound 3a, DMSO-d_6 (400/101 MHz) 5
Figure S5. HMBC 2D ^{1}H-^{13}C NMR spectrum of compound 3a, DMSO-d_6 (400/101 MHz) 6
Table S1. The observed correlations in the ^{1}H-^{13}C HSQC and ^{1}H-^{13}C HMBC 2D NMR spectra of compound 3a 7
Figure S6. ^{1}H NMR spectrum of compound 3c, DMSO-d_6 (400 MHz) 8
Figure S7. ^{13}C NMR spectrum of compound 3c, DMSO-d_6 (101 MHz) 8
Figure S8. ^{1}H–^{13}C HSQC NMR spectrum of compound 3c, DMSO-d_6 (400/101 MHz) 9
Table S2. The observed correlations in the ^{1}H-^{13}C HSQC 2D NMR spectrum of compound 3c 10
Figure S9. FTIR spectrum of compound 3c 11
Figure S10. ^{1}H NMR spectrum of compound 3d (solvate with EtOH), DMSO-d_6 (400 MHz)11
Figure S11. ^{13}C NMR spectrum of compound 3d (solvate with EtOH), DMSO-d_6 (101 MHz)12
Figure S13. ^{1}H–^{13}C HSQC NMR spectrum of compound 3d (solvate with EtOH), DMSO-d_6 (100/400 MHz) 13
Figure S14. ^{1}H–^{13}C HMBC NMR spectrum of compound 3d (solvate with EtOH), DMSO-d_6 (100/400 MHz) 14
Table S3. The observed correlations in the ^{1}H-^{13}C HSQC and ^{1}H-^{13}C HMBC 2D NMR spectra of compound 3d (solvate with EtOH), DMSO-d_6 (100/400 MHz) 15
Figure S15. FTIR spectrum of compound 3d 16
Figure S16. ORTEP drawings of the crystal structure showing 50% probability thermal ellipsoids (CCDC 2310349) and microphotography of the single crystal of compound 3d used for X-Ray diffraction analysis at the bottom 17

Table S4. Crystal data and structure refinement for 6-amino-4-(2,4-dichlorophenyl)-7-phenyl-3-(phenylimino)-4,7-dihydro-3H-[1,2]dithiolo[3,4-b]pyridine-5-carboxamide 3d .. 18
Table S5. Fractional Atomic Coordinates (×10^4) and Equivalent Isotropic Displacement Parameters (Å^2×10^3) for compound 3d. U_{eq} is defined as 1/3 of of the trace of the orthogonalised U_{IJ} tensor. 19
Table S6. Anisotropic Displacement Parameters (Å^2×10^3) for 3d. The Anisotropic displacement factor exponent takes the form: $-2\pi^2[h^2a^{*2}U_{11}+...+2hka^*b^*U_{12}+ …]$ 20
Table S7. Bond Lengths for compound 3d. 21
Table S8. Bond Angles for compound 3d. 22
Table S9. Torsion Angles for compound 3d 23
Table S10. Hydrogen Atom Coordinates (Å×10^4) and Isotropic Displacement Parameters (Å^2×10^3) for compound 3d. 24
Table S11. Solvent masks information for compound 3d 25
Crystal structure determination details for compound 3d 25
Figure S17. HRMS data for compound 3a 26
Figure S18. HRMS data for compound 3b 26
Figure S19. HRMS data for compound 3c 27
Figure S20. HRMS data for compound 3d 27
Figure S21. HRMS data for the crude mixture from the experiment (3) - reaction of 3-(4-methoxyphenyl)-2-cyanoacrylamide 2f with dithiomalondianilide 1 taken in the ratio 2 : 1;28
Figure S22. HRMS of pure dithiolopyridine 3f (experiment (2), Table 1, entry 6). 28
Table S12. Toxicity risks and physicochemical parameters of compounds 3a-f predicted using OSIRIS Property Explorer 29
Table S13. ADMET parameters calculated for compound 3a using SwissADME and AdmetSar 31
Table S14. ADMET parameters calculated for compound 3b using SwissADME and AdmetSar 35
Table S15. ADMET parameters calculated for compound 3c using SwissADME and AdmetSar 39
Table S16. ADMET parameters calculated for compound 3d using SwissADME and AdmetSar 42
Table S17. ADMET parameters calculated for compound 3e using SwissADME and AdmetSar 45
Table S18. ADMET parameters calculated for compound 3f using SwissADME and AdmetSar 48
Table S19. Rat acute toxicity of 3a predicted by GUSAR 52
Table S20. Rat acute toxicity of 3b predicted by GUSAR 53
Table S21. Rat acute toxicity of 3c predicted by GUSAR 54
Table S22. Rat acute toxicity of 3d predicted by GUSAR 55
Table S23. Rat acute toxicity of 3e predicted by GUSAR 56
Table S24. Rat acute toxicity of 3f predicted by GUSAR 57
Table S25. The predicted results of protein-ligand interaction for compounds 3a-f 58

Scheme S1. The reactions involving unsaturated nitriles both as building blocks for pyridine synthesis and as oxidizing agents

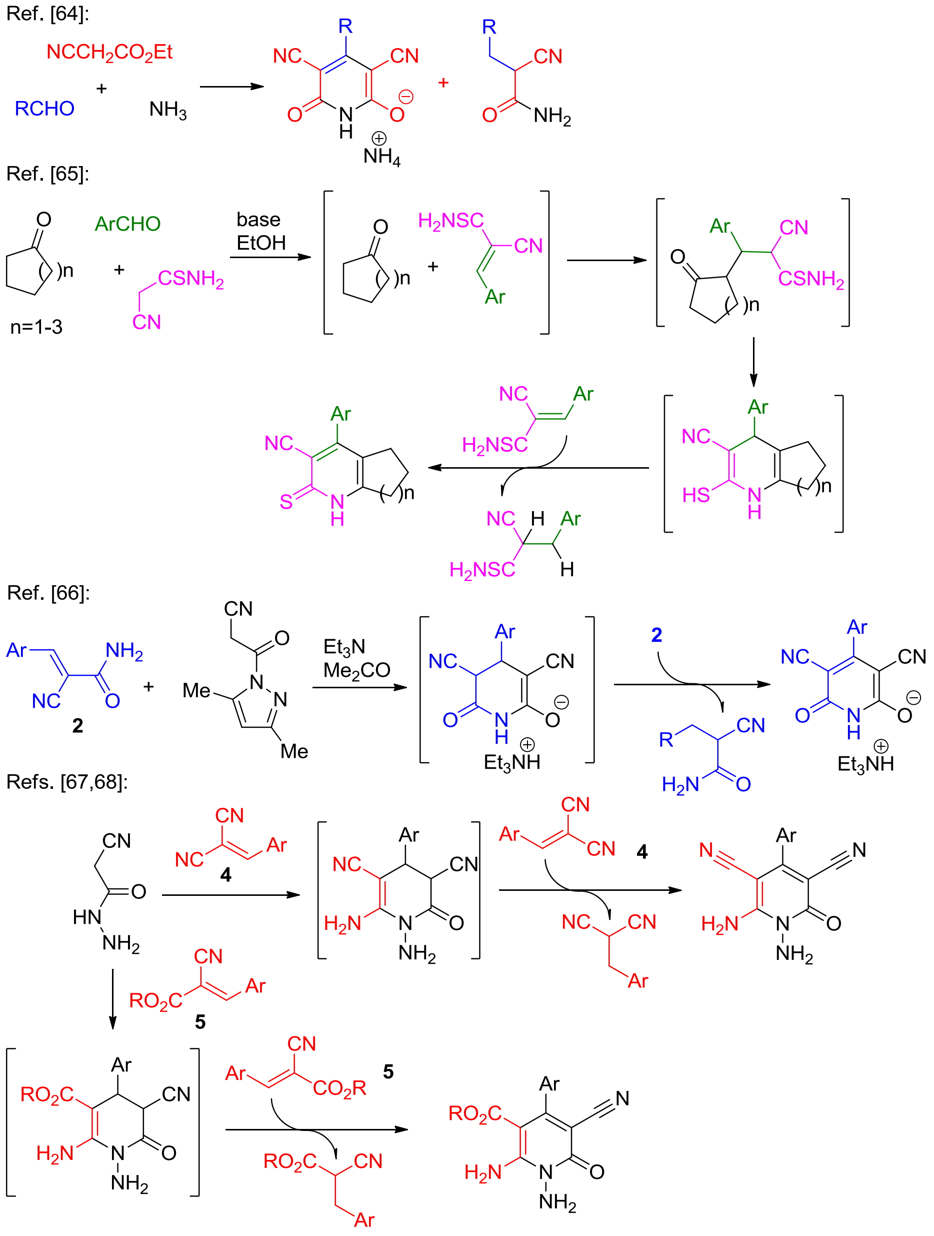

In a number of studies concerning the synthesis of pyridine derivatives starting from unsaturated nitriles (e.g., arylmethylidene malononitriles **4**) it was shown that partially saturated pyridine intermediate easily reacts with unsaturated nitriles to give α,β-saturated nitriles and corresponding oxidation products – nicotinonitriles [64-68].

64. Brunskill, J.S.A. Some cyano-amides and dicyano-glutaconimides derived from pyridine aldehydes. *J. Chem. Soc. Perkin Trans. 1*, **1972**, 2946-2950.

65. Shestopalov, A.M.; Rodinovskaya, L.A.; Zubarev, A.A.; Nesterov, V.N.; Ugrak, B.I.; Dutova, T.Y. Synthesis and domino reactions of polymethylene-3-cyanopyridine-2 (1H)-thiones. *J. Heterocycl. Chem.* **2020**, *57*(2), 913-922.

66. Chigorina, E.A. 1-(Cyanoacetyl)-3,5-dimethylpyrazole as an effective alternative to cyanoacetic ester in the synthesis of 2, 6-dioxopiperidine-3, 5-dicarbonitrile derivatives. *Chem. Heterocycl. Comp.* **2013**, *49*, 574-585.

67. Soto, J.L.; Seoane, C.; Zamorano, P.; Cuadrado, F.J. A convenient synthesis of N-amino-2-pyridones. *Synthesis* **1981**, *1981*(07), 529-530.

68. Seoane, C.; Soto, J.L.; Zamorano, P. Preparation of substituted 1,6-diamino-2-oxopyridines. *Org. Prep. Proced. Int.* **1984**, *16*(6), 393-400.

Figure S1. ^{1}**H NMR spectrum of compound 3a, DMSO-d$_6$ (400 MHz)**

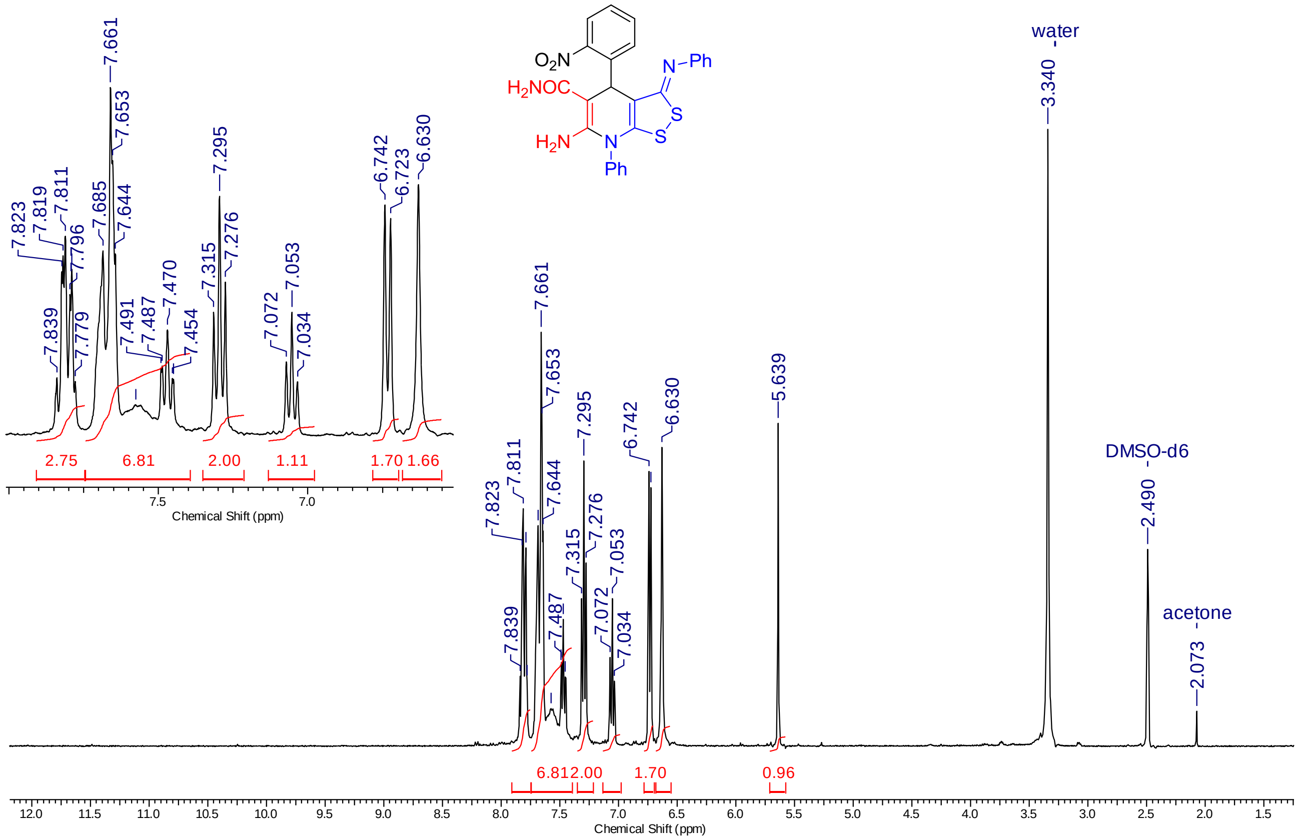

Figure S2. ^{13}**C NMR spectrum of compound 3a, DMSO-d$_6$ (101 MHz)**

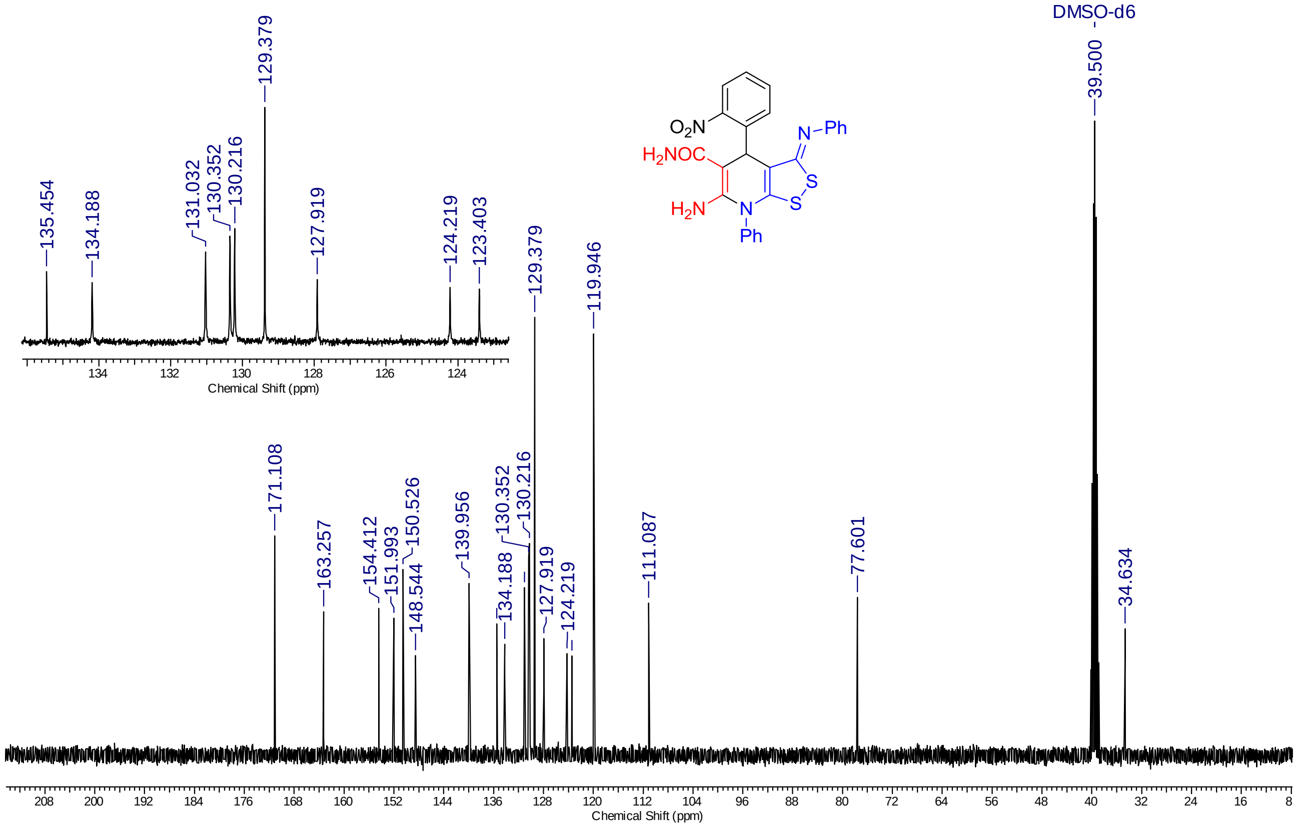

Figure S3. FTIR spectrum of compound 3a

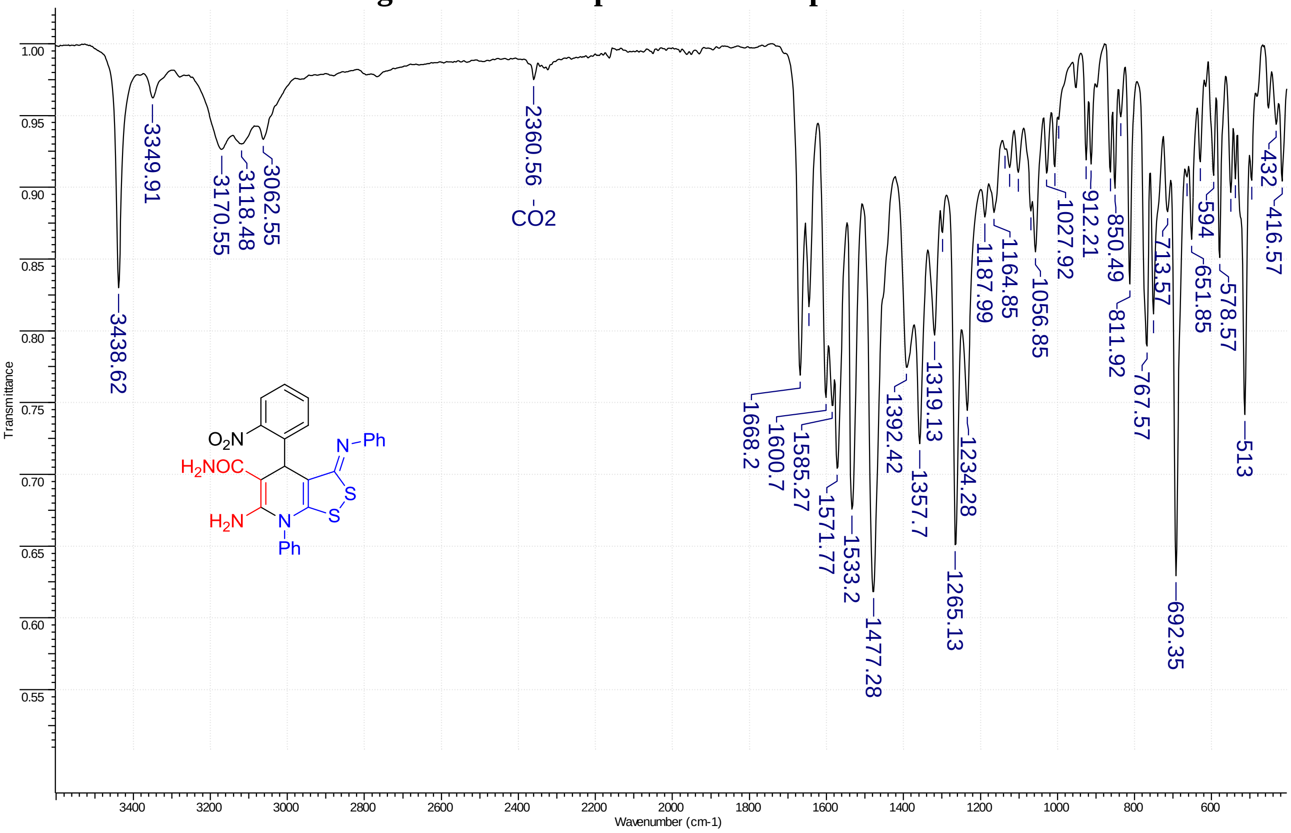

Figure S4. HSQC 2D ^{1}H-^{13}C NMR spectrum of compound 3a, DMSO-d_6 (400/101 MHz)

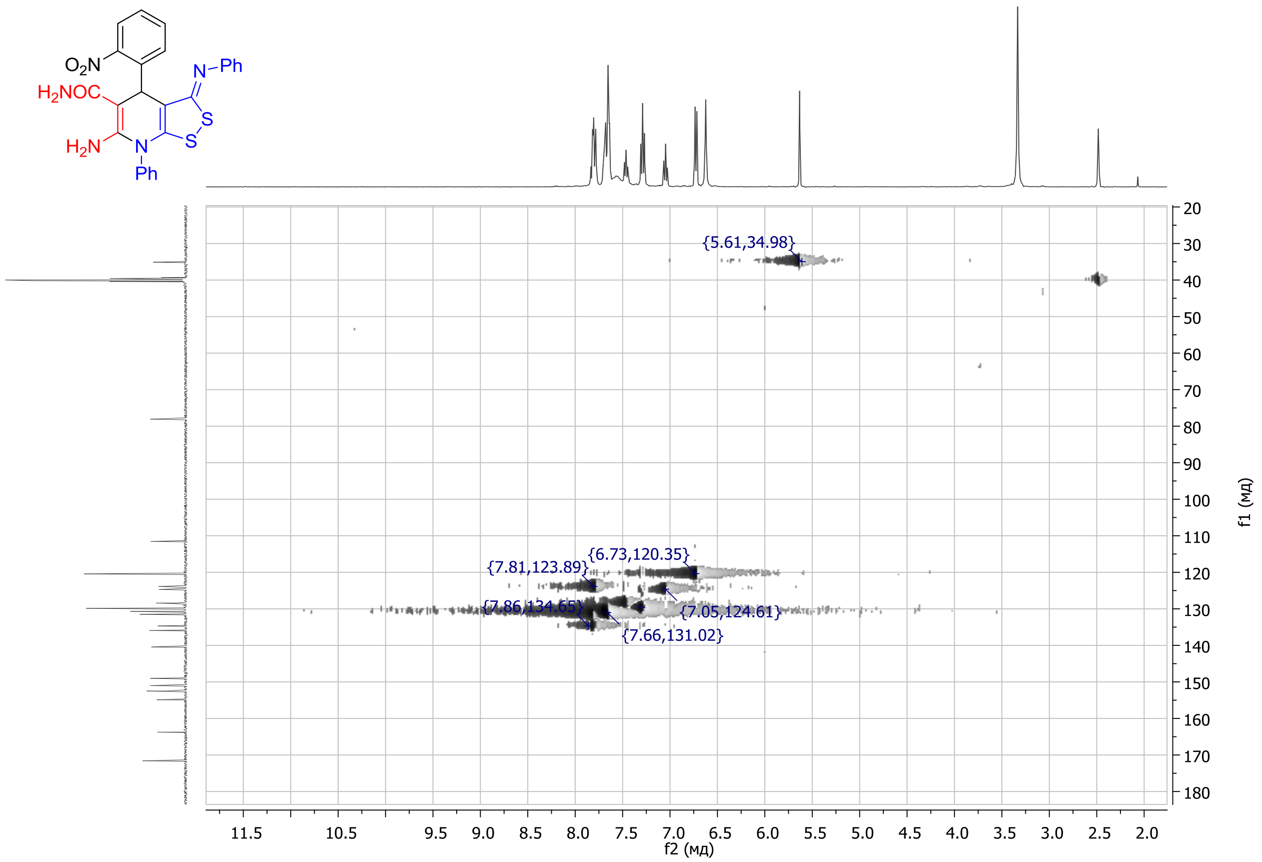

Figure S5. HMBC 2D ^{1}H-^{13}C NMR spectrum of compound 3a, DMSO-d_6 (400/101 MHz)

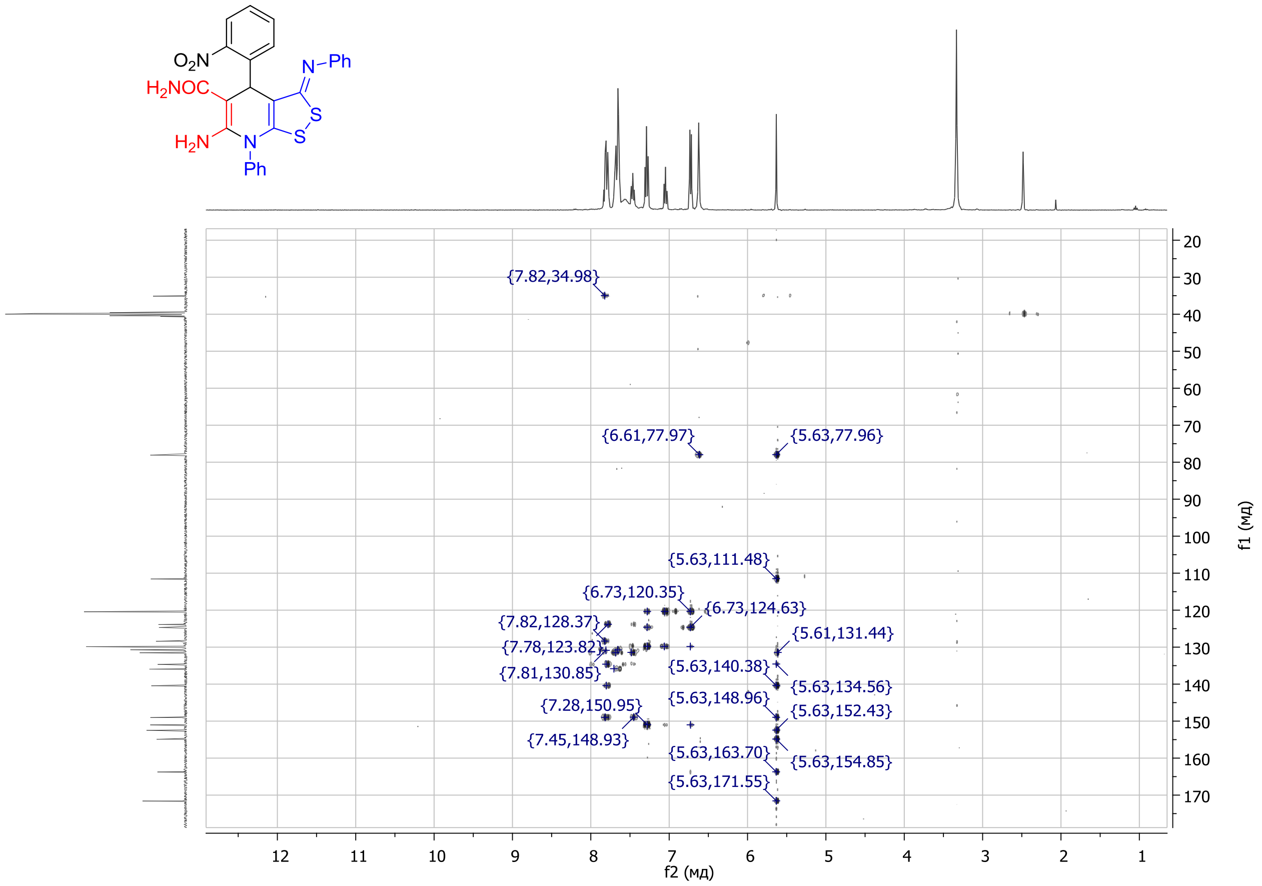

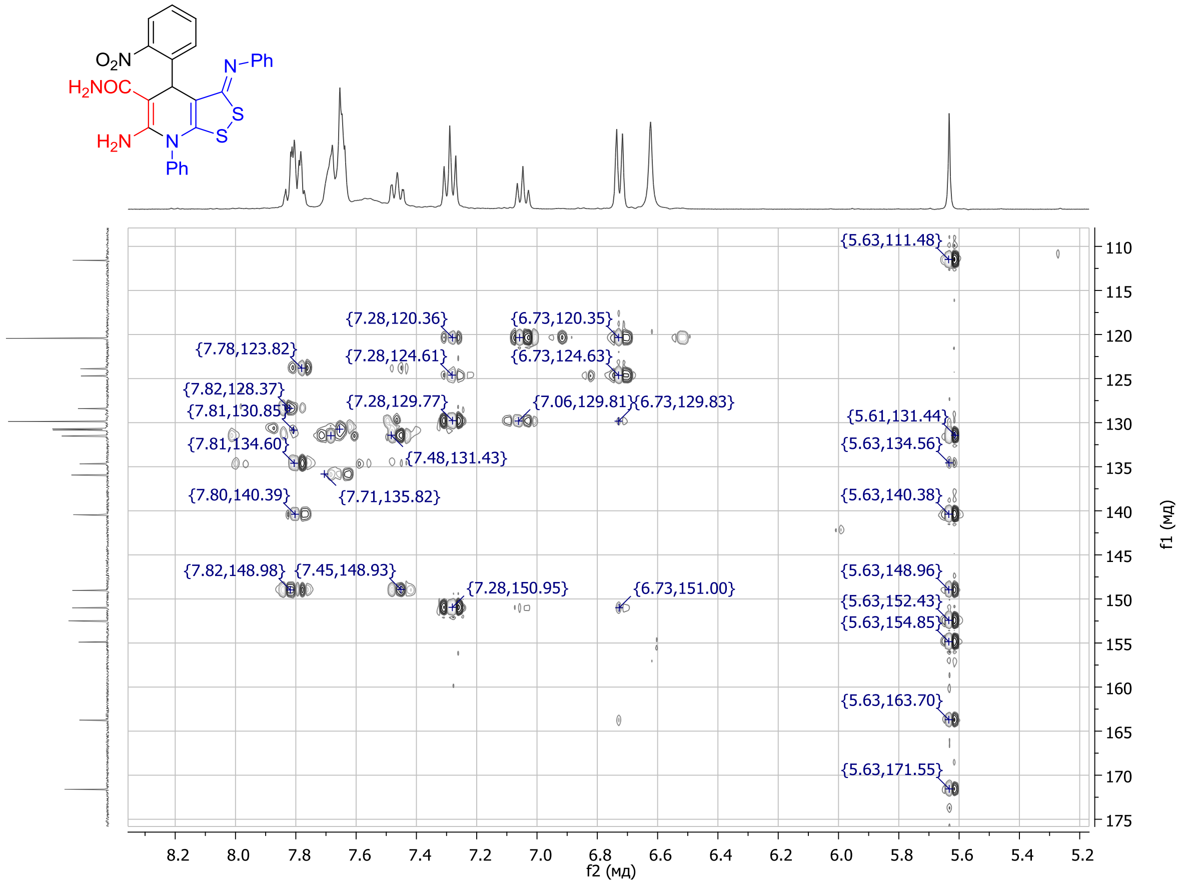

Table S1. The observed correlations in the ^{1}H-^{13}C HSQC and ^{1}H-^{13}C HMBC 2D NMR spectra of compound 3a

^{13}C chemical shifts are given in **red**, ^{1}H shifts – in **blue**

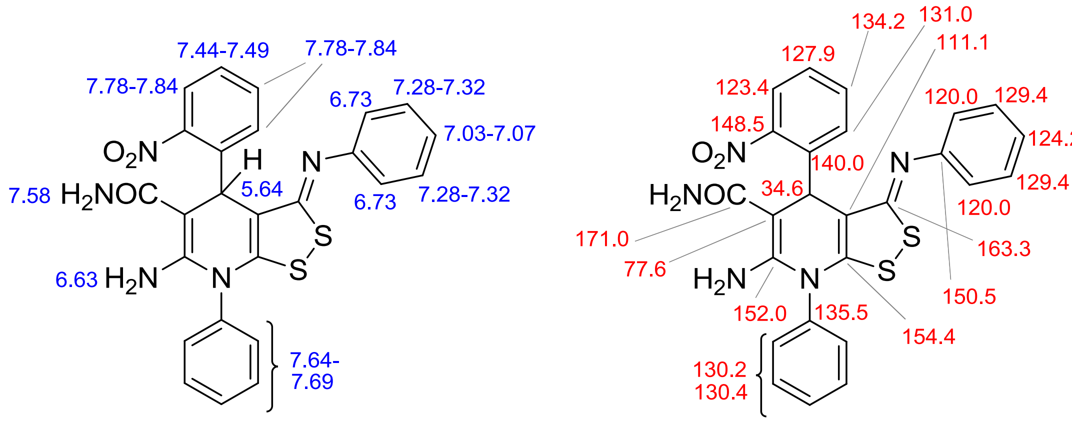

^{1}H NMR shifts, δ, ppm	Correlations in HSQC spectrum, δ, ppm	Correlations in HMBC spectrum, δ, ppm
5.64 (s, 1H, H-4)	34.6 (C-4)	77.6 (C-5), 111.1 (C-3a), 131.0 (C-6 Ar), 134.2 weak (C-5 Ar), 140.0 (C-1 Ar), 148.5 (C-NO_2), 152.0 (C-6), 154.4 (C-7a), 163.3 (C-3), 171.1 ($CONH_2$)
6.63 (br s, 2H, NH_2)	–	77.6 (C-5)
6.73 (d, ^{3}J = 7.5 Hz, 2H, H-2 H-6 Ph)	120.0 (2C, C-2 C-6 Ph)	120.0 (2C, C-2 C-6 Ph), 124.2 (C-4 Ph), 129.4 weak (2C, C-3 C-5 Ph), 150.5 (C-1 Ph)
7.03-7.07 (m, 1H, H-4 Ph)	124.2 (C-4 Ph)	120.0 (2C, C-2 C-6 Ph), 129.4 (2C, C-3 C-5 Ph)
7.28-7.32 (m, 2H, H-3 H-5 Ph)	129.4 (2C, C-3 C-5 Ph)	120.0 (2C, C-2 C-6 Ph), 124.2 (C-4 Ph), 129.4 (2C, C-3 C-5 Ph), 150.5 (C-1 Ph)
7.44-7.49 (m, 1H, Ar)	127.9 (C-4 Ar),	123.4 weak (C-3 Ar), 131.0 (C-6 Ar), 134.2 weak (C-5 Ar), 148.5 (C-NO_2)
7.58 (br s, $CONH_2$)	–	–
7.64-7.69 (m, 5H, Ph)	130.2 (CH Ph), 130.4 (CH Ph)	130.2 (CH Ph), 130.4 (CH Ph), 135.5 (C-1 Ph)
7.78-7.83 (m, 3H, Ar)	123.4 (C-3 Ar), 134.2 (C-5 Ar),	34.6 (C-4), 123.4 (C-3 Ar), 127.9 (C-4 Ar), 131.0 (C-6 Ar), 134.2 (C-5 Ar), 140.0 (C-1 Ar), 148.5 (C-NO_2)

^{1}H NMR (400 MHz, DMSO-d_6): 5.64 (s, 1H, H-4), 6.63 (br s, 2H, NH_2), 6.73 (d, ^{3}J = 7.5 Hz, 2H, H-2 H-6 Ph), 7.03-7.07 (m, 1H, H-4 Ph), 7.28-7.32 (m, 2H, H-3 H-5 Ph), 7.44-7.49 (m, 1H, H-4 Ar), 7.58 (br s, $CONH_2$), 7.64-7.69 (m, 5H, Ph), 7.78-7.83 (m, 3H, Ar).

^{13}C NMR (101 MHz, DMSO-d_6): 34.6 (C-4), 77.6 (C-5), 111.1 (C-3a), 120.0 (2C, C-2 C-6 Ph), 123.4 (C-3 Ar), 124.2 (C-4 Ph), 127.9 (C-4 Ar), 129.4 (2C, C-3 C-5 Ph), 130.2 (CH Ph), 130.4 (CH Ph), 131.0 (C-6 Ar), 134.2 (C-5 Ar), 135.5 (C-1 Ph), 140.0 (C-1 Ar), 148.5 (C-NO_2), 150.5 (C-1 Ph), 152.0 (C-6), 154.4 (C-7a), 163.3 (C-3), 171.1 ($CONH_2$).

Figure S6. ^{1}H NMR spectrum of compound 3c, DMSO-d_6 (400 MHz)

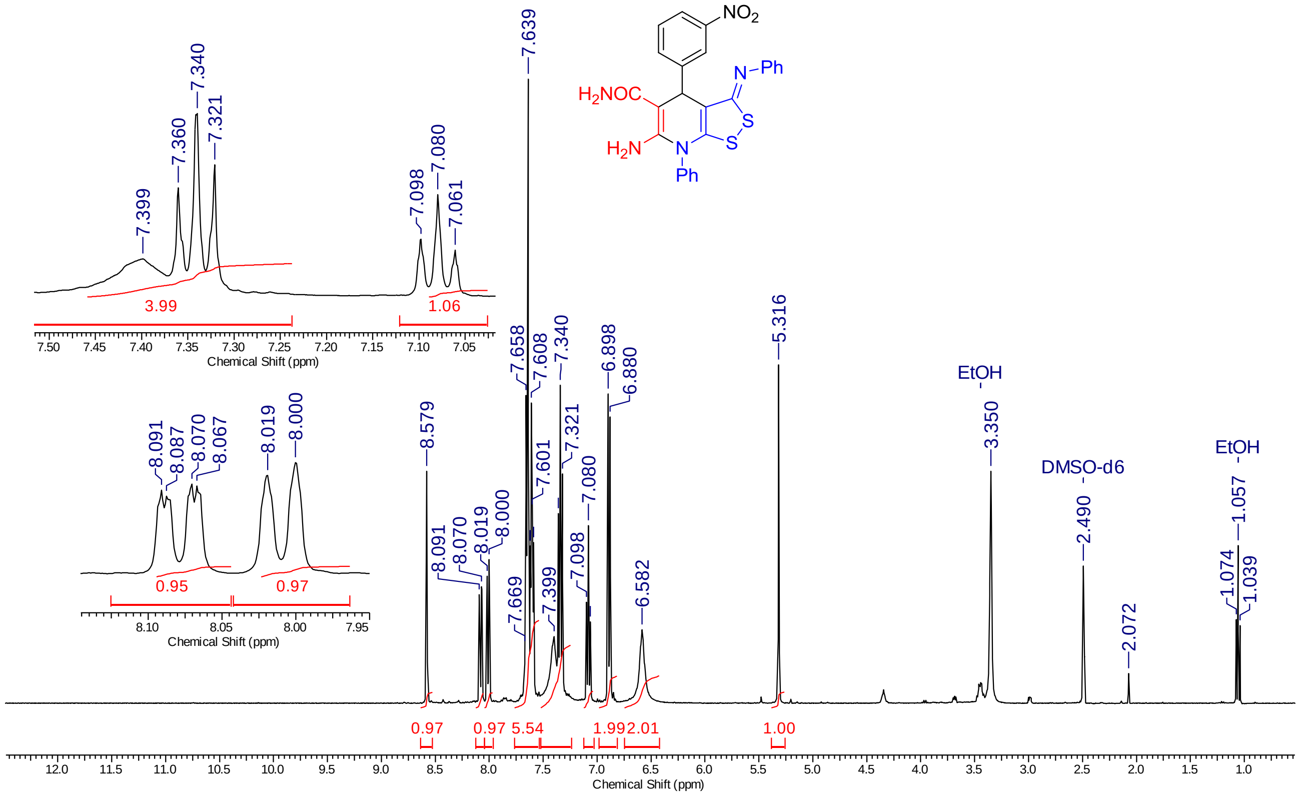

Figure S7. ^{13}C NMR spectrum of compound 3c, DMSO-d_6 (101 MHz)

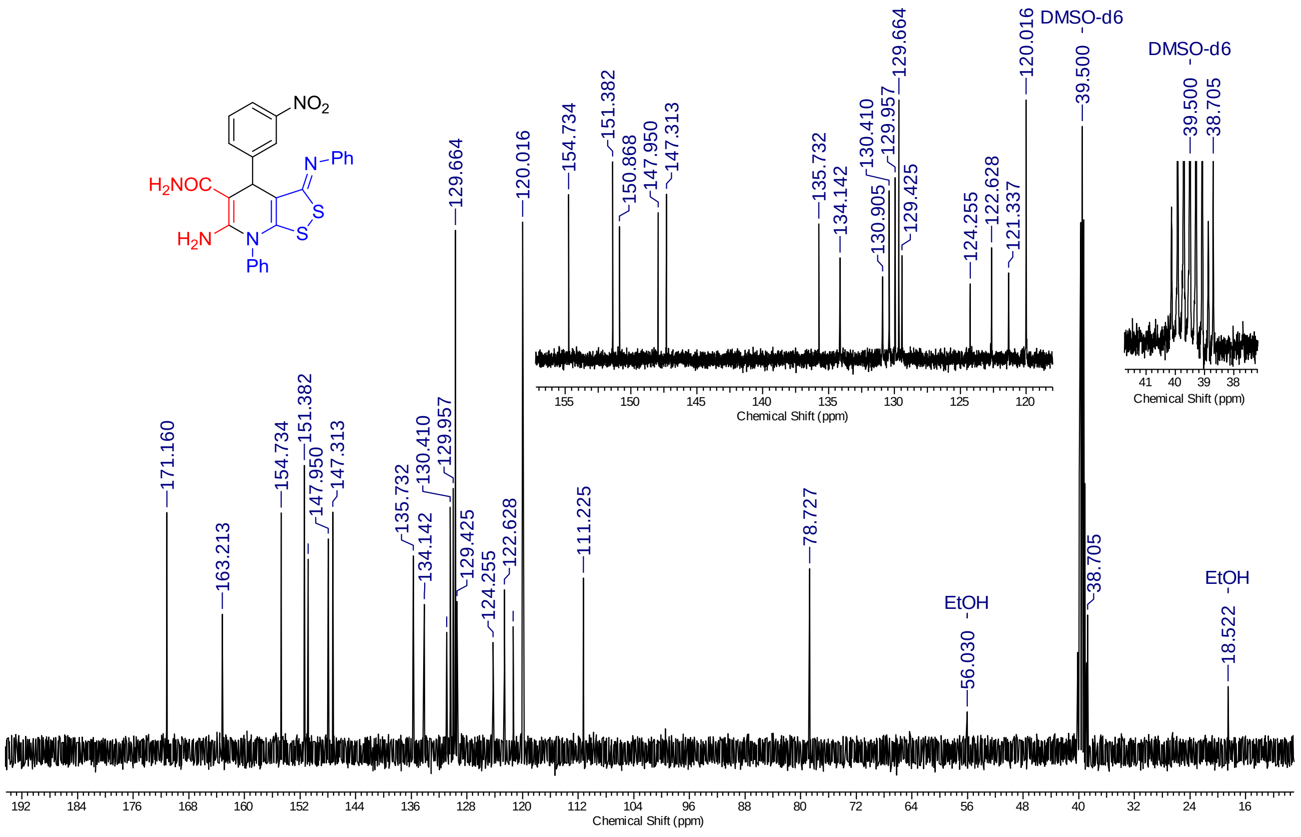

Figure S8. ^{1}H–^{13}C HSQC NMR spectrum of compound 3c, DMSO-d_6 (400/101 MHz)

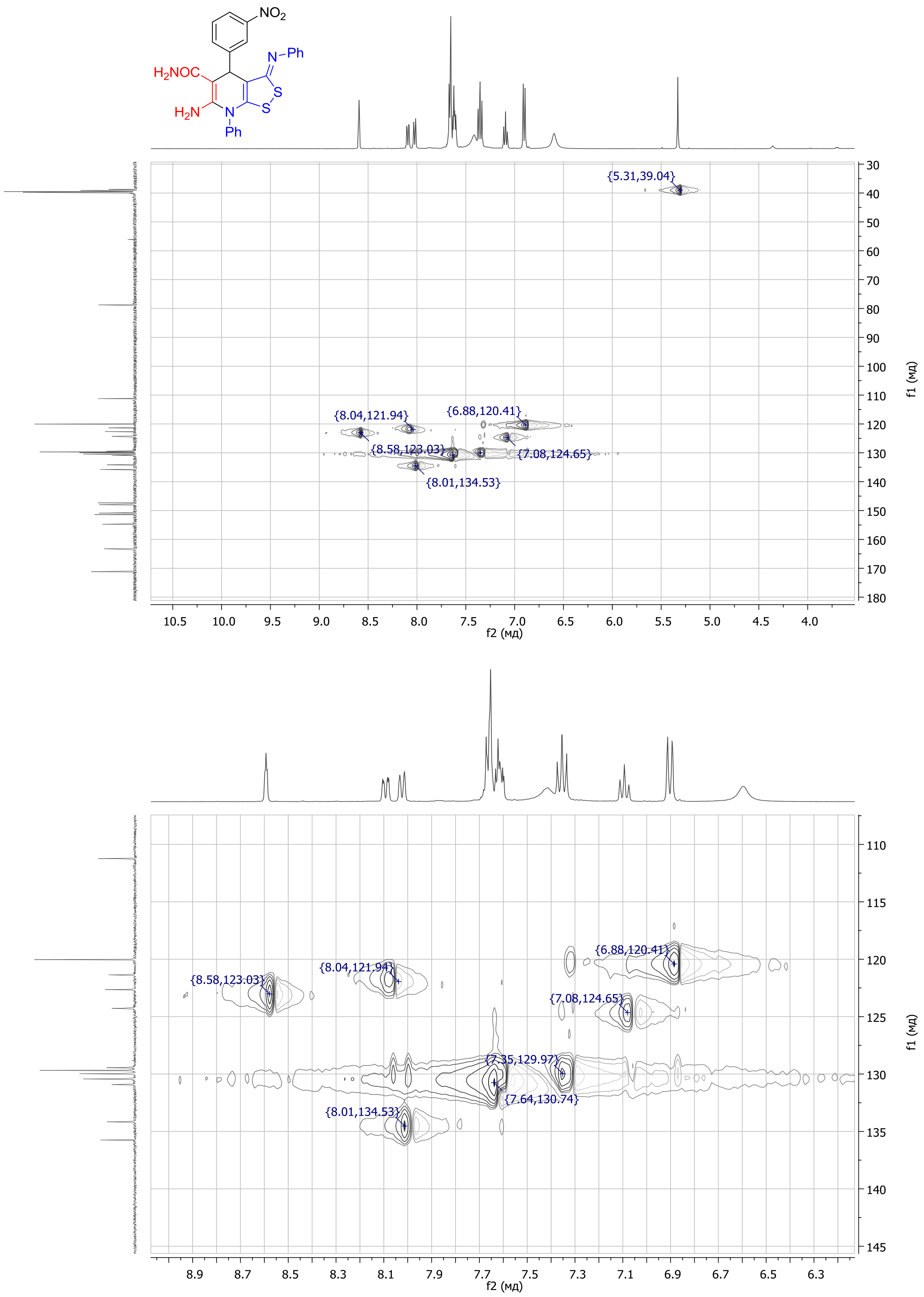

Table S2. The observed correlations in the ^{1}H-^{13}C HSQC 2D NMR spectrum of compound 3c

^{13}C chemical shifts are given in **red**, ^{1}H shifts – in **blue**

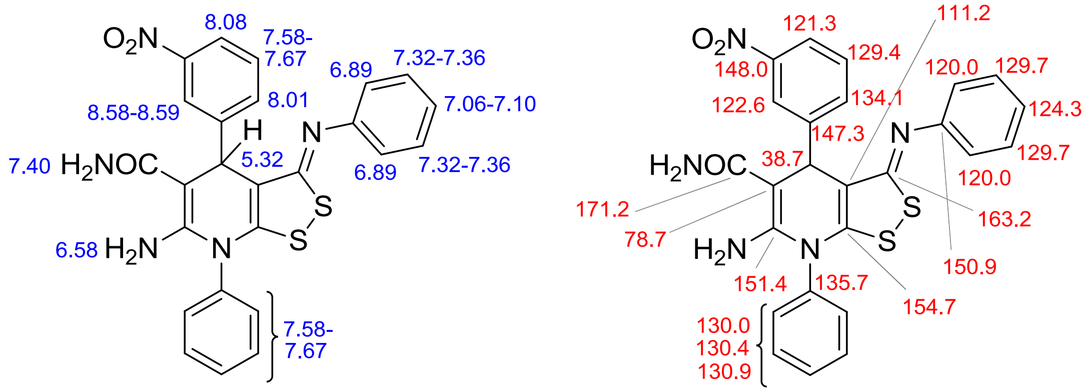

^{1}H NMR shifts, δ, ppm	Correlations in HSQC spectrum, δ, ppm
5.32 (s, 1H, H-4)	38.7 (C-4)
6.58 (br s, 2H, NH_2)	–
6.89 (d, ^{3}J = 7.5 Hz, 2H, H-2 H-6 Ph)	120.0 (2C, C-2 C-6 Ph)
7.06-7.10 (m, 1H, H-4 Ph)	124.3 (C-4 Ph)
7.32-7.36 (m, 2H, H-3 H-5 Ph)	129.7 (2C, C-3 C-5 Ph)
7.40 (br s, $CONH_2$)	–
7.58-7.67 (m, 6H, H-5 Ar + Ph)	129.4 (C-5 Ar), 130.0 (CH Ph), 130.4 (CH Ph), 130.9 (CH Ph)
8.01 (br d, ^{3}J = 7.5 Hz, H-6 Ar)	134.1 (C-6 Ar)
8.08 (dd, ^{3}J = 8.2 Hz, ^{3}J = 1.4 Hz, H-4 Ar)	121.3 (C-4 Ar)
8.58-8.59 (m, 1H, H-2 Ar)	122.6 (C-2 Ar)

^{1}H NMR (400 MHz, DMSO-d_6): 5.32 (s, 1H, H-4), 6.58 (br s, 2H, NH_2), 6.89 (d, ^{3}J = 7.5 Hz, 2H, H-2 H-6 Ph), 7.06-7.10 (m, 1H, H-4 Ph), 7.32-7.36 (m, 2H, H-3 H-5 Ph), 7.40 (br s, $CONH_2$), 7.58-7.67 (m, 6H, H-5 Ar + Ph), 8.01 (br d, ^{3}J = 7.5 Hz, H-6 Ar), 8.08 (dd, ^{3}J = 8.2 Hz, ^{3}J = 1.4 Hz, H-4 Ar), 8.58-8.59 (m, 1H, H-2 Ar).

^{13}C NMR (101 MHz, DMSO-d_6): 38.7 (C-4), 78.7 (C-5), 111.2 (C-3a), 120.0 (2C, C-2 C-6 Ph), 121.3 (C-4 Ar), 122.6 (C-2 Ar), 124.3 (C-4 Ph), 129.4 (C-5 Ar), 129.7 (2C, C-3 C-5 Ph), 130.0 (CH Ph), 130.4 (CH Ph), 130.9 (CH Ph), 134.1 (C-6 Ar), 135.7 (C-1 Ph), 147.3 (C-1 Ar), 148.0 (C-NO_2), 150.9 (C-1 Ph), 151.4 (C-6), 154.7 (C-7a), 163.2 (C-3), 171.2 ($CONH_2$).

Figure S9. FTIR spectrum of compound 3c

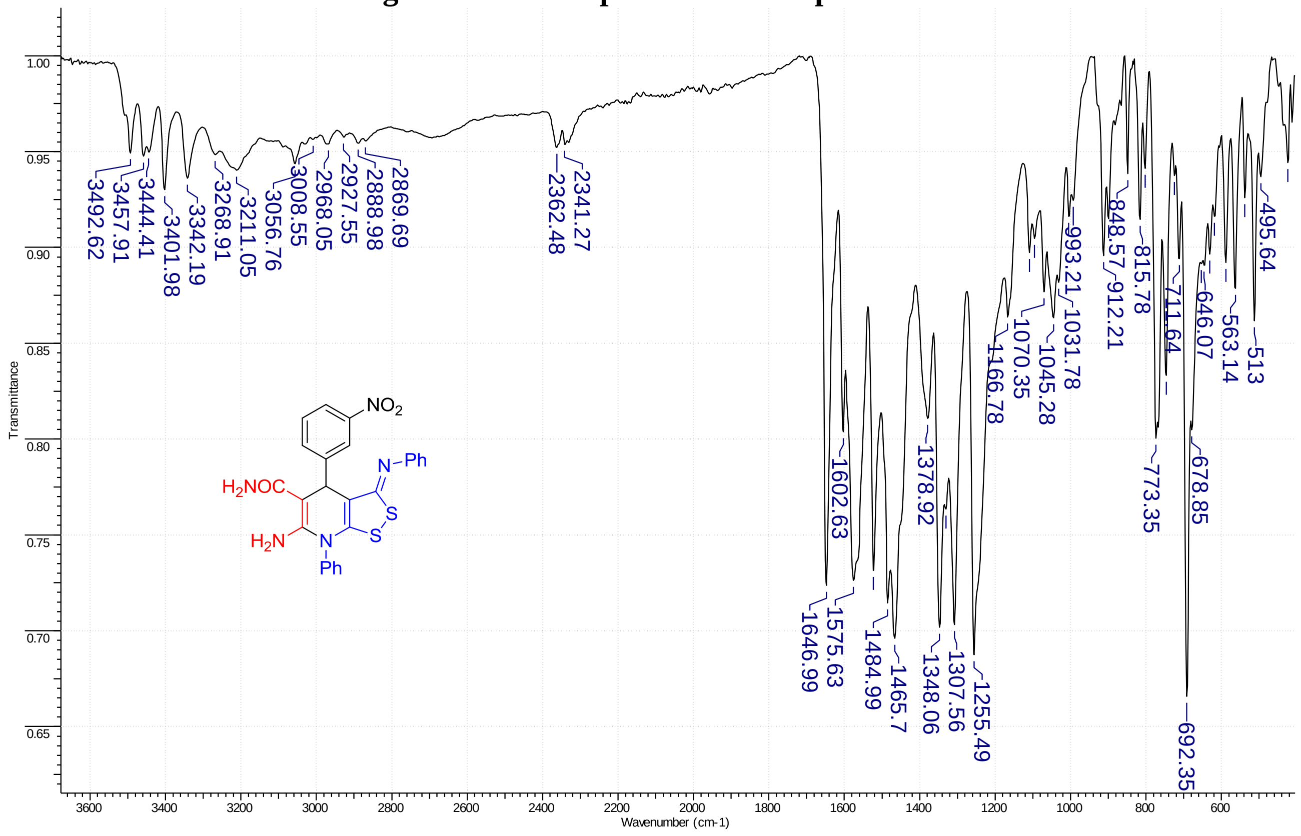

Figure S10. ^{1}H NMR spectrum of compound 3d (solvate with EtOH), DMSO-d_6 (400 MHz)

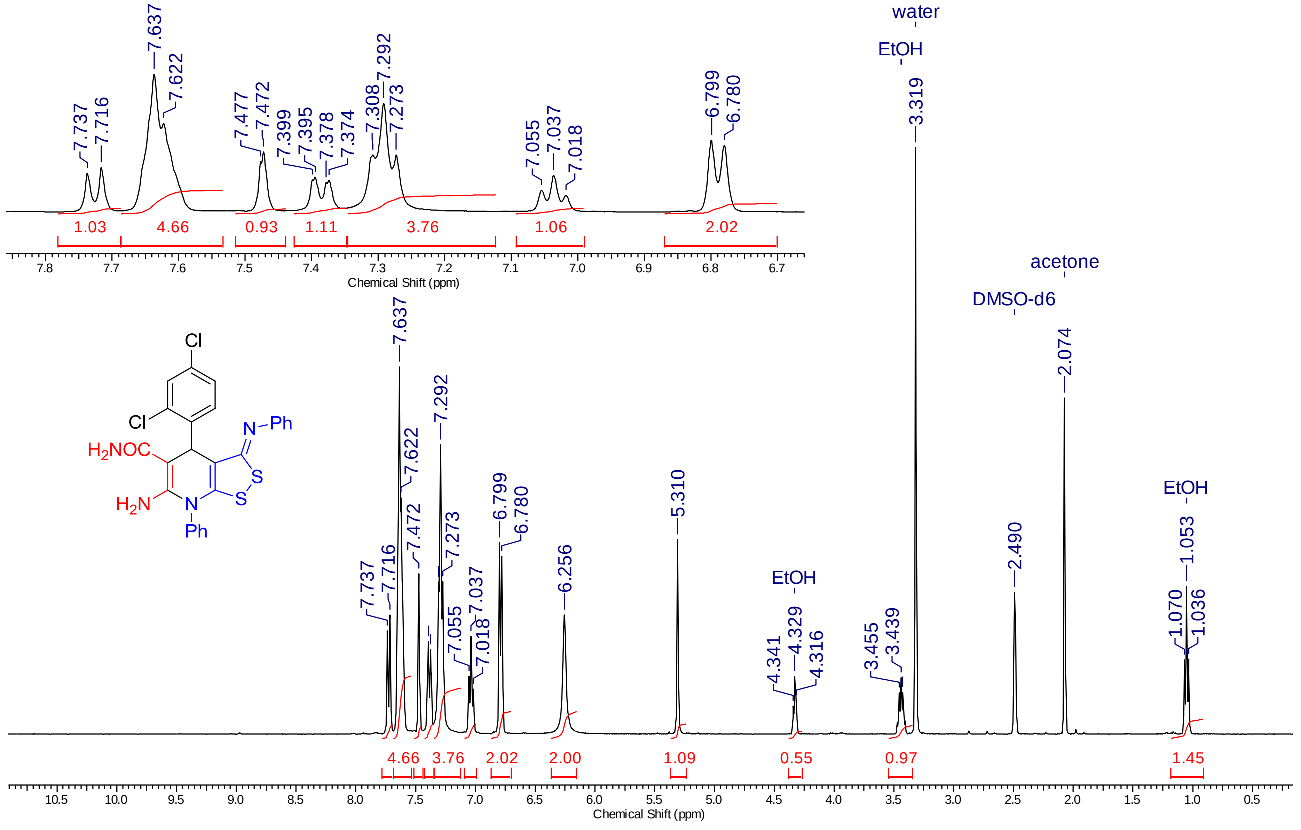

Figure S11. ^{13}C NMR spectrum of compound 3d (solvate with EtOH), DMSO-d_6 (101 MHz)

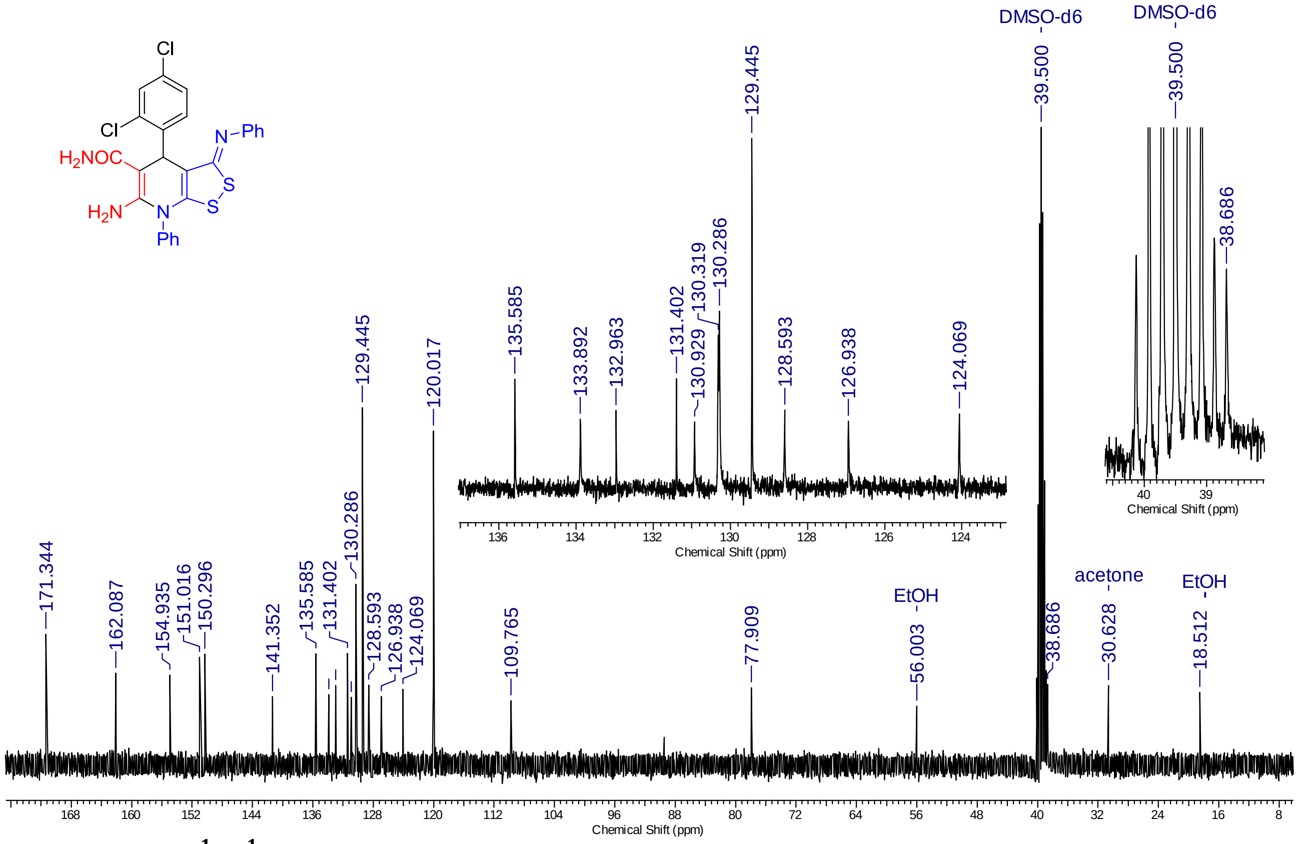

Figure S12. ^{1}H-^{1}H COSY NMR spectrum of compound 3d (solvate with EtOH), DMSO-d6 (400/400 MHz)

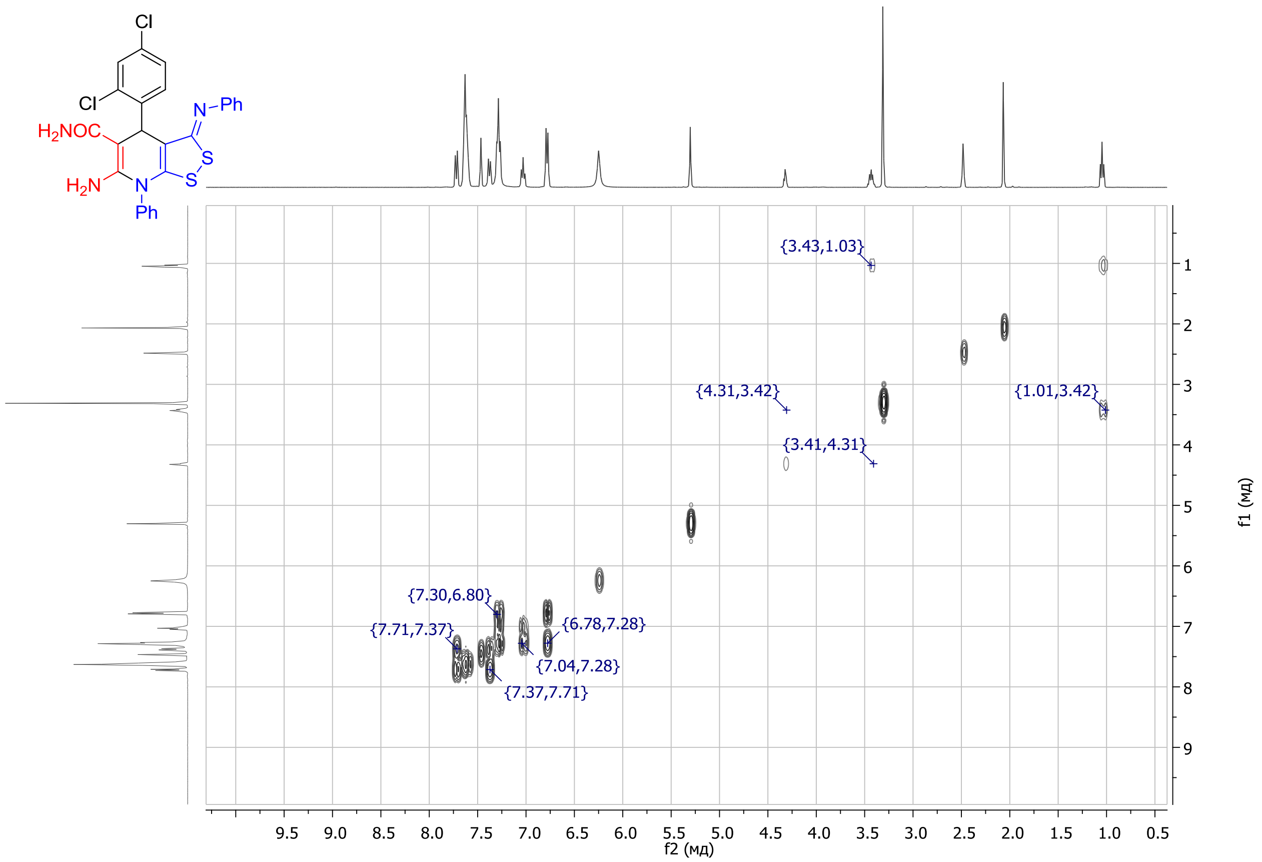

Figure S13. ^{1}H–^{13}C HSQC NMR spectrum of compound 3d (solvate with EtOH), DMSO-d_6 (100/400 MHz)

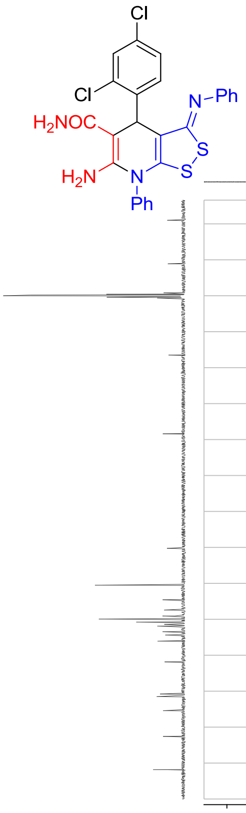

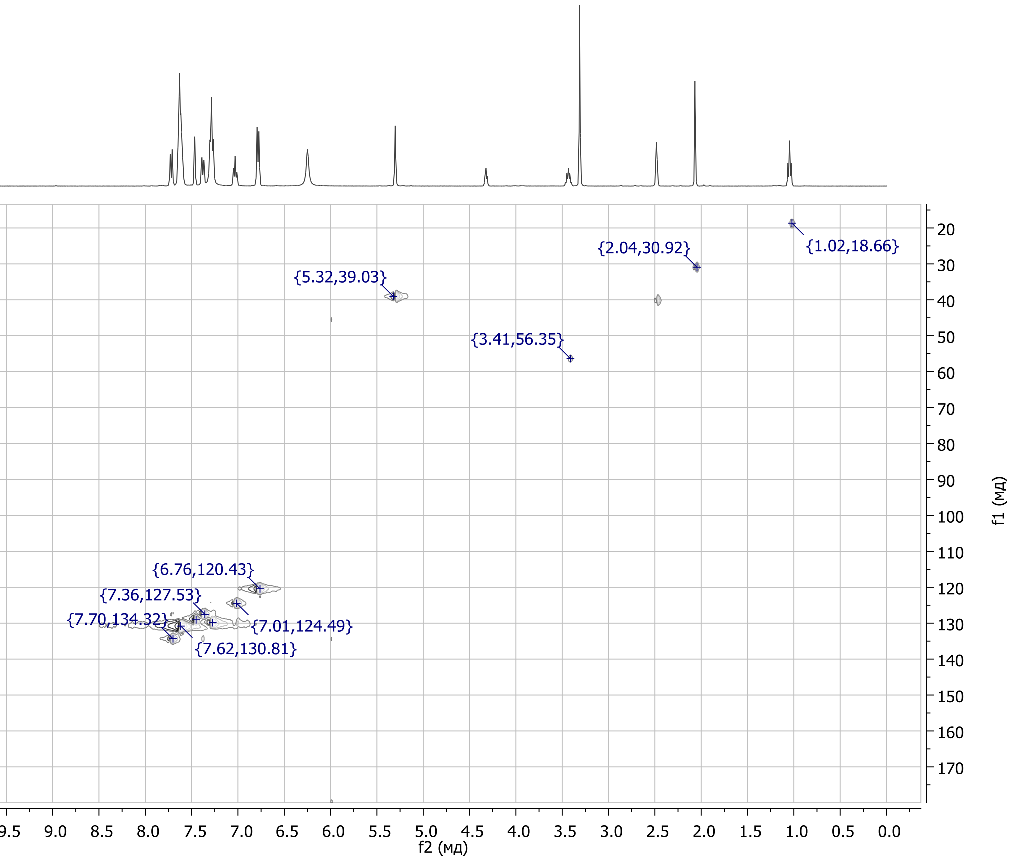

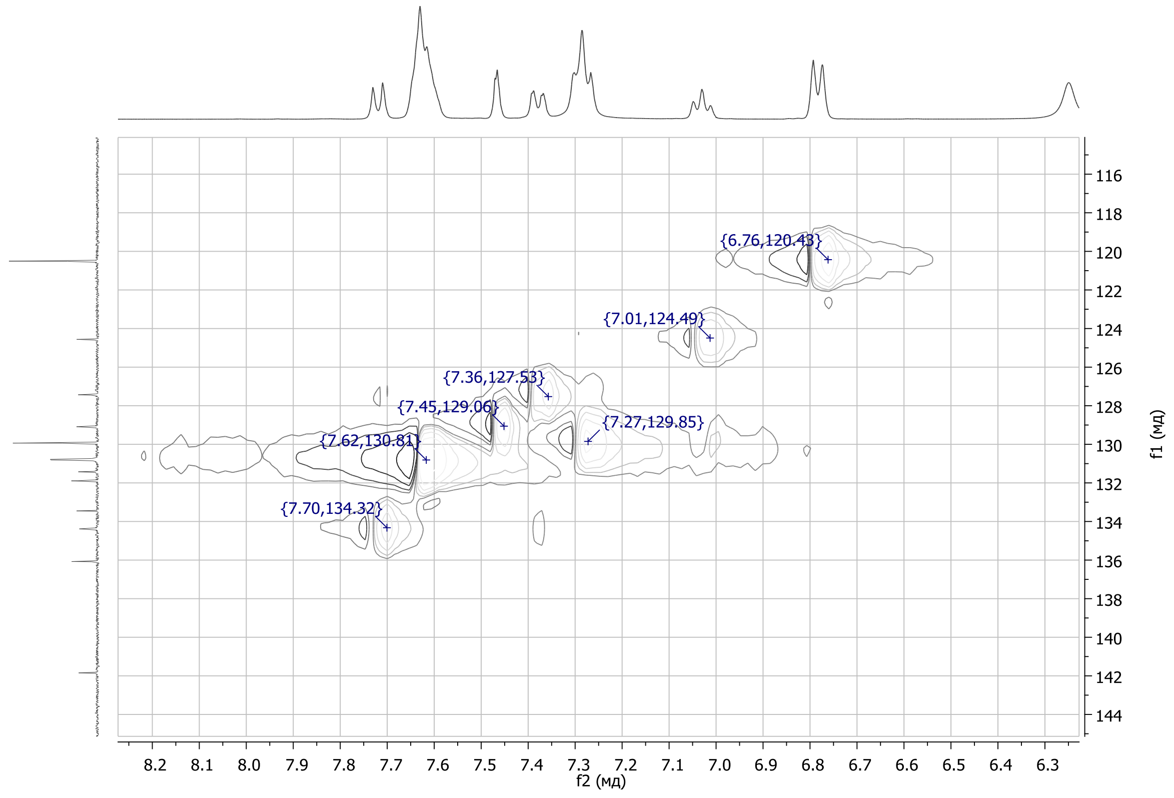

Figure S14. ^1H–^{13}C HMBC NMR spectrum of compound 3d (solvate with EtOH), DMSO-d_6 (100/400 MHz)

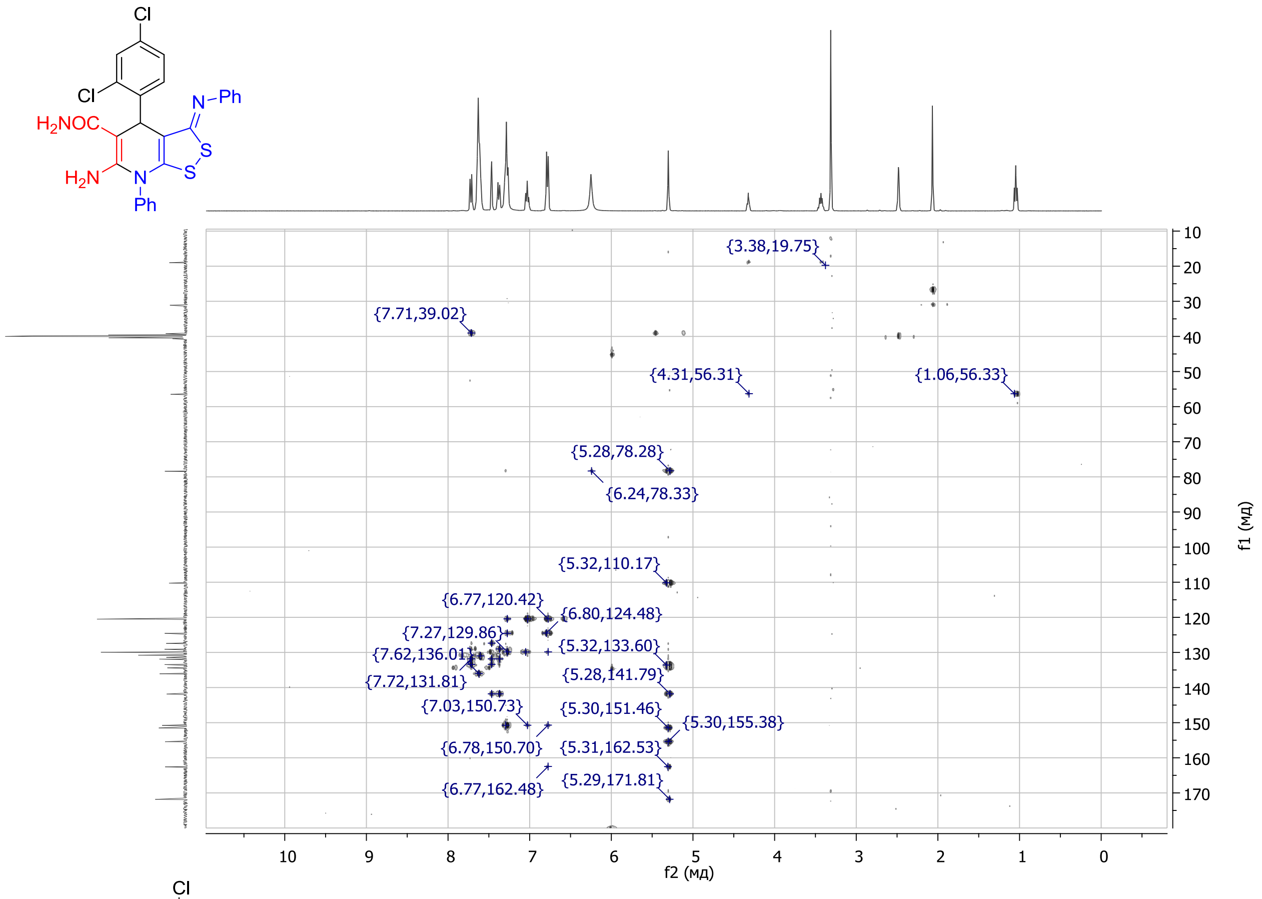

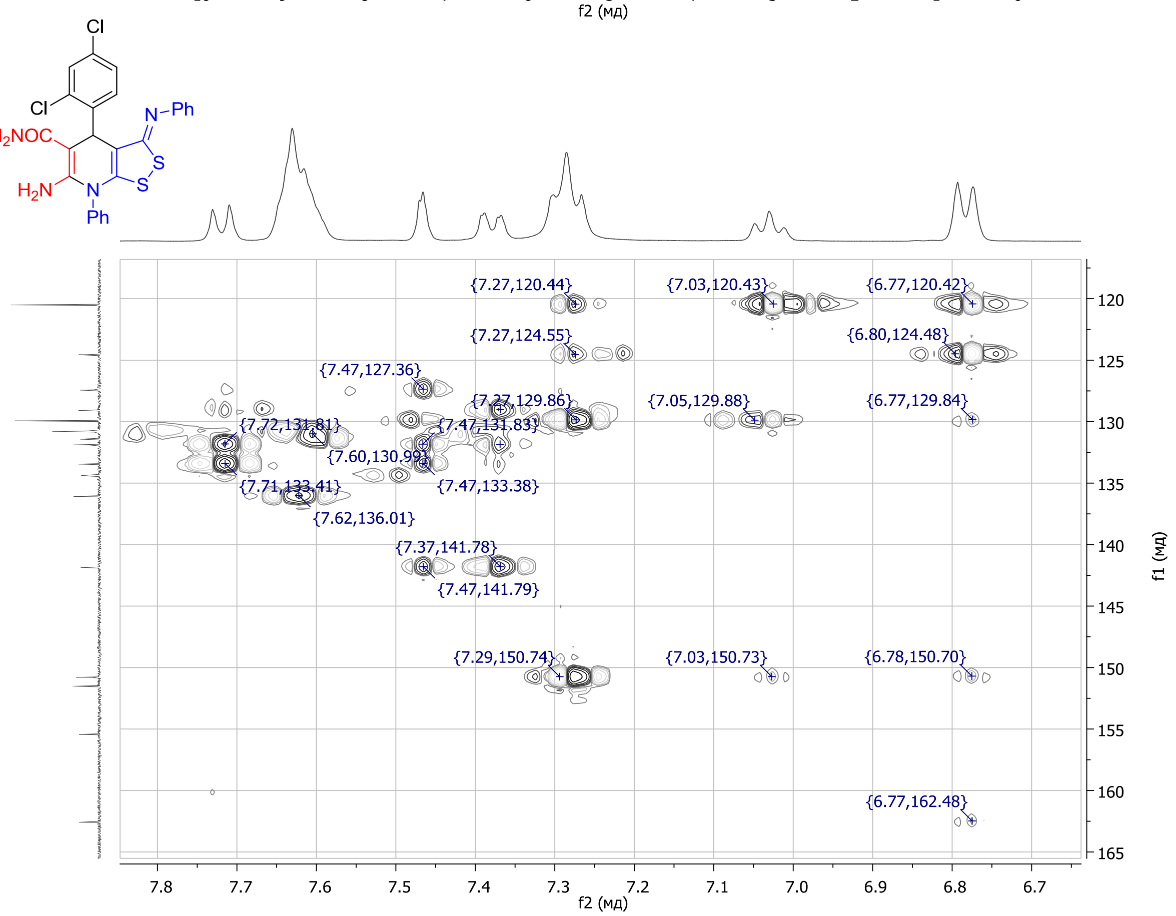

Table S3. The observed correlations in the ^{1}H-^{13}C HSQC and ^{1}H-^{13}C HMBC 2D NMR spectra of compound 3d (solvate with EtOH), DMSO-d_6 (100/400 MHz)

^{13}C chemical shifts are given in **red**, ^{1}H shifts – in **blue**

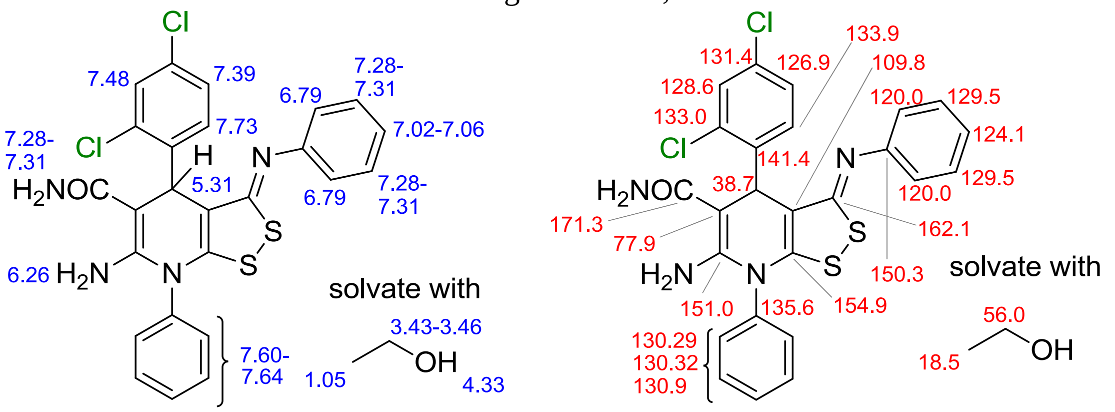

^{1}H NMR shifts, δ, ppm	Correlations in HSQC spectrum, δ, ppm	Correlations in HMBC spectrum, δ, ppm
1.05 (t, ^{3}J = 6.8 Hz, 3H, EtOH)	18.5 (CH_3 EtOH)	56.0 (CH_2 EtOH)
3.43-3.46 (m, 2H, EtOH)	56.0 (CH_2 EtOH)	18.5 (CH_3 EtOH)
4.33 (t, ^{3}J = 5.0 Hz, 1H, EtOH)	–	56.0 weak (CH_2 EtOH)
5.31 (s, 1H, H-4)	38.7 (C-4)	77.9 (C-5), 109.8 (C-3a), 133.0 (C–Cl Ar), 133.9 (C-6 Ar), 141.4 (C-1 Ar), 151.0 (C-6), 154.9 (C-7a), 162.1 (C-3), 171.3 ($CONH_2$)
6.26 (br s, 2H, NH_2)	–	77.9 weak (C-5)
6.79 (d, ^{3}J = 7.5 Hz, 2H, H-2 H-6 Ph)	120.0 (2C, C-2 C-6 Ph)	120.0 (2C, C-2 C-6 Ph), 124.1 (C-4 Ph), 129.5 weak (2C, C-3 C-5 Ph), 150.3 (C-1 Ph), 162.1 weak (C-3)
7.02-7.06 (m, 1H, H-4 Ph)	124.1 (C-4 Ph)	120.0 (2C, C-2 C-6 Ph), 129.5 (2C, C-3 C-5 Ph), 150.3 weak (C-1 Ph)
7.28-7.31 (m, 4H, H-3 H-5 Ph, $CONH_2$)	129.5 (2C, C-3 C-5 Ph)	120.0 (2C, C-2 C-6 Ph), 124.1 (C-4 Ph), 129.5 (2C, C-3 C-5 Ph), 150.3 (C-1 Ph)
7.39 (dd, ^{3}J = 8.2 Hz, ^{4}J = 1.7 Hz, H-5 Ar)	126.9 (C-5 Ar)	128.6 (C-3 Ar), 131.4 (C-4 Ar), 141.4 (C-1 Ar)
7.48 (d, ^{4}J = 1.7 Hz, H-3 Ar)	128.6 (C-3 Ar)	126.9 (C-5 Ar), 131.4 (C-4 Ar), 133.0 (C–Cl Ar), 141.4 (C-1 Ar)
7.60-7.64 (m, 5H, Ph)	130.29 (CH Ph), 130.32 (CH Ph), 130.9 (CH Ph)	130.29 (CH Ph), 130.32 (CH Ph), 130.9 (CH Ph), 135.6 (C-1 Ph)
7.73 (d, ^{3}J = 8.2 Hz, H-6 Ar)	133.9 (C-6 Ar)	38.7 (C-4), 131.4 (C-4 Ar), 133.0 (C2–Cl Ar)

^{1}H NMR (400 MHz, DMSO-d_6): 1.05 (t, ^{3}J = 6.8 Hz, 3H, EtOH), 3.43-3.46 (m, 2H, EtOH), 4.33 (t, ^{3}J = 5.0 Hz, 1H, EtOH), 5.31 (s, 1H, H-4), 6.26 (br s, 2H, NH_2), 6.79 (d, ^{3}J = 7.5 Hz, 2H, H-2 H-6 Ph), 7.02-7.06 (m, 1H, H-4 Ph), 7.28-7.31 (m, 4H, H-3 H-5 Ph, $CONH_2$), 7.39 (dd, ^{3}J = 8.2 Hz, ^{4}J = 1.7 Hz, H-5 Ar), 7.48 (d, ^{4}J = 1.7 Hz, H-3 Ar), 7.60-7.64 (m, 5H, Ph), 7.73 (d, ^{3}J = 8.2 Hz, H-6 Ar).

^{13}C NMR (101 MHz, DMSO-d_6): 18.5 (CH_3 EtOH), 38.7 (C-4), 56.0 (CH_2 EtOH), 77.9 (C-5), 109.8 (C-3a), 120.0 (2C, C-2 C-6 Ph), 124.1 (C-4 Ph), 126.9 (C-5 Ar), 128.6 (C-3 Ar), 129.5 (2C, C-3 C-5 Ph), 130.29 (CH Ph), 130.32 (CH Ph), 130.9 (CH Ph), 131.4 (C-4 Ar), 133.0 (C–Cl Ar), 133.9 (C-6 Ar), 135.6 (C-1 Ph), 141.4 (C-1 Ar), 150.3 (C-1 Ph), 151.0 (C-6), 154.9 (C-7a), 162.1 (C-3), 171.3 ($CONH_2$).

Figure S15. FTIR spectrum of compound 3d

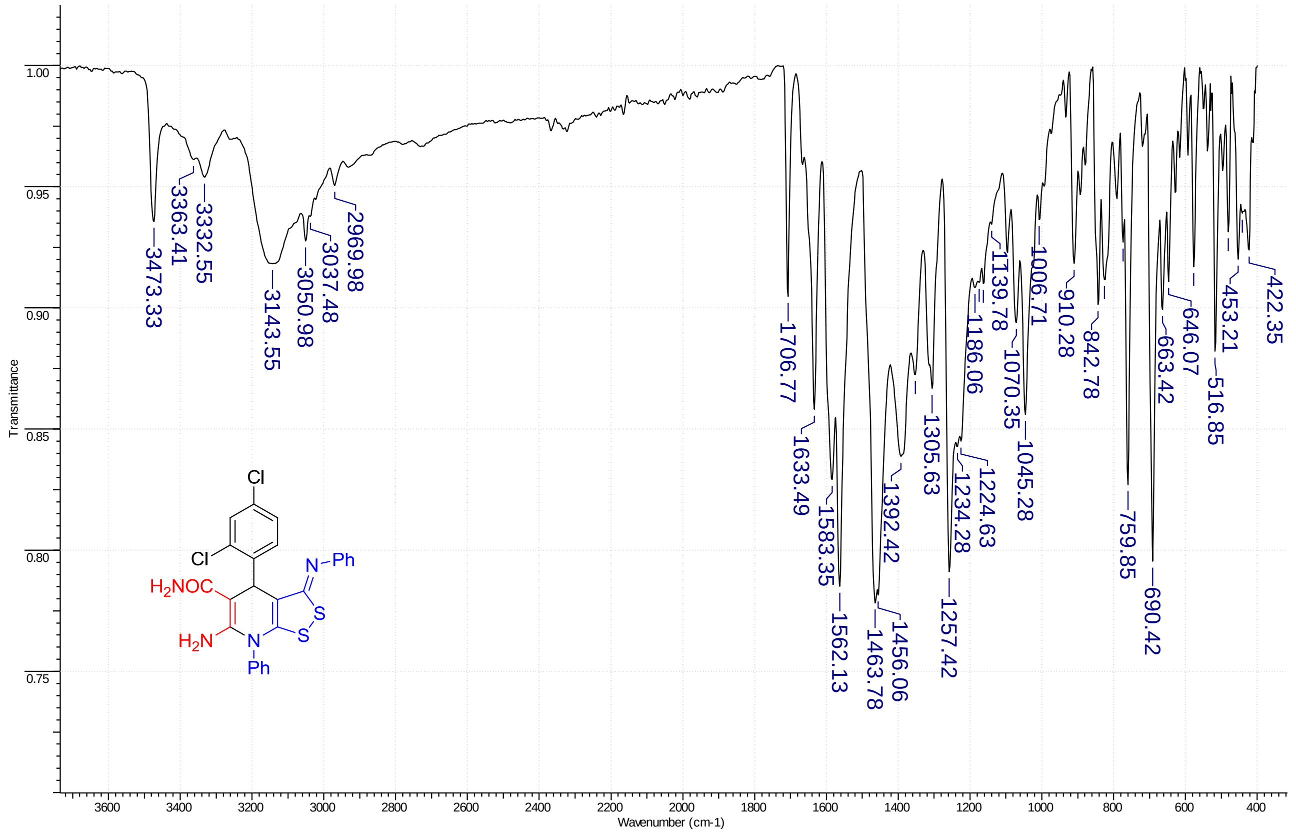

Figure S16. ORTEP drawings of the crystal structure showing 50% probability thermal ellipsoids (CCDC 2310349) and microphotography of the single crystal of compound 3d used for X-Ray diffraction analysis at the bottom.

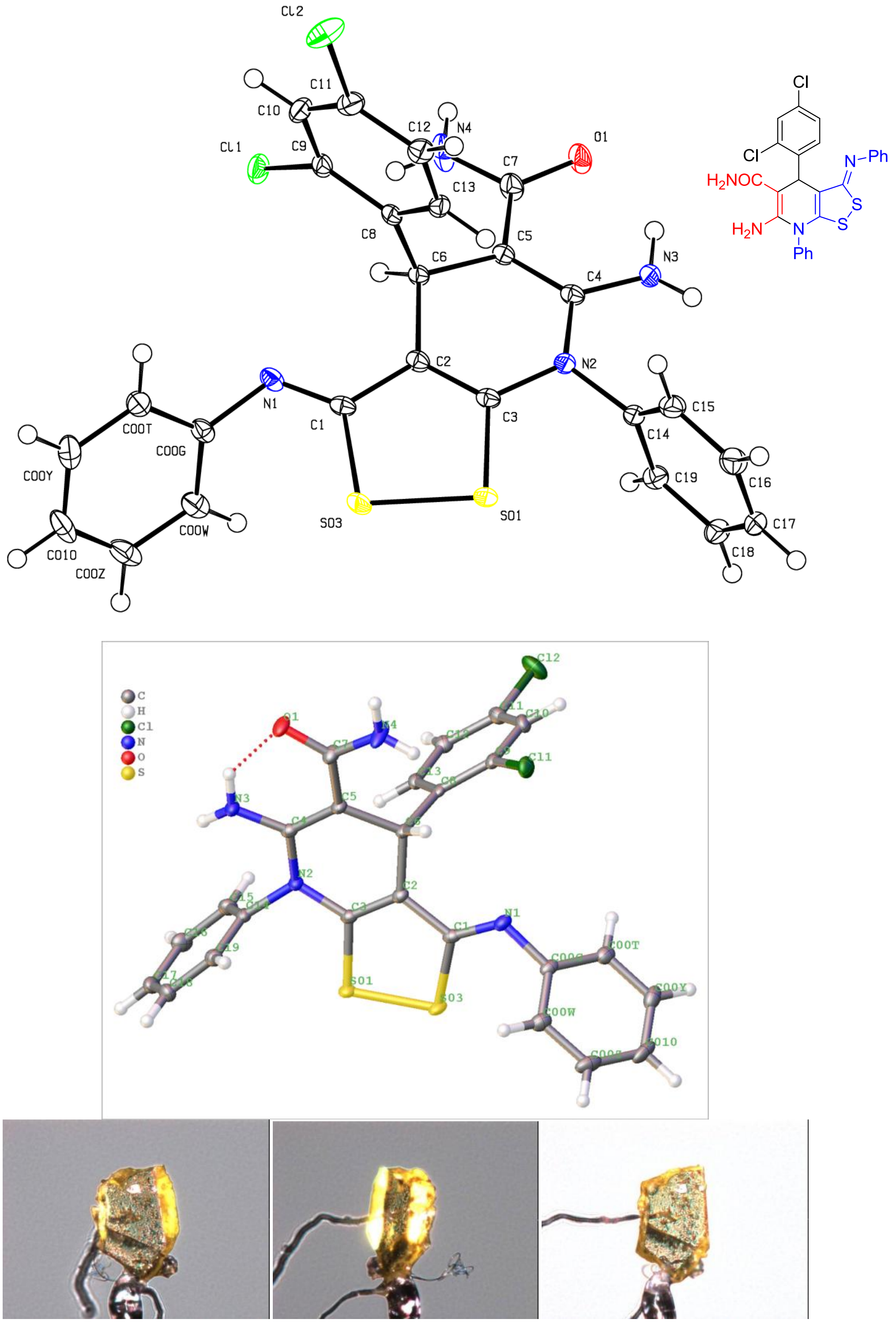

Table S4. Crystal data and structure refinement for 6-amino-4-(2,4-dichlorophenyl)-7-phenyl-3-(phenylimino)-4,7-dihydro-3H-[1,2]dithiolo[3,4-b]pyridine-5-carboxamide 3d

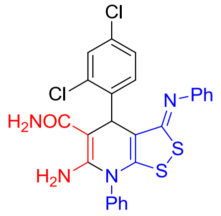

Identification code	ANNA_SAE106_2
Empirical formula	$C_{25}H_{18}Cl_2N_4OS_2$
Formula weight	525.45
Temperature/K	100.00(10)
Crystal system	monoclinic
Space group	I2/a
a/Å	11.97350(10)
b/Å	12.96920(10)
c/Å	34.4739(2)
α/°	90
β/°	90.7890(10)
γ/°	90
Volume/Å^3	5352.83(7)
Z	8
ρ_{calc}g/cm^3	1.304
μ/mm^{-1}	3.837
F(000)	2160.0
Crystal size/mm^3	0.507 × 0.386 × 0.167
Radiation	Cu Kα (λ = 1.54184)
2Θ range for data collection/	7.282 to 153.23
Index ranges	$-14 \leq h \leq 12$, $-15 \leq k \leq 16$, $-43 \leq l \leq 43$
Reflections collected	29542
Independent reflections	5572 [R_{int} = 0.0263, R_{sigma} = 0.0158]
Data/restraints/parameters	5572/0/323
Goodness-of-fit on F^2	1.048
Final R indexes [I>=2σ (I)]	R_1 = 0.0352, wR_2 = 0.0936
Final R indexes [all data]	R_1 = 0.0356, wR_2 = 0.0939
Largest diff. peak/hole / e Å^{-3}	0.34/-0.44

Table S5. Fractional Atomic Coordinates (×10^4) and Equivalent Isotropic Displacement Parameters (Å^2×10^3) for compound 3d. U_{eq} is defined as 1/3 of of the trace of the orthogonalised U_{IJ} tensor.

Atom	*x*	*y*	*z*	U(eq)
S01	2860.6(3)	6914.6(3)	2896.5(2)	16.39(10)
Cl1	7647.4(3)	5958.7(3)	3929.7(2)	20.73(10)
S03	4330.3(3)	6571.3(3)	2614.7(2)	18.15(10)
Cl2	7933.1(4)	10013.6(4)	4195.1(2)	30.45(12)
O1	3800.9(10)	5693.2(11)	4756.5(3)	22.3(3)
N2	2753.5(11)	6928.8(11)	3668.3(4)	13.9(3)
N1	6138.5(12)	5934.7(11)	3035.9(4)	15.9(3)
N3	2257.4(12)	6541.5(12)	4300.6(4)	17.0(3)
N4	5472.7(15)	5294.0(15)	4512.5(5)	30.8(4)
C2	4518.0(14)	6435.5(12)	3396.1(4)	13.7(3)
C8	5811.3(13)	7221.7(13)	3885.2(4)	13.4(3)
C14	1597.5(13)	7169.5(13)	3571.9(4)	14.2(3)
C5	4145.5(13)	6175.7(13)	4097.7(4)	13.7(3)
C00G	6642.1(14)	5730.9(13)	2673.0(5)	15.8(3)
C13	5345.5(13)	8208.5(13)	3915.2(5)	15.0(3)
C1	5131.1(14)	6255.5(12)	3045.1(4)	14.2(3)
C3	3446.9(14)	6749.8(12)	3358.4(4)	13.5(3)
C4	3073.4(14)	6542.9(12)	4036.0(4)	13.6(3)
C19	896.3(14)	6383.5(13)	3444.7(5)	16.5(3)
C15	1248.0(15)	8186.3(13)	3577.8(5)	18.2(3)
C12	5971.2(14)	9073.9(13)	4009.6(5)	17.6(3)
C6	5047.4(13)	6302.6(12)	3792.7(4)	12.8(3)
C7	4449.1(15)	5711.0(14)	4470.7(5)	18.4(3)
C10	7626.1(14)	7996.2(14)	4040.5(5)	19.5(3)
C11	7114.5(15)	8945.6(14)	4071.2(5)	19.5(3)
C18	-178.4(14)	6629.7(14)	3318.1(5)	19.6(3)
C00T	7617.5(14)	6253.4(14)	2579.3(5)	18.8(3)
C9	6963.7(14)	7143.2(13)	3949.6(4)	15.9(3)
C17	-535.8(14)	7650.0(15)	3319.5(5)	20.2(3)
C00W	6215.0(16)	4986.8(13)	2418.2(5)	20.2(3)
C16	173.7(15)	8426.2(14)	3448.9(5)	21.6(4)
C00Y	8138.0(16)	6070.9(15)	2227.1(5)	24.3(4)
C00Z	6750.2(17)	4801.2(15)	2067.4(5)	25.2(4)
C010	7702.3(17)	5348.7(16)	1972.1(5)	26.7(4)

Table S6. Anisotropic Displacement Parameters (Å^2×10^3) for 3d. The Anisotropic displacement factor exponent takes the form: $-2\pi^2[h^2a^{*2}U_{11}+...+2hka^*b^*U_{12}+ …]$

Atom	U_{11}	U_{22}	U_{33}	U_{23}	U_{13}	U_{12}
S01	15.89(19)	24.4(2)	8.86(17)	1.07(14)	-1.83(14)	0.95(15)
Cl1	14.25(19)	25.2(2)	22.8(2)	2.33(15)	2.20(15)	6.88(15)
S03	19.8(2)	26.6(2)	8.06(17)	0.94(14)	0.44(14)	3.14(16)
Cl2	22.7(2)	27.4(2)	40.9(3)	3.31(19)	-12.88(19)	-7.62(17)
O1	20.3(6)	33.8(7)	12.8(5)	8.8(5)	5.4(5)	6.7(5)
N2	12.5(6)	19.4(7)	9.9(6)	1.8(5)	-0.1(5)	0.0(5)
N1	19.7(7)	17.4(7)	10.5(6)	0.9(5)	2.2(5)	1.5(5)
N3	13.2(7)	26.9(8)	10.8(7)	2.1(5)	1.1(5)	0.1(6)
N4	26.0(8)	50.1(11)	16.6(7)	18.3(7)	9.9(6)	19.8(8)
C2	16.5(8)	14.2(7)	10.5(7)	1.4(6)	1.0(6)	-1.2(6)
C8	13.9(7)	19.1(8)	7.1(6)	2.8(6)	2.0(5)	0.8(6)
C14	12.7(7)	20.0(8)	9.9(7)	1.5(6)	0.0(5)	0.4(6)
C5	14.8(7)	17.0(7)	9.2(7)	2.3(6)	0.5(6)	0.3(6)
C00G	19.9(8)	16.7(8)	10.7(7)	2.0(6)	0.9(6)	6.3(6)
C13	12.1(7)	20.7(8)	12.2(7)	3.1(6)	-0.1(6)	1.3(6)
C1	19.3(8)	13.4(7)	9.7(7)	1.2(6)	-1.0(6)	-2.0(6)
C3	16.3(8)	14.0(7)	10.0(7)	0.6(5)	-0.6(6)	-2.0(6)
C4	16.8(8)	13.9(7)	10.0(7)	0.8(6)	0.1(6)	-2.0(6)
C19	17.4(8)	16.8(8)	15.2(7)	2.1(6)	-0.4(6)	-1.3(6)
C15	19.3(8)	18.0(8)	17.2(8)	-3.2(6)	-1.8(6)	-0.9(6)
C12	18.8(8)	18.7(8)	15.4(7)	3.7(6)	-0.5(6)	0.5(6)
C6	13.2(7)	16.2(7)	9.0(7)	2.1(5)	0.2(5)	2.0(6)
C7	19.3(8)	22.0(8)	13.8(7)	5.7(6)	3.4(6)	3.2(7)
C10	11.1(7)	29.8(9)	17.5(8)	3.5(7)	-1.1(6)	0.5(7)
C11	17.7(8)	23.9(9)	16.7(8)	2.5(6)	-2.0(6)	-4.8(7)
C18	15.5(8)	24.5(9)	18.8(8)	-0.5(7)	-0.2(6)	-3.9(7)
C00T	19.1(8)	21.5(8)	15.8(8)	1.1(6)	-0.4(6)	6.1(7)
C9	14.1(8)	23.2(8)	10.4(7)	3.1(6)	2.0(6)	3.6(6)
C17	13.5(8)	30.7(9)	16.3(8)	-1.7(7)	-1.5(6)	5.0(7)
C00W	25.6(9)	18.9(8)	16.2(8)	-1.0(6)	0.3(7)	3.4(7)
C16	23.2(9)	20.7(8)	21.0(8)	-3.1(7)	-1.6(7)	6.6(7)
C00Y	20.8(9)	30.1(10)	22.2(9)	7.3(7)	7.1(7)	8.5(7)
C00Z	37.5(11)	23.3(9)	14.7(8)	-4.0(7)	-2.3(7)	12.9(8)
C010	34.3(10)	32.6(10)	13.3(8)	2.6(7)	7.5(7)	17.9(8)

Table S7. Bond Lengths for compound 3d.

Atom	Atom	Length/Å	Atom	Atom	Length/Å
S01	S03	2.0701(6)	C14	C19	1.388(2)
S01	C3	1.7447(16)	C14	C15	1.384(2)
Cl1	C9	1.7425(17)	C5	C4	1.383(2)
S03	C1	1.8024(16)	C5	C6	1.527(2)
Cl2	C11	1.7465(18)	C5	C7	1.462(2)
O1	C7	1.263(2)	C00G	C00T	1.392(3)
N2	C14	1.453(2)	C00G	C00W	1.397(2)
N2	C3	1.382(2)	C13	C12	1.386(2)
N2	C4	1.411(2)	C19	C18	1.390(2)
N1	C00G	1.421(2)	C15	C16	1.390(2)
N1	C1	1.277(2)	C12	C11	1.392(2)
N3	C4	1.346(2)	C10	C11	1.380(3)
N4	C7	1.346(2)	C10	C9	1.394(3)
C2	C1	1.443(2)	C18	C17	1.391(3)
C2	C3	1.350(2)	C00T	C00Y	1.393(2)
C2	C6	1.509(2)	C17	C16	1.387(3)
C8	C13	1.400(2)	C00W	C00Z	1.397(2)
C8	C6	1.533(2)	C00Y	C010	1.382(3)
C8	C9	1.398(2)	C00Z	C010	1.386(3)

Table S8. Bond Angles for compound 3d.

Atom	Atom	Atom	Angle/˚	Atom	Atom	Atom	Angle/˚
C3	S01	S03	93.89(6)	N3	C4	N2	114.75(14)
C1	S03	S01	96.24(6)	N3	C4	C5	125.19(15)
C3	N2	C14	116.10(13)	C5	C4	N2	120.03(14)
C3	N2	C4	118.45(14)	C14	C19	C18	118.93(16)
C4	N2	C14	121.86(13)	C14	C15	C16	119.16(16)
C1	N1	C00G	119.66(14)	C13	C12	C11	117.79(16)
C1	C2	C6	121.95(14)	C2	C6	C8	109.98(13)
C3	C2	C1	117.48(14)	C2	C6	C5	110.12(13)
C3	C2	C6	120.56(14)	C5	C6	C8	111.47(13)
C13	C8	C6	119.26(14)	O1	C7	N4	118.54(15)
C9	C8	C13	116.61(15)	O1	C7	C5	123.08(15)
C9	C8	C6	124.12(15)	N4	C7	C5	118.37(15)
C19	C14	N2	118.99(15)	C11	C10	C9	118.26(15)
C15	C14	N2	119.26(15)	C12	C11	Cl2	119.33(14)
C15	C14	C19	121.52(15)	C10	C11	Cl2	118.57(13)
C4	C5	C6	121.39(14)	C10	C11	C12	122.10(16)
C4	C5	C7	119.77(14)	C19	C18	C17	120.06(16)
C7	C5	C6	118.74(14)	C00G	C00T	C00Y	120.44(17)
C00T	C00G	N1	118.77(15)	C8	C9	Cl1	121.40(13)
C00T	C00G	C00W	119.43(15)	C10	C9	Cl1	116.25(13)
C00W	C00G	N1	121.72(16)	C10	C9	C8	122.34(16)
C12	C13	C8	122.89(15)	C16	C17	C18	120.31(16)
N1	C1	S03	123.07(12)	C00G	C00W	C00Z	119.66(18)
N1	C1	C2	124.41(15)	C17	C16	C15	120.02(16)
C2	C1	S03	112.49(12)	C010	C00Y	C00T	120.02(18)
N2	C3	S01	116.53(12)	C010	C00Z	C00W	120.35(18)
C2	C3	S01	119.64(12)	C00Y	C010	C00Z	120.05(16)
C2	C3	N2	123.80(14)				

Table S9. Torsion Angles for compound 3d

A	B	C	D	Angle/˚	A	B	C	D	Angle/˚
S01	S03	C1	N1	177.01(14)	C4	N2	C14	C19	-84.06(19)
S01	S03	C1	C2	-4.70(12)	C4	N2	C14	C15	101.32(18)
S03	S01	C3	N2	178.51(12)	C4	N2	C3	S01	162.83(12)
S03	S01	C3	C2	-3.21(14)	C4	N2	C3	C2	-15.4(2)
N2	C14	C19	C18	-174.05(14)	C4	C5	C6	C2	-23.4(2)
N2	C14	C15	C16	173.72(15)	C4	C5	C6	C8	98.94(17)
N1	C00G	C00T	C00Y	179.72(15)	C4	C5	C7	O1	-5.6(3)
N1	C00G	C00W	C00Z	-178.90(15)	C4	C5	C7	N4	175.28(18)
C8	C13	C12	C11	0.9(2)	C19	C14	C15	C16	-0.8(3)
C14	N2	C3	S01	3.78(19)	C19	C18	C17	C16	-0.3(3)
C14	N2	C3	C2	-174.42(15)	C15	C14	C19	C18	0.5(2)
C14	N2	C4	N3	-8.0(2)	C6	C2	C1	S03	-175.32(12)
C14	N2	C4	C5	170.10(15)	C6	C2	C1	N1	2.9(3)
C14	C19	C18	C17	0.1(2)	C6	C2	C3	S01	179.12(12)
C14	C15	C16	C17	0.5(3)	C6	C2	C3	N2	-2.7(2)
C00G	N1	C1	S03	-5.3(2)	C6	C8	C13	C12	177.96(14)
C00G	N1	C1	C2	176.63(15)	C6	C8	C9	Cl1	0.0(2)
C00G	C00T	C00Y	C010	-1.6(3)	C6	C8	C9	C10	-178.74(15)
C00G	C00W	C00Z	C010	0.2(3)	C6	C5	C4	N2	8.0(2)
C13	C8	C6	C2	63.39(18)	C6	C5	C4	N3	-174.09(15)
C13	C8	C6	C5	-59.05(18)	C6	C5	C7	O1	170.78(16)
C13	C8	C9	Cl1	178.88(11)	C6	C5	C7	N4	-8.4(3)
C13	C8	C9	C10	0.2(2)	C7	C5	C4	N2	-175.74(15)
C13	C12	C11	Cl2	-179.42(12)	C7	C5	C4	N3	2.1(3)
C13	C12	C11	C10	0.1(3)	C7	C5	C6	C2	160.31(15)
C1	N1	C00G	C00T	122.10(18)	C7	C5	C6	C8	-77.33(18)
C1	N1	C00G	C00W	-61.1(2)	C11	C10	C9	Cl1	-178.04(13)
C1	C2	C3	S01	0.3(2)	C11	C10	C9	C8	0.7(2)
C1	C2	C3	N2	178.48(14)	C18	C17	C16	C15	0.0(3)
C1	C2	C6	C8	76.26(18)	C00T	C00G	C00W	C00Z	-2.1(3)
C1	C2	C6	C5	-160.51(14)	C00T	C00Y	C010	C00Z	-0.3(3)
C3	N2	C14	C19	74.22(19)	C9	C8	C13	C12	-1.0(2)
C3	N2	C14	C15	-100.40(18)	C9	C8	C6	C2	-117.72(16)
C3	N2	C4	N3	-165.78(14)	C9	C8	C6	C5	119.84(16)
C3	N2	C4	C5	12.3(2)	C9	C10	C11	Cl2	178.65(12)
C3	C2	C1	S03	3.44(19)	C9	C10	C11	C12	-0.9(3)
C3	C2	C1	N1	-178.30(16)	C00W	C00G	C00T	C00Y	2.8(2)
C3	C2	C6	C8	-102.46(17)	C00W	C00Z	C010	C00Y	1.0(3)
C3	C2	C6	C5	20.8(2)					

Table S10. Hydrogen Atom Coordinates (Å×10^4) and Isotropic Displacement Parameters (Å^2×10^3) for compound 3d.

Atom	*x*	*y*	*z*	U(eq)
H13	4582.75	8286.41	3869.86	18
H19	1141.35	5703.06	3444.09	20
H15	1725.6	8702.67	3666.81	22
H12	5638.57	9718.98	4031.07	21
H6	5503.94	5675.53	3790.55	15
H10	8392.6	7927.14	4079.31	23
H18	-658.76	6111.71	3232.3	24
H00T	7923.51	6727.41	2753.05	23
H17	-1253.91	7812.5	3233.39	24
H00W	5577.7	4616.94	2481.74	24
H16	-69.18	9107.3	3449.34	26
H00Y	8778.94	6435.23	2163.51	29
H00Z	6466.29	4307.76	1896.96	30
H010	8048.07	5229.78	1736.35	32
H3A	1630(20)	6882(18)	4263(7)	22(6)
H4A	5930(20)	5200(20)	4331(8)	34(7)
H3B	2480(20)	6341(19)	4533(8)	29(6)
H4B	5660(30)	4960(20)	4742(9)	53(8)

Table S11. Solvent masks information for compound 3d.

Number	X	Y	Z	Volume	Electron count
1	-0.686	0.000	0.500	502.6	176.1
2	-0.853	0.500	0.000	502.6	176.1

Experimental

Single crystals of $C_{25}H_{18}Cl_2N_4OS_2$ (compound **3d**) were prepared by slow evaporation of saturated solution in DMSO. A suitable crystal was selected and mounted on the glass stick by acrylic glue on a SuperNova, Dual, Cu at home/near, AtlasS2 diffractometer. The crystal was kept at 100.00(10) K during data collection. Using Olex2 [1], the structure was solved with the SHELXT [2] structure solution program using Intrinsic Phasing and refined with the SHELXL [3] refinement package using Least Squares minimisation.

1. Dolomanov, O.V., Bourhis, L.J., Gildea, R.J, Howard, J.A.K. & Puschmann, H. (2009), J. Appl. Cryst. 42, 339-341.
2. Sheldrick, G.M. (2015). Acta Cryst. A71, 3-8.
3. Sheldrick, G.M. (2015). Acta Cryst. C71, 3-8.

Crystal structure determination details for compound 3d.

Crystal Data for $C_{25}H_{18}Cl_2N_4OS_2$ (M =525.45 g/mol): monoclinic, space group I2/a (no. 15), a = 11.97350(10) Å, b = 12.96920(10) Å, c = 34.4739(2) Å, β = 90.7890(10)°, V = 5352.83(7) Å^3, Z = 8, T = 100.00(10) K, μ(Cu Kα) = 3.837 mm^{-1}, D_{calc} = 1.304 g/cm^3, 29542 reflections measured (7.282° ≤ 2Θ ≤ 153.23°), 5572 unique (R_{int} = 0.0263, R_{sigma} = 0.0158) which were used in all calculations. The final R_1 was 0.0352 (I > 2σ(I)) and wR_2 was 0.0939 (all data).

Refinement model description

Number of restraints - 0, number of constraints - unknown.

Details:

1. Fixed Uiso
 At 1.2 times of:
  All C(H) groups
2.a Ternary CH refined with riding coordinates:
 C6(H6)
2.b Aromatic/amide H refined with riding coordinates:
 C13(H13), C19(H19), C15(H15), C12(H12), C10(H10), C18(H18), C00T(H00T), C17(H17), C00W(H00W), C16(H16), C00Y(H00Y), C00Z(H00Z), C010(H010)

Figure S17. HRMS data for compound 3a

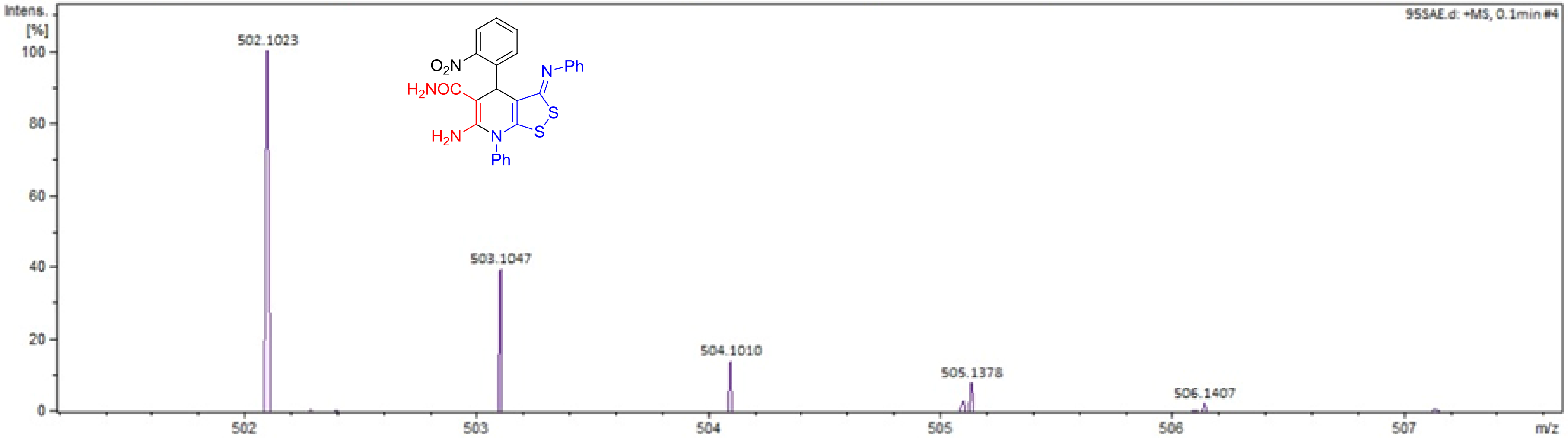

Figure S18. HRMS data for compound 3b

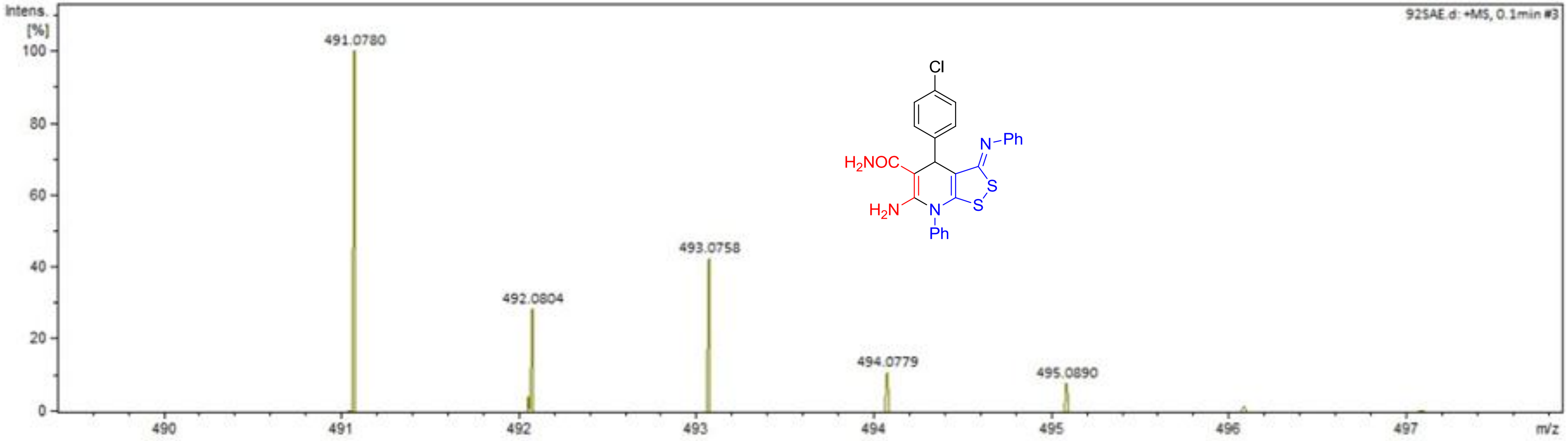

Figure S19. HRMS data for compound 3c

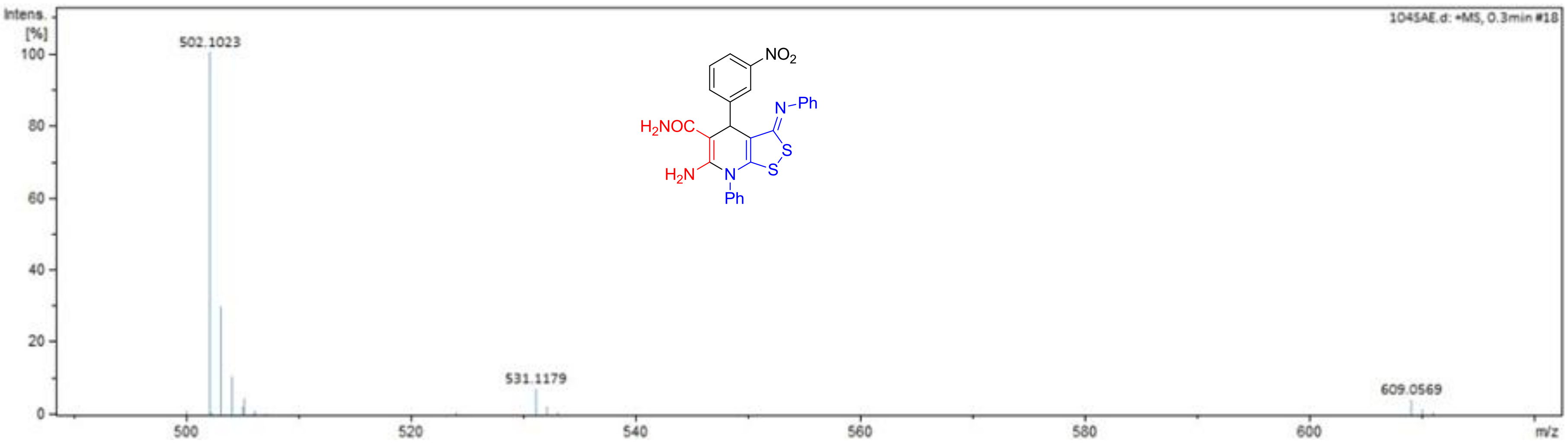

Figure S20. HRMS data for compound 3d

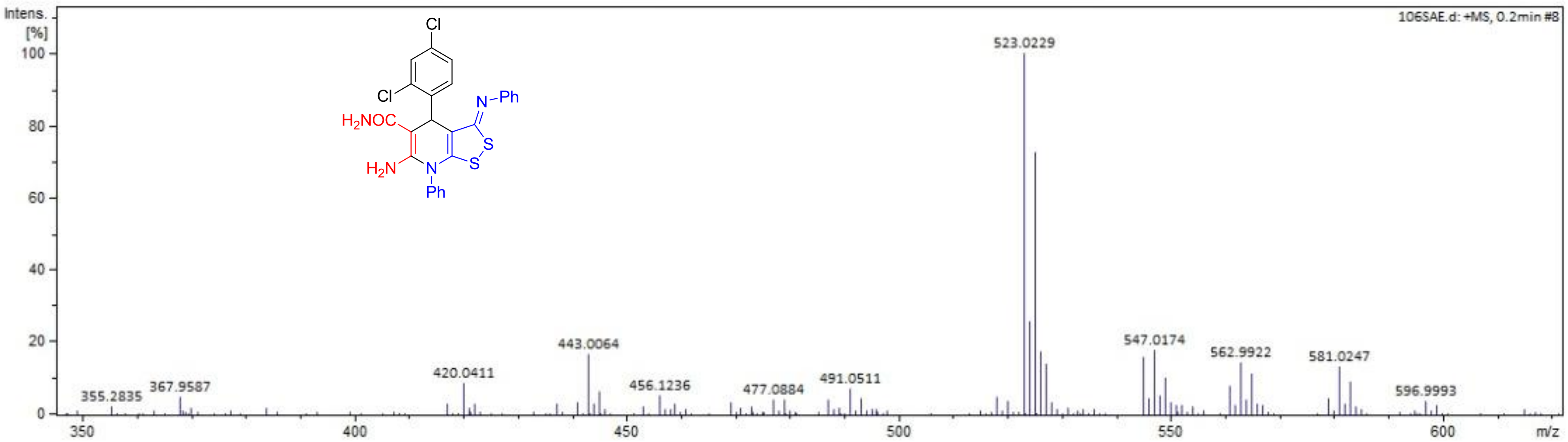

Figure S21. HRMS data for the crude mixture from the experiment (3) - reaction of 3-(4-methoxyphenyl)-2-cyanoacrylamide 2f with dithiomalondianilide 1 taken in the ratio 2 : 1;

Here we can see the peak of 3-(4-methoxyphenyl)-2-cyanoacrylamide **2f** [M+Na]+, calc. 225.063998, peaks of **3f** [M+Na]+, calc. 508.100365

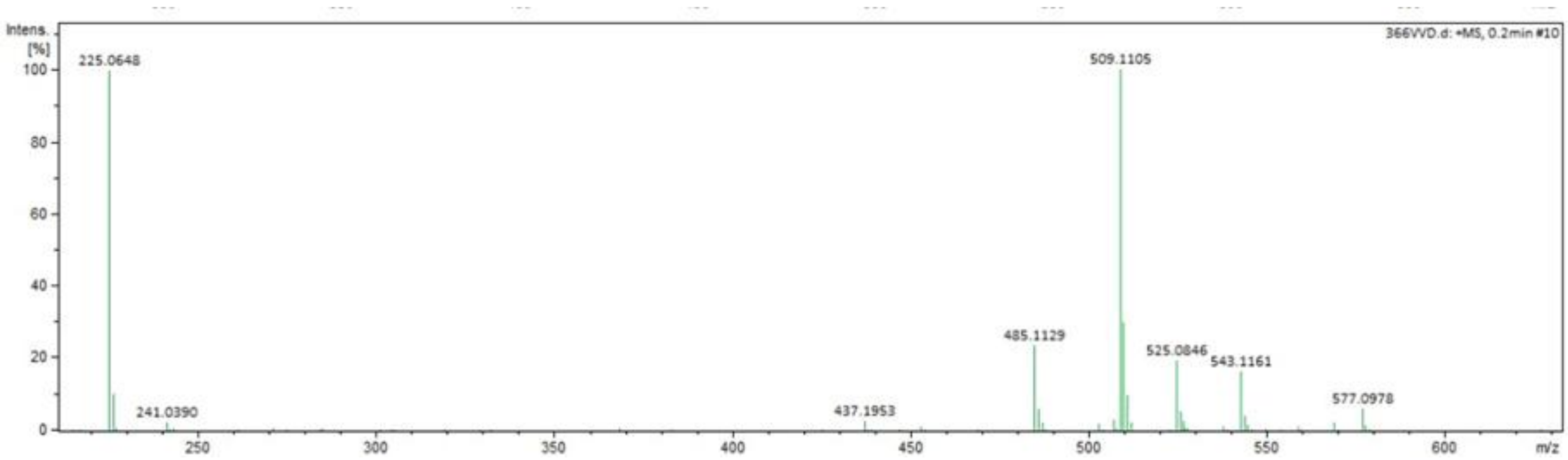

Figure S22. HRMS of pure dithiolopyridine 3f (experiment (2), Table 1, entry 6).

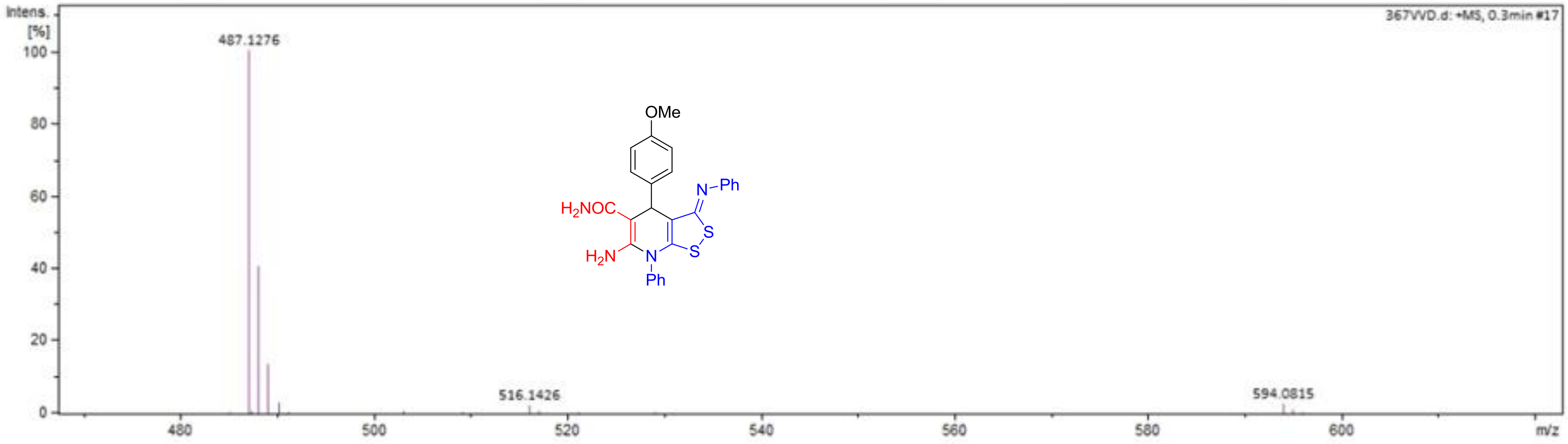

Table S12. Toxicity risks and physicochemical parameters of compounds 3a-f predicted using OSIRIS Property Explorer

Compound	Toxicity risk*				Physico-chemical parameters					
	A	B	C	D	*cLogP*	*logS*	MW	TPSA	Drug likeness	Drug Score
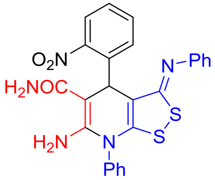 **3a**	–	–	–	–	3.65	-7.19	501	181.1	-3.29	0.19
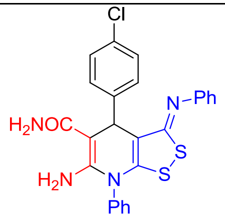 **3b**	–	–	–	–	5.18	-7.46	490	135.3	5.41	0.3
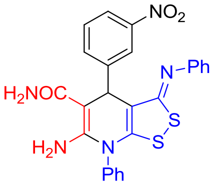 **3c**	–	–	–	–	3.65	-7.19	501	181.1	-1.32	0.22
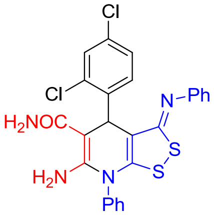 **3d**	–	–	–	–	5.79	-8.2	524	135.3	5.22	0.24

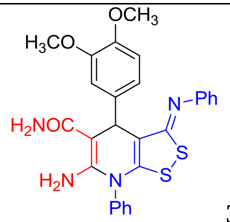 **3e**	–	–	–	–	4.43	-6.76	516	153.7	5.86	0.34
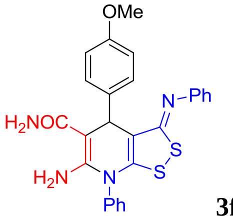 **3f**	–	–	–	–	4.5	-6.75	486	144.5	4.29	0.36

*(+) indicates high risk of toxicity, (±) moderate risk, (–) no toxicity predicted. A - Mutagenic, B - Tumorigenic, C - Irritant effects, D - Reproductive effects.

Table S13. ADMET parameters calculated for compound 3a using SwissADME and AdmetSar

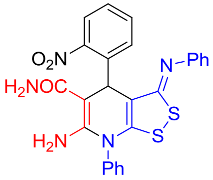

3a

NC1=C(C(N)=O)C(C2=CC=CC=C2[N+]([O-])=O)C3=C(SS/C3=N\C4=CC=CC=C4)N1C5=CC=CC=C5

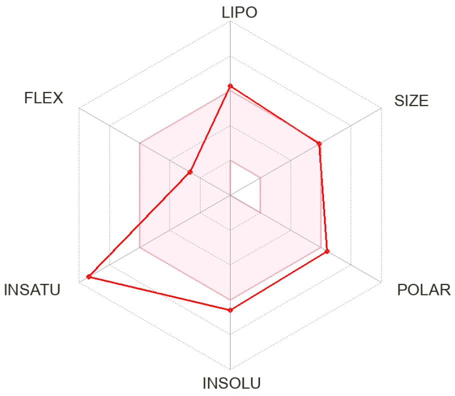

Physicochemical Properties
Formula C25H19ClN4OS2

Molecular weight	491.03 g/mol
Num. heavy atoms	33
Num. arom. heavy atoms	23
Fraction Csp3	0.04
Num. rotatable bonds	4
Num. H-bond acceptors	2
Num. H-bond donors	2
Molar Refractivity	138.34
TPSA	141.19 Å²
Lipophilicity	
Log Po/w (iLOGP)	3.14
Log Po/w (XLOGP3)	5.49
Log Po/w (WLOGP)	5.25
Log Po/w (MLOGP)	3.45

Log Po/w (SILICOS-IT)	6.17
Consensus Log Po/w	4.70
Water Solubility	
Log S (ESOL)	-6.59
Solubility	1.25e-04 mg/ml ; 2.54e-07 mol/l
Class	Poorly soluble
Log S (Ali)	-8.21
Solubility	3.01e-06 mg/ml ; 6.12e-09 mol/l
Class	Poorly soluble
Log S (SILICOS-IT)	-8.54
Solubility	1.42e-06 mg/ml ; 2.88e-09 mol/l
Class	Poorly soluble
Pharmacokinetics	
GI absorption	Low
BBB permeant	No
P-gp substrate	No
CYP1A2 inhibitor	No
CYP2C19 inhibitor	Yes
CYP2C9 inhibitor	Yes
CYP2D6 inhibitor	No
CYP3A4 inhibitor	Yes
Log Kp (skin permeation)	-5.40 cm/s
Druglikeness	
Lipinski	Yes; 0 violation
Ghose	No; 2 violations: MW>480, MR>130
Veber	No; 1 violation: TPSA>140
Egan	No; 1 violation: TPSA>131.6
Muegge	No; 1 violation: XLOGP3>5
Bioavailability Score	0.55
Medicinal Chemistry	
PAINS	0 alert
Brenk	0 alert
Leadlikeness	No; 2 violations: MW>350, XLOGP3>3.5
Synthetic accessibility	4.74

ADMET predicted profile	Value	Probability
Human Intestinal Absorption	+	0.9079
Caco-2	-	0.5591
Blood Brain Barrier	+	0.6000
Human oral bioavailability	+	0.6857
Subcellular localzation	Mitochondria	0.5405

OATP2B1 inhibitior	-	0.7131
OATP1B1 inhibitior	+	0.8928
OATP1B3 inhibitior	+	0.9368
MATE1 inhibitior	-	0.9623
OCT2 inhibitior	-	0.8750
BSEP inhibitior	+	0.6455
P-glycoprotein inhibitior	+	0.5850
P-glycoprotein substrate	-	0.7113
CYP3A4 substrate	+	0.5948
CYP2C9 substrate	-	0.8063
CYP2D6 substrate	-	0.8829
CYP3A4 inhibition	+	0.8782
CYP2C9 inhibition	+	0.5819
CYP2C19 inhibition	+	0.6868
CYP2D6 inhibition	-	0.7844
CYP1A2 inhibition	+	0.5784
CYP inhibitory promiscuity	+	0.9301
UGT catelyzed	-	0.0000
Carcinogenicity (binary)	-	0.6800
Carcinogenicity (trinary)	Danger	0.4377
Eye corrosion	-	0.9769
Eye irritation	-	0.9188
Ames mutagenesis	+	0.6209
Human Ether-a-go-go-Related Gene inhibition	-	0.6058
Micronuclear	+	1.0000
Hepatotoxicity	+	0.8000
skin sensitisation	-	0.8303
Respiratory toxicity	+	0.7778
Reproductive toxicity	+	0.7667
Mitochondrial toxicity	+	0.9000
Nephrotoxicity	+	0.5337
Acute Oral Toxicity (c)	III	0.6153
Estrogen receptor binding	+	0.7667
Androgen receptor binding	+	0.7434
Thyroid receptor binding	+	0.5635
Glucocorticoid receptor binding	+	0.8118
Aromatase binding	+	0.6500
PPAR gamma	+	0.6055

Honey bee toxicity	-	0.8944
Biodegradation	-	0.9250
Crustacea aquatic toxicity	-	0.6700
Fish aquatic toxicity	+	0.9820
Water solubility	-3.442	logS
Plasma protein binding	1.157	100%
Acute Oral Toxicity	2.881	log(1/(mol/kg))
Tetrahymena pyriformis	1.191	pIGC50 (ug/L)

Table S14. ADMET parameters calculated for compound 3b using SwissADME and AdmetSar

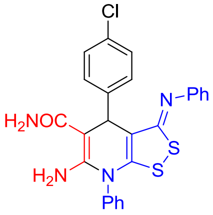

3b

NC1=C(C(N)=O)C(C2=CC=C(Cl)C=C2)C3=C(SS/C3=N\C4=CC=CC=C4)N1C5=CC=CC=C5

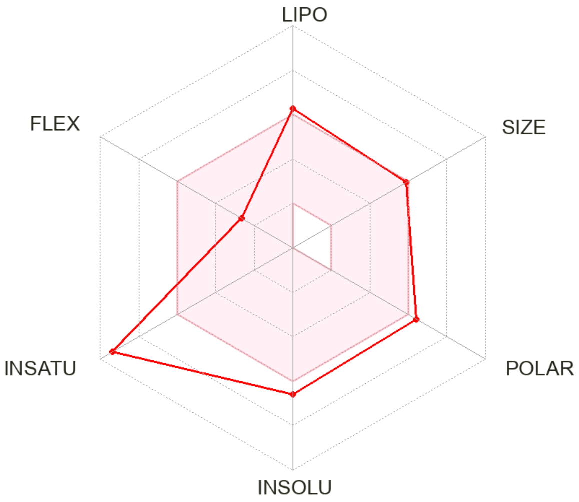

Physicochemical Properties

Formula	$C_{25}H_{19}ClN_4OS_2$
Molecular weight	491.03 g/mol
Num. heavy atoms	33
Num. arom. heavy atoms	23
Fraction Csp3	0.04
Num. rotatable bonds	4
Num. H-bond acceptors	2
Num. H-bond donors	2
Molar Refractivity	138.34

TPSA	141.19 Å²
Lipophilicity	
Log Po/w (iLOGP)	3.14
Log Po/w (XLOGP3)	5.49
Log Po/w (WLOGP)	5.25
Log Po/w (MLOGP)	3.45
Log Po/w (SILICOS-IT)	6.17
Consensus Log Po/w	4.70
Water Solubility	
Log S (ESOL)	-6.59
Solubility	1.25e-04 mg/ml ; 2.54e-07 mol/l
Class	Poorly soluble
Log S (Ali)	-8.21
Solubility	3.01e-06 mg/ml ; 6.12e-09 mol/l
Class	Poorly soluble
Log S (SILICOS-IT)	-8.54
Solubility	1.42e-06 mg/ml ; 2.88e-09 mol/l
Class	Poorly soluble
Pharmacokinetics	
GI absorption	Low
BBB permeant	No
P-gp substrate	No
CYP1A2 inhibitor	No
CYP2C19 inhibitor	Yes
CYP2C9 inhibitor	Yes
CYP2D6 inhibitor	No
CYP3A4 inhibitor	Yes
Log Kp (skin permeation)	-5.40 cm/s
Druglikeness	
Lipinski	Yes; 0 violation
Ghose	No; 2 violations: MW>480, MR>130
Veber	No; 1 violation: TPSA>140
Egan	No; 1 violation: TPSA>131.6
Muegge	No; 1 violation: XLOGP3>5
Bioavailability Score	0.55
Medicinal Chemistry	
PAINS	0 alert
Brenk	0 alert
Leadlikeness	No; 2 violations: MW>350, XLOGP3>3.5
Synthetic accessibility	4.74

ADMET predicted profile	Value	Probability
Human Intestinal Absorption	+	0.9699
Caco-2	-	0.6350
Blood Brain Barrier	+	0.8250
Human oral bioavailability	-	0.5000
Subcellular localzation	Lysosomes	0.6760
OATP2B1 inhibitior	-	0.7128
OATP1B1 inhibitior	+	0.9030
OATP1B3 inhibitior	+	0.9377
MATE1 inhibitior	-	0.9823
OCT2 inhibitior	-	0.7250
BSEP inhibitior	+	0.9473
P-glycoprotein inhibitior	+	0.6827
P-glycoprotein substrate	-	0.8936
CYP3A4 substrate	+	0.5761
CYP2C9 substrate	+	0.6000
CYP2D6 substrate	-	0.8859
CYP3A4 inhibition	+	0.6438
CYP2C9 inhibition	+	0.6632
CYP2C19 inhibition	+	0.8129
CYP2D6 inhibition	-	0.8238
CYP1A2 inhibition	+	0.6461
CYP inhibitory promiscuity	+	0.9560
UGT catelyzed	-	0.0000
Carcinogenicity (binary)	-	0.7119
Carcinogenicity (trinary)	Non-required	0.5554
Eye corrosion	-	0.9794
Eye irritation	-	0.9813
Ames mutagenesis	-	0.5454
Human Ether-a-go-go-Related Gene inhibition	-	0.4305
Micronuclear	+	0.9100
Hepatotoxicity	+	0.6375
skin sensitisation	-	0.8416
Respiratory toxicity	+	0.8111
Reproductive toxicity	+	0.8111
Mitochondrial toxicity	+	0.8625
Nephrotoxicity	+	0.4594
Acute Oral Toxicity (c)	III	0.5895
Estrogen receptor binding	+	0.7714
Androgen receptor binding	+	0.7495
Thyroid receptor binding	+	0.6576

Glucocorticoid receptor binding	+	0.8577
Aromatase binding	+	0.6429
PPAR gamma	+	0.6563
Honey bee toxicity	-	0.8830
Biodegradation	-	0.8500
Crustacea aquatic toxicity	-	0.5200
Fish aquatic toxicity	+	0.9874
Water solubility	-3.891	logS
Plasma protein binding	1.178	100%
Acute Oral Toxicity	2.027	log(1/(mol/kg))
Tetrahymena pyriformis	1.787	pIGC50 (ug/L)

Table S15. ADMET parameters calculated for compound 3c using SwissADME and AdmetSar

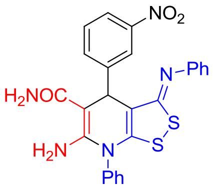

3c

NC1=C(C(N)=O)C(C2=CC([N+]([O-])=O)=CC=C2)C3=C(SS/C3=N\C4=CC=CC=C4)N1C5=CC=CC=C5

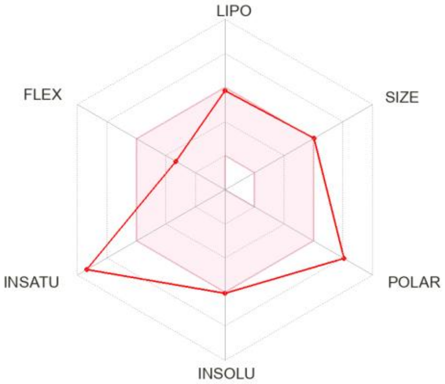

Formula	C25H19N5O3S2
Molecular weight	501.58 g/mol
Num. heavy atoms	35
Num. arom. heavy atoms	23
Fraction Csp3	0.04
Num. rotatable bonds	5
Num. H-bond acceptors	4
Num. H-bond donors	2
Molar Refractivity	142.16
TPSA	187.01 Å²
Lipophilicity	
Log Po/w (iLOGP)	2.77
Log Po/w (XLOGP3)	4.69
Log Po/w (WLOGP)	4.51
Log Po/w (MLOGP)	2.06

Log Po/w (SILICOS-IT) 3.37
Consensus Log Po/w 3.48
Water Solubility
Log S (ESOL) -6.06
Solubility 4.36e-04 mg/ml ; 8.69e-07 mol/l
Class Poorly soluble
Log S (Ali) -8.35
Solubility 2.27e-06 mg/ml ; 4.52e-09 mol/l
Class Poorly soluble
Log S (SILICOS-IT) -7.29
Solubility 2.56e-05 mg/ml ; 5.11e-08 mol/l
Class Poorly soluble
Pharmacokinetics
GI absorption Low
BBB permeant No
P-gp substrate No
CYP1A2 inhibitor No
CYP2C19 inhibitor Yes
CYP2C9 inhibitor Yes
CYP2D6 inhibitor No
CYP3A4 inhibitor Yes
Log Kp (skin permeation) -6.03 cm/s
Druglikeness
Lipinski Yes; 1 violation: MW>500
Ghose No; 2 violations: MW>480, MR>130
Veber No; 1 violation: TPSA>140
Egan No; 1 violation: TPSA>131.6
Muegge No; 1 violation: TPSA>150
Bioavailability Score 0.55
Medicinal Chemistry
PAINS 0 alert
Brenk 2 alerts: nitro_group, oxygen-nitrogen_single_bond
Leadlikeness No; 2 violations: MW>350, XLOGP3>3.5
Synthetic accessibility 4.85

ADMET predicted profile	Value	Probability
Human Intestinal Absorption	+	0.9079
Caco-2	-	0.6981
Blood Brain Barrier	+	0.6000
Human oral bioavailability	-	0.5143
Subcellular localzation	Mitochondria	0.5405

OATP2B1 inhibitior	-	0.7151
OATP1B1 inhibitior	+	0.8918
OATP1B3 inhibitior	+	0.9368
MATE1 inhibitior	-	0.9623
OCT2 inhibitior	-	0.8750
BSEP inhibitior	+	0.7898
P-glycoprotein inhibitior	+	0.6185
P-glycoprotein substrate	-	0.6669
CYP3A4 substrate	+	0.6186
CYP2C9 substrate	-	0.8063
CYP2D6 substrate	-	0.8829
CYP3A4 inhibition	+	0.8782
CYP2C9 inhibition	+	0.5819
CYP2C19 inhibition	+	0.6868
CYP2D6 inhibition	-	0.7844
CYP1A2 inhibition	+	0.5784
CYP inhibitory promiscuity	+	0.9301
UGT catelyzed	-	0.0000
Carcinogenicity (binary)	-	0.6800
Carcinogenicity (trinary)	Danger	0.4377
Eye corrosion	-	0.9769
Eye irritation	-	0.9467
Ames mutagenesis	+	0.7009
Human Ether-a-go-go-Related Gene inhibition	-	0.5000
Micronuclear	+	1.0000
Hepatotoxicity	+	0.7875
skin sensitisation	-	0.8303
Respiratory toxicity	+	0.7111
Reproductive toxicity	+	0.7667
Mitochondrial toxicity	+	0.9000
Nephrotoxicity	-	0.6160
Acute Oral Toxicity (c)	III	0.6153
Estrogen receptor binding	+	0.8047
Androgen receptor binding	+	0.7615
Thyroid receptor binding	+	0.5894
Glucocorticoid receptor binding	+	0.8449
Aromatase binding	+	0.5873
PPAR gamma	+	0.6930
Honey bee toxicity	-	0.8937

Biodegradation	-	0.9000
Crustacea aquatic toxicity	-	0.8300
Fish aquatic toxicity	+	0.9820
Water solubility	-3.442	logS
Plasma protein binding	1.244	100%
Acute Oral Toxicity	2.587	log(1/(mol/kg))
Tetrahymena pyriformis	1.256	pIGC50 (ug/L)

Table S16. ADMET parameters calculated for compound 3d using SwissADME and AdmetSar

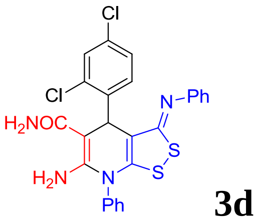

3d

NC1=C(C(N)=O)C(C2=CC=C(Cl)C=C2Cl)C3=C(SS/C3=N\C4=CC=CC=C4)N1C5=CC=CC=C5

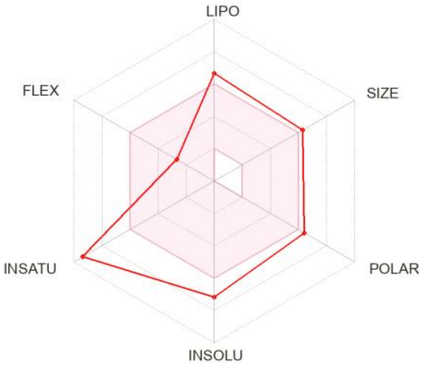

Physicochemical Properties
Formula C25H18Cl2N4OS2
Molecular weight 525.47 g/mol
Num. heavy atoms 34
Num. arom. heavy atoms 23
Fraction Csp3 0.04
Num. rotatable bonds 4

Num. H-bond acceptors	2
Num. H-bond donors	2
Molar Refractivity	143.35
TPSA	141.19 Å²
Lipophilicity	
Log Po/w (iLOGP)	3.06
Log Po/w (XLOGP3)	6.12
Log Po/w (WLOGP)	5.91
Log Po/w (MLOGP)	3.92
Log Po/w (SILICOS-IT)	6.82
Consensus Log Po/w	5.16
Water Solubility	
Log S (ESOL)	-7.19
Solubility	3.39e-05 mg/ml ; 6.45e-08 mol/l
Class	Poorly soluble
Log S (Ali)	-8.87
Solubility	7.14e-07 mg/ml ; 1.36e-09 mol/l
Class	Poorly soluble
Log S (SILICOS-IT)	-9.12
Solubility	4.01e-07 mg/ml ; 7.64e-10 mol/l
Class	Poorly soluble
Pharmacokinetics	
GI absorption	Low
BBB permeant	No
P-gp substrate	No
CYP1A2 inhibitor	No
CYP2C19 inhibitor	Yes
CYP2C9 inhibitor	Yes
CYP2D6 inhibitor	No
CYP3A4 inhibitor	Yes
Log Kp (skin permeation)	-5.16 cm/s
Druglikeness	
Lipinski	Yes; 1 violation: MW>500
Ghose	No; 3 violations: MW>480, WLOGP>5.6, MR>130
Veber	No; 1 violation: TPSA>140
Egan	No; 2 violations: WLOGP>5.88, TPSA>131.6
Muegge	No; 1 violation: XLOGP3>5
Bioavailability Score	0.55
Medicinal Chemistry	
PAINS	0 alert

Brenk 0 alert
Leadlikeness No; 2 violations: MW>350, XLOGP3>3.5
Synthetic accessibility 4.79

**ADMET predicted profile**	**Value**	**Probability**
Human Intestinal Absorption	+	0.9699
Caco-2	-	0.6591
Blood Brain Barrier	+	0.8250
Human oral bioavailability	+	0.6571
Subcellular localzation	Lysosomes	0.6760
OATP2B1 inhibitior	-	0.7128
OATP1B1 inhibitior	+	0.9163
OATP1B3 inhibitior	+	0.9377
MATE1 inhibitior	-	0.9823
OCT2 inhibitior	-	0.7250
BSEP inhibitior	+	0.9631
P-glycoprotein inhibitior	+	0.6805
P-glycoprotein substrate	-	0.7919
CYP3A4 substrate	+	0.6242
CYP2C9 substrate	+	0.6000
CYP2D6 substrate	-	0.8859
CYP3A4 inhibition	+	0.6438
CYP2C9 inhibition	+	0.6632
CYP2C19 inhibition	+	0.8129
CYP2D6 inhibition	-	0.8238
CYP1A2 inhibition	+	0.6461
CYP inhibitory promiscuity	+	0.9560
UGT catelyzed	-	0.0000
Carcinogenicity (binary)	-	0.7119
Carcinogenicity (trinary)	Non-required	0.5554
Eye corrosion	-	0.9794
Eye irritation	-	0.9749
Ames mutagenesis	+	0.5046
Human Ether-a-go-go-Related Gene inhibition	-	0.5000
Micronuclear	+	0.9100
Hepatotoxicity	+	0.8000
skin sensitisation	-	0.8416
Respiratory toxicity	+	0.7667
Reproductive toxicity	+	0.8111
Mitochondrial toxicity	+	0.8625

Nephrotoxicity	+	0.5694
Acute Oral Toxicity (c)	III	0.5895
Estrogen receptor binding	+	0.8165
Androgen receptor binding	+	0.7653
Thyroid receptor binding	+	0.7229
Glucocorticoid receptor binding	+	0.8848
Aromatase binding	+	0.6064
PPAR gamma	+	0.7571
Honey bee toxicity	-	0.8920
Biodegradation	-	0.8500
Crustacea aquatic toxicity	-	0.5051
Fish aquatic toxicity	+	0.9874
Water solubility	-3.891	logS
Plasma protein binding	1.134	100%
Acute Oral Toxicity	2.068	log(1/(mol/kg))
Tetrahymena pyriformis	1.736	pIGC50 (ug/L)

Table S17. ADMET parameters calculated for compound 3e using SwissADME and AdmetSar

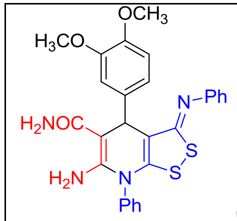

3e

NC1=C(C(N)=O)C(C2=CC=C(OC)C(OC)=C2)C3=C(SS/C3=N\C4=CC=CC=C4)N1C5=CC=CC=C5

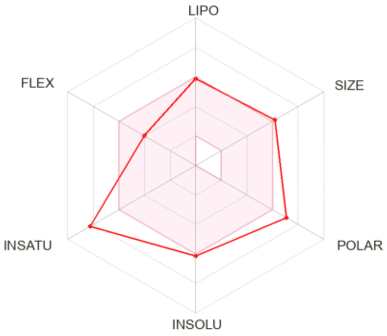

Physicochemical Properties

Formula	C27H24N4O3S2
Molecular weight	516.63 g/mol
Num. heavy atoms	36
Num. arom. heavy atoms	23
Fraction Csp3	0.11
Num. rotatable bonds	6
Num. H-bond acceptors	4
Num. H-bond donors	2
Molar Refractivity	146.32
TPSA	159.65 Å²
Lipophilicity	
Log Po/w (iLOGP)	3.69
Log Po/w (XLOGP3)	4.81
Log Po/w (WLOGP)	4.62
Log Po/w (MLOGP)	2.30
Log Po/w (SILICOS-IT)	5.67
Consensus Log Po/w	4.22
Water Solubility	
Log S (ESOL)	-6.15
Solubility	3.66e-04 mg/ml ; 7.08e-07 mol/l
Class	Poorly soluble
Log S (Ali)	-7.90
Solubility	6.58e-06 mg/ml ; 1.27e-08 mol/l
Class	Poorly soluble
Log S (SILICOS-IT)	-8.15
Solubility	3.65e-06 mg/ml ; 7.06e-09 mol/l
Class	Poorly soluble
Pharmacokinetics	
GI absorption	Low
BBB permeant	No
P-gp substrate	No
CYP1A2 inhibitor	No
CYP2C19 inhibitor	Yes
CYP2C9 inhibitor	Yes
CYP2D6 inhibitor	No
CYP3A4 inhibitor	Yes
Log Kp (skin permeation)	-6.04 cm/s
Druglikeness	
Lipinski	Yes; 1 violation: MW>500
Ghose	No; 2 violations: MW>480, MR>130

Veber No; 1 violation: TPSA>140<br>Egan No; 1 violation: TPSA>131.6<br>Muegge No; 1 violation: TPSA>150<br>Bioavailability Score 0.55<br>Medicinal Chemistry<br>PAINS 0 alert<br>Brenk 0 alert<br>Leadlikeness No; 2 violations: MW>350, XLOGP3>3.5<br>Synthetic accessibility 5.00		
**ADMET predicted profile**	**Value**	**Probability**
Human Intestinal Absorption	+	0.9612
Caco-2	-	0.5433
Blood Brain Barrier	+	0.5500
Human oral bioavailability	-	0.5000
Subcellular localzation	Mitochondria	0.3736
OATP2B1 inhibitior	-	0.7117
OATP1B1 inhibitior	+	0.9128
OATP1B3 inhibitior	+	0.9359
MATE1 inhibitior	-	0.9423
OCT2 inhibitior	-	0.9000
BSEP inhibitior	+	0.9346
P-glycoprotein inhibitior	+	0.9013
P-glycoprotein substrate	-	0.6867
CYP3A4 substrate	+	0.5925
CYP2C9 substrate	-	0.7952
CYP2D6 substrate	-	0.8665
CYP3A4 inhibition	+	0.5202
CYP2C9 inhibition	+	0.6508
CYP2C19 inhibition	+	0.7677
CYP2D6 inhibition	-	0.8155
CYP1A2 inhibition	-	0.6840
CYP inhibitory promiscuity	+	0.8506
UGT catelyzed	-	0.0000
Carcinogenicity (binary)	-	0.8600
Carcinogenicity (trinary)	Non-required	0.4764
Eye corrosion	-	0.9816
Eye irritation	-	0.9617
Ames mutagenesis	+	0.5146
Human Ether-a-go-go-Related Gene inhibition	+	0.7191

Micronuclear	+	0.9600
Hepatotoxicity	-	0.5125
skin sensitisation	-	0.8429
Respiratory toxicity	+	0.7000
Reproductive toxicity	+	0.7889
Mitochondrial toxicity	+	0.8750
Nephrotoxicity	-	0.7047
Acute Oral Toxicity (c)	III	0.5792
Estrogen receptor binding	+	0.8648
Androgen receptor binding	+	0.7423
Thyroid receptor binding	+	0.7647
Glucocorticoid receptor binding	+	0.8746
Aromatase binding	-	0.5000
PPAR gamma	+	0.7150
Honey bee toxicity	-	0.8936
Biodegradation	-	0.9250
Crustacea aquatic toxicity	-	0.6600
Fish aquatic toxicity	+	0.9670
Water solubility	-3.438	logS
Plasma protein binding	1.077	100%
Acute Oral Toxicity	2.045	log(1/(mol/kg))
Tetrahymena pyriformis	1.404	pIGC50 (ug/L)

Table S18. ADMET parameters calculated for compound 3f using SwissADME and AdmetSar

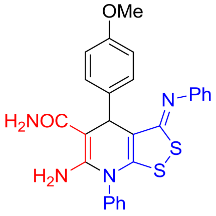

3f

NC1=C(C(N)=O)C(C2=CC=C(OC)C=C2)C3=C(SS/C3=N\C4=CC=CC=C4)N1C5=CC=CC=C5

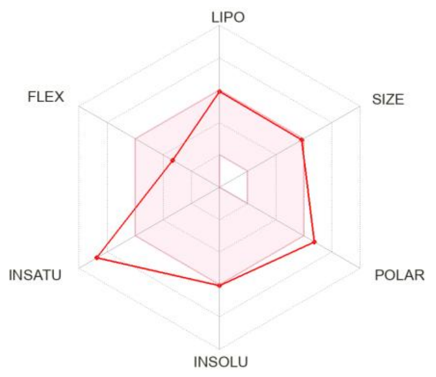

Physicochemical Properties
Formula C26H22N4O2S2
Molecular weight 486.61 g/mol
Num. heavy atoms 34
Num. arom. heavy atoms 23
Fraction Csp3 0.08
Num. rotatable bonds 5
Num. H-bond acceptors 3
Num. H-bond donors 2
Molar Refractivity 139.83
TPSA 150.42 Å²
Lipophilicity
Log Po/w (iLOGP) 3.60
Log Po/w (XLOGP3) 4.83
Log Po/w (WLOGP) 4.61
Log Po/w (MLOGP) 2.63
Log Po/w (SILICOS-IT) 5.60
Consensus Log Po/w 4.26
Water Solubility
Log S (ESOL) -6.07
Solubility 4.14e-04 mg/ml ; 8.50e-07 mol/l
Class Poorly soluble
Log S (Ali) -7.72
Solubility 9.23e-06 mg/ml ; 1.90e-08 mol/l
Class Poorly soluble
Log S (SILICOS-IT) -8.06
Solubility 4.27e-06 mg/ml ; 8.77e-09 mol/l

Class Poorly soluble

Pharmacokinetics

GI absorption Low

BBB permeant No

P-gp substrate No

CYP1A2 inhibitor No

CYP2C19 inhibitor Yes

CYP2C9 inhibitor Yes

CYP2D6 inhibitor No

CYP3A4 inhibitor Yes

Log Kp (skin permeation) -5.84 cm/s

Druglikeness

Lipinski Yes; 0 violation

Ghose No; 2 violations: MW>480, MR>130

Veber No; 1 violation: TPSA>140

Egan No; 1 violation: TPSA>131.6

Muegge No; 1 violation: TPSA>150

Bioavailability Score 0.55

Medicinal Chemistry

PAINS 0 alert

Brenk 0 alert

Leadlikeness No; 2 violations: MW>350, XLOGP3>3.5

Synthetic accessibility 4.84

**ADMET predicted profile**	**Value**	**Probability**
Human Intestinal Absorption	+	0.9786
Caco-2	-	0.5455
Blood Brain Barrier	+	0.6750
Human oral bioavailability	-	0.6000
Subcellular localzation	Lysosomes	0.4007
OATP2B1 inhibitior	-	0.7108
OATP1B1 inhibitior	+	0.8985
OATP1B3 inhibitior	+	0.9369
MATE1 inhibitior	-	0.9423
OCT2 inhibitior	-	0.8000
BSEP inhibitior	+	0.9310
P-glycoprotein inhibitior	+	0.8414
P-glycoprotein substrate	-	0.8429
CYP3A4 substrate	+	0.5825
CYP2C9 substrate	-	0.7952
CYP2D6 substrate	-	0.8665

CYP3A4 inhibition	-	0.5564
CYP2C9 inhibition	+	0.6211
CYP2C19 inhibition	+	0.7777
CYP2D6 inhibition	-	0.7906
CYP1A2 inhibition	-	0.5000
CYP inhibitory promiscuity	+	0.8677
UGT catelyzed	-	0.0000
Carcinogenicity (binary)	-	0.8700
Carcinogenicity (trinary)	Non-required	0.4908
Eye corrosion	-	0.9818
Eye irritation	-	0.9569
Ames mutagenesis	+	0.5246
Human Ether-a-go-go-Related Gene inhibition	+	0.6740
Micronuclear	+	0.9500
Hepatotoxicity	-	0.5594
skin sensitisation	-	0.8413
Respiratory toxicity	+	0.7111
Reproductive toxicity	+	0.7444
Mitochondrial toxicity	+	0.8875
Nephrotoxicity	-	0.6658
Acute Oral Toxicity (c)	III	0.5659
Estrogen receptor binding	+	0.8366
Androgen receptor binding	+	0.7831
Thyroid receptor binding	+	0.7030
Glucocorticoid receptor binding	+	0.8841
Aromatase binding	+	0.5494
PPAR gamma	+	0.6600
Honey bee toxicity	-	0.9201
Biodegradation	-	0.8500
Crustacea aquatic toxicity	-	0.6000
Fish aquatic toxicity	+	0.9527
Water solubility	-3.538	logS
Plasma protein binding	1.016	100%
Acute Oral Toxicity	2.252	log(1/(mol/kg))
Tetrahymena pyriformis	1.371	pIGC50 (ug/L)

Table S19. Rat acute toxicity of 3a predicted by GUSAR

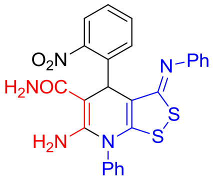

Rat IP LD50 Log10(mmol/kg)	Rat IV LD50 log10(mmol/kg)	Rat Oral LD50 log10(mmol/kg)	Rat SC LD50 log10(mmol/kg)
-0,004 in AD	-0,437 in AD	0,542 in AD	0,212 in AD

Rat IP LD50 (mg/kg)	Rat IV LD50 (mg/kg)	Rat Oral LD50 (mg/kg)	Rat SC LD50 (mg/kg)
497,200 in AD	183,400 in AD	1747,000 in AD	816,500 in AD

Acute Rodent Toxicity Classification of Chemicals by OECD Project

Rat IP LD50 Classification	Rat IV LD50 Classification	Rat Oral LD50 Classification	Rat SC LD50 Classification
Class 4 in AD	Class 4 in AD	Class 4 in AD	Class 4 in AD

IP - Intraperitoneal route of administration
IV - Intravenous route of administration
Oral - Oral route of administration
SC - Subcutaneous route of administration

in AD - compound falls in applicability domain of models
out of AD - compound is out of applicability domain of models

Table S20. Rat acute toxicity of 3b predicted by GUSAR

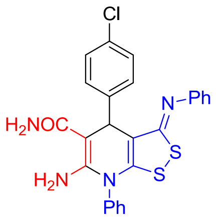

Rat IP LD50 Log10(mmol/kg)	Rat IV LD50 log10(mmol/kg)	Rat Oral LD50 log10(mmol/kg)	Rat SC LD50 log10(mmol/kg)
0,077 in AD	-0,442 in AD	0,499 in AD	0,583 out of AD

Rat IP LD50 (mg/kg)	Rat IV LD50 (mg/kg)	Rat Oral LD50 (mg/kg)	Rat SC LD50 (mg/kg)
586,000 in AD	177,500 in AD	1548,000 in AD	1881,000 out of AD

Acute Rodent Toxicity Classification of Chemicals by OECD Project

Rat IP LD50 Classification	Rat IV LD50 Classification	Rat Oral LD50 Classification	Rat SC LD50 Classification
Class 5 in AD	Class 4 in AD	Class 4 in AD	Class 5 out of AD

IP - Intraperitoneal route of administration
IV - Intravenous route of administration
Oral - Oral route of administration
SC - Subcutaneous route of administration

in AD - compound falls in applicability domain of models
out of AD - compound is out of applicability domain of models

Table S21. Rat acute toxicity of 3c predicted by GUSAR

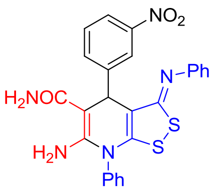

Rat IP LD50 Log10(mmol/kg)	Rat IV LD50 log10(mmol/kg)	Rat Oral LD50 log10(mmol/kg)	Rat SC LD50 log10(mmol/kg)
0,060 in AD	-0,380 in AD	0,512 in AD	0,179 out of AD

Rat IP LD50 (mg/kg)	Rat IV LD50 (mg/kg)	Rat Oral LD50 (mg/kg)	Rat SC LD50 (mg/kg)
575,400 in AD	209,100 in AD	1630,000 in AD	756,600 out of AD

Acute Rodent Toxicity Classification of Chemicals by OECD Project

Rat IP LD50 Classification	Rat IV LD50 Classification	Rat Oral LD50 Classification	Rat SC LD50 Classification
Class 5 in AD	Class 4 in AD	Class 4 in AD	Class 4 out of AD

IP - Intraperitoneal route of administration
IV - Intravenous route of administration
Oral - Oral route of administration
SC - Subcutaneous route of administration

in AD - compound falls in applicability domain of models
out of AD - compound is out of applicability domain of models

Table S22. Rat acute toxicity of 3d predicted by GUSAR

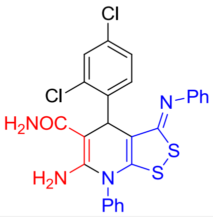

Rat IP LD50 Log10(mmol/kg)	Rat IV LD50 log10(mmol/kg)	Rat Oral LD50 log10(mmol/kg)	Rat SC LD50 log10(mmol/kg)
0,247 in AD	-0,562 in AD	0,463 in AD	0,541 out of AD

Rat IP LD50 (mg/kg)	Rat IV LD50 (mg/kg)	Rat Oral LD50 (mg/kg)	Rat SC LD50 (mg/kg)
927,100 in AD	144,100 in AD	1524,000 in AD	1826,000 out of AD

Acute Rodent Toxicity Classification of Chemicals by OECD Project

Rat IP LD50 Classification	Rat IV LD50 Classification	Rat Oral LD50 Classification	Rat SC LD50 Classification
Class 5 in AD	Class 4 in AD	Class 4 in AD	Class 5 out of AD

Table S23. Rat acute toxicity of 3e predicted by GUSAR

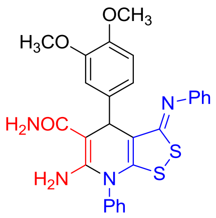

Rat IP LD50 Log10(mmol/kg)	Rat IV LD50 log10(mmol/kg)	Rat Oral LD50 log10(mmol/kg)	Rat SC LD50 log10(mmol/kg)
0,141 in AD	-0,388 in AD	0,470 in AD	0,422 in AD

Rat IP LD50 (mg/kg)	Rat IV LD50 (mg/kg)	Rat Oral LD50 (mg/kg)	Rat SC LD50 (mg/kg)
715,500 in AD	211,500 in AD	1524,000 in AD	1366,000 in AD

Acute Rodent Toxicity Classification of Chemicals by OECD Project

Rat IP LD50 Classification	Rat IV LD50 Classification	Rat Oral LD50 Classification	Rat SC LD50 Classification
Class 5 in AD	Class 4 in AD	Class 4 in AD	Class 5 in AD

IP - Intraperitoneal route of administration
IV - Intravenous route of administration
Oral - Oral route of administration
SC - Subcutaneous route of administration

in AD - compound falls in applicability domain of models
out of AD - compound is out of applicability domain of models

Table S24. Rat acute toxicity of 3f predicted by GUSAR

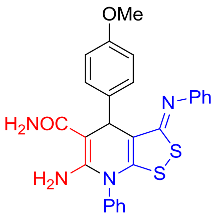

Rat IP LD50 Log10(mmol/kg)	Rat IV LD50 log10(mmol/kg)	Rat Oral LD50 log10(mmol/kg)	Rat SC LD50 log10(mmol/kg)
0,171 in AD	-0,224 in AD	0,534 in AD	0,403 out of AD

Rat IP LD50 (mg/kg)	Rat IV LD50 (mg/kg)	Rat Oral LD50 (mg/kg)	Rat SC LD50 (mg/kg)
721,700 in AD	290,300 in AD	1662,000 in AD	1232,000 out of AD

Acute Rodent Toxicity Classification of Chemicals by OECD Project

Rat IP LD50 Classification	Rat IV LD50 Classification	Rat Oral LD50 Classification	Rat SC LD50 Classification
Class 5 in AD	Class 4 in AD	Class 4 in AD	Class 5 out of AD

IP - Intraperitoneal route of administration
IV - Intravenous route of administration
Oral - Oral route of administration
SC - Subcutaneous route of administration

in AD - compound falls in applicability domain of models
out of AD - compound is out of applicability domain of models

Table S25. The predicted results of protein-ligand interaction for compounds 3a-f

Compound	Rank	PDB ID	UniProt ID	Predock score	Scoring functions ΔG_{bind}, kcal/mol	Final dock score
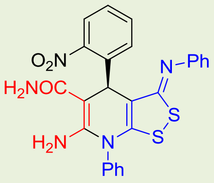 **(*R*)-3a**	1	6uvc_A	Q07817	0.184	-19.144	0.328
	2	4r0i_A	Q9Y5Y6	0.156	-21.667	0.319
	3	3k9x_B	P00742	0.120	-20.671	0.275
	4	4yt6_H	P08709	0.122	-19.541	0.268
	5	4gu6_A	Q05397	0.084	-24.434	0.267
	6	1pkd_A	P24941	0.089	-23.345	0.264
	7	3znr_A	Q8WUI4	0.074	-25.114	0.262
	8	Q7Z3E1_F1_d2	Q7Z3E1	0.068	-25.585	0.260
	9	2q7m_B,C	P20292, P20292	0.059	-26.706	0.259
	10	4iph_A	P27694	0.131	-17.007	0.259
	11	3khv_A	P00749	0.098	-21.235	0.257
	12	7wld_S,T,U	Q969N2, Q96S52, Q9H490	0.056	-26.640	0.256
	13	1trn_B	P07477	0.104	-19.967	0.253
	14	6r6y_A	O43570	0.083	-22.730	0.253
	15	2srt_A	P08254	0.070	-24.351	0.252
	16	7n9g_A,B	P00519, P00519	0.057	-25.985	0.252
	17	3ac8_A	P06239	0.073	-23.739	0.251
	18	4mnf_B	P15056	0.065	-24.744	0.251
	19	1q6s_A,B	P18031, P18031	0.068	-24.287	0.250
	20	8gv8_A	P04920	0.059	-25.498	0.250
	21	8bh3_A	P78527	0.057	-25.727	0.250
	22	7dtc_A	Q14524	0.060	-25.171	0.249
	23	1wbp_A	Q96SB4	0.066	-24.075	0.246
	24	7yaw_A	Q9NYL2	0.058	-25.093	0.246
	25	6kuw_A,B	P18825, P18825	0.056	-25.352	0.246
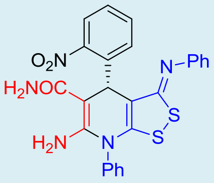 **(*S*)-3a**	1	6vwc_B	Q07817	0.170	-21.386	0.330
	2	2p93_A	P00742	0.133	-22.615	0.303
	3	4o97_A	Q9Y5Y6	0.137	-20.649	0.292
	4	4gu6_A	Q05397	0.091	-25.831	0.284
	5	1pkd_A	P24941	0.103	-23.745	0.281
	6	6r6y_A	O43570	0.102	-23.378	0.277
	7	3khv_A	P00749	0.108	-22.081	0.273
	8	4yt6_H	P08709	0.120	-19.960	0.269
	9	4fsl_E	P56817	0.098	-22.323	0.266
	10	Q5JU69_F1_d1	Q5JU69	0.078	-24.929	0.265
	11	4mnf_B	P15056	0.078	-24.719	0.263
	12	2srt_A	P08254	0.072	-25.422	0.263
	13	O15197_F1_d3	O15197	0.051	-28.272	0.263
	14	5qce_B	P25774	0.060	-26.449	0.258
	15	8evb_C	Q16281	0.082	-23.328	0.257
	16	2pqf_C	Q9H0J9	0.050	-27.574	0.257
	17	7yaw_A	Q9NYL2	0.070	-24.727	0.255
	18	1trn_B	P07477	0.104	-20.179	0.255
	19	7sxf_A	P49840	0.066	-25.200	0.255
	20	6glb_B	P52333	0.061	-25.764	0.254
	21	8hmy_E	Q92989	0.049	-27.318	0.254
	22	3cs9_B	P00519	0.099	-20.578	0.254

	23	6uel_B	P31327	0.056	-26.201	0.253
	24	P29322_F1_d2	P29322	0.080	-22.993	0.252
	25	2q7m_A,B	P20292, P20292	0.052	-26.511	0.251
	1	5nka_A	P29317	0.140	-22.971	0.312
	2	3zln_A	Q07817	0.148	-21.430	0.309
	3	2xby_A	P00742	0.142	-21.699	0.305
	4	3kul_B	P29322	0.119	-23.931	0.298
	5	4trz_B	P56817	0.128	-20.976	0.286
	6	3ac8_A	P06239	0.104	-23.744	0.282
	7	4cwt_A	P07900	0.085	-25.792	0.278
	8	8evb_C	Q16281	0.099	-23.676	0.277
	9	7rhi_D	P29973	0.106	-22.535	0.275
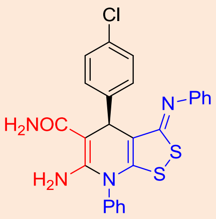 **(*R*)-3b**	10	P42681_F1_d1	P42681	0.092	-23.921	0.271
	11	Q15131_F1_d1	Q15131	0.096	-23.315	0.271
	12	3fsk_A	Q16539	0.090	-23.462	0.266
	13	2py3_A	P21802	0.075	-25.396	0.265
	14	7yaw_C	Q9NYL2	0.084	-23.539	0.260
	15	4twp_A	P00519	0.104	-20.786	0.260
	16	2xir_A	P35968	0.108	-20.154	0.259
	17	3fxx_A	P29320	0.098	-21.308	0.258
	18	5ghv_A	P43405	0.089	-22.479	0.257
	19	5uu1_A	Q86Y07	0.066	-25.505	0.257
	20	4mnf_A	P15056	0.099	-21.072	0.257
	21	7ull_A	P14625	0.070	-24.721	0.256
	22	6e2n_A,B	Q99683, Q99683	0.092	-21.812	0.256
	23	7sqa_B,C	P37231, Q9Y618	0.048	-27.244	0.252
	24	P36894_F1_d2	P36894	0.070	-24.195	0.252
	25	Q13882_F1_d1	Q13882	0.076	-23.352	0.251
	1	2xby_A	P00742	0.142	-22.485	0.311
	2	5nka_A	P29317	0.140	-21.311	0.300
	3	3zln_A	Q07817	0.148	-19.808	0.297
	4	P42681_F1_d1	P42681	0.092	-24.357	0.274
	5	4trz_B	P56817	0.128	-19.185	0.272
	6	3kul_B	P29322	0.119	-20.477	0.272
	7	8evb_C	Q16281	0.099	-22.969	0.271
	8	2jif_A,B,D	P45954, P45954, P45954	0.048	-29.434	0.269
	9	1egd_A,B,C	P11310, P11310, P11310	0.051	-28.654	0.266
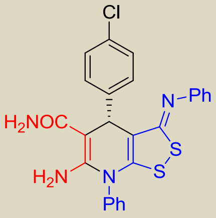 **(*S*)-3b**	10	7rhi_D	P29973	0.106	-21.341	0.266
	11	Q15131_F1_d1	Q15131	0.096	-22.511	0.265
	12	Q13882_F1_d1	Q13882	0.076	-25.111	0.264
	13	3ac8_A	P06239	0.104	-21.315	0.264
	14	Q15569_F1_d1	Q15569	0.067	-26.180	0.264
	15	3zmm_B	O60674	0.099	-21.819	0.262
	16	7yaw_C	Q9NYL2	0.084	-23.715	0.262
	17	3fxx_A	P29320	0.098	-21.838	0.262
	18	P30291_F1_d1	P30291	0.090	-22.648	0.260
	19	4cwt_A	P07900	0.085	-23.118	0.258
	20	8eyk_F	P49327	0.075	-24.427	0.258
	21	4mnf_A	P15056	0.099	-20.983	0.256

	22	2dq7_X	P06241	0.076	-23.981	0.256
	23	5uch_B	P08238	0.080	-23.302	0.255
	24	4wua_A	Q96SB4	0.070	-24.568	0.254
	25	5k3m_A	P51449	0.058	-26.194	0.254
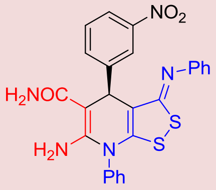 **(*R*)-3c**	1	6uvc_A	Q07817	0.184	-20.078	0.335
	2	4r0i_A	Q9Y5Y6	0.156	-20.781	0.312
	3	2q7m_B,C	P20292, P20292	0.059	-29.401	0.279
	4	3k9x_B	P00742	0.120	-20.368	0.273
	5	1pkd_A	P24941	0.089	-23.976	0.269
	6	4gu6_A	Q05397	0.084	-24.629	0.268
	7	Q7Z3E1_F1_d2	Q7Z3E1	0.068	-26.708	0.268
	8	4yt6_H	P08709	0.122	-19.155	0.265
	9	7wld_S,T,U	Q969N2, Q96S52, Q9H490	0.056	-27.683	0.264
	10	2srt_A	P08254	0.070	-25.062	0.258
	11	4iph_A	P27694	0.131	-16.618	0.256
	12	3khv_A	P00749	0.098	-21.003	0.255
	13	3ac8_A	P06239	0.073	-23.956	0.253
	14	6r6y_A	O43570	0.083	-22.614	0.252
	15	1trn_B	P07477	0.104	-19.671	0.251
	16	5mkf_C,D	Q13563, Q13563	0.057	-25.634	0.249
	17	1wbp_A	Q96SB4	0.066	-24.279	0.248
	18	8gv8_A	P04920	0.059	-25.023	0.247
	19	7yaw_A	Q9NYL2	0.058	-25.019	0.246
	20	1rd4_C,D	P20701, P20701	0.058	-24.976	0.245
	21	7n9g_A,B	P00519, P00519	0.057	-24.919	0.244
	22	4lxd_A	P10415	0.098	-19.465	0.244
	23	4mnf_B	P15056	0.065	-23.720	0.243
	24	Q5JU69_F1_d1	Q5JU69	0.060	-24.331	0.242
	25	8gfa_D	Q8NER1	0.065	-23.568	0.242
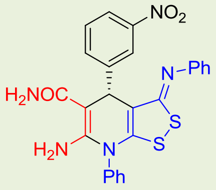 **(S)-3c**	1	6vwc_B	Q07817	0.170	-21.037	0.328
	2	2p93_A	P00742	0.133	-22.404	0.301
	3	4o97_A	Q9Y5Y6	0.137	-20.932	0.294
	4	1pkd_A	P24941	0.103	-24.200	0.285
	5	4gu6_A	Q05397	0.091	-24.445	0.274
	6	4yt6_H	P08709	0.120	-20.337	0.272
	7	3khv_A	P00749	0.108	-21.122	0.266
	8	4tqb_A	P06730	0.096	-22.604	0.266
	9	O15197_F1_d3	O15197	0.051	-28.647	0.266
	10	4fsl_E	P56817	0.098	-22.015	0.263
	11	2pqf_C	Q9H0J9	0.050	-27.859	0.259
	12	6r6y_A	O43570	0.102	-20.992	0.259
	13	1trn_B	P07477	0.104	-20.447	0.257
	14	8hmy_E	Q92989	0.049	-27.748	0.257
	15	8evb_C	Q16281	0.082	-23.218	0.256
	16	P29322_F1_d2	P29322	0.080	-23.175	0.254
	17	Q9UKR0_F1_d1	Q9UKR0	0.078	-23.281	0.252
	18	Q5JU69_F1_d1	Q5JU69	0.078	-23.168	0.251
	19	7wqw_B	P98073	0.072	-23.876	0.251
	20	P42681_F1_d1	P42681	0.066	-24.468	0.250
	21	4lxd_A	P10415	0.090	-21.268	0.249

	22	6qff_A	Q92876	0.070	-23.846	0.249
	23	7oam_B	Q12866	0.061	-24.987	0.248
	24	4i6b_A	Q9NYY3	0.055	-25.779	0.248
	25	8eob_A	P08238	0.063	-24.691	0.248
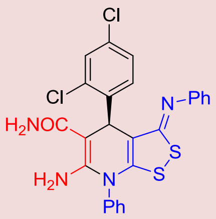 **(*R*)-3d**	1	5nka_A	P29317	0.123	-24.021	0.303
	2	3kul_B	P29322	0.105	-24.202	0.286
	3	6uvc_A	Q07817	0.126	-20.699	0.281
	4	1xkb_C	P00742	0.110	-21.491	0.271
	5	2py3_A	P21802	0.065	-26.616	0.264
	6	5lay_E	Q00987	0.103	-21.325	0.263
	7	7rhh_D	P29973	0.091	-22.872	0.262
	8	3bys_A	P06239	0.091	-22.813	0.262
	9	5tq4_A	O60674	0.081	-24.094	0.261
	10	8eu3_B	Q16281	0.088	-22.619	0.258
	11	2dq7_X	P06241	0.071	-24.680	0.256
	12	P42681_F1_d1	P42681	0.074	-24.271	0.256
	13	P29320_F1_d2	P29320	0.084	-22.739	0.255
	14	4fsl_E	P56817	0.095	-21.070	0.253
	15	2xir_A	P35968	0.088	-22.013	0.253
	16	8eob_A	P08238	0.058	-25.707	0.251
	17	4nfn_A	Q5TCY1	0.071	-23.964	0.250
	18	2wma_C	P24941	0.067	-24.421	0.250
	19	4iph_A	P27694	0.118	-17.520	0.250
	20	6e2n_A,B	Q99683, Q99683	0.072	-23.624	0.249
	21	7yaw_C	Q9NYL2	0.071	-23.697	0.249
	22	7k0v_C	P15056	0.077	-22.763	0.248
	23	Q9UF33_F1_d1	Q9UF33	0.067	-24.087	0.248
	24	4mxo_B	P12931	0.063	-24.520	0.247
	25	Q15131_F1_d1	Q15131	0.068	-23.840	0.247
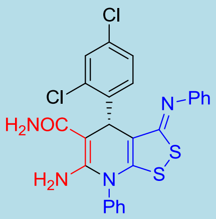 **(*S*)-3d**	1	5lay_E	Q00987	0.118	-23.576	0.295
	2	6uvc_A	Q07817	0.135	-20.769	0.291
	3	4iph_A	P27694	0.144	-16.883	0.271
	4	7k0v_C	P15056	0.065	-26.637	0.265
	5	3cen_A	P00742	0.091	-22.105	0.257
	6	7dsx_A,B	P19634, P19634	0.061	-26.129	0.257
	7	5nka_A	P29317	0.083	-22.798	0.254
	8	P11511_F1_d1	P11511	0.066	-24.842	0.253
	9	Q99988_F1_d1	Q99988	0.060	-25.462	0.251
	10	7rhh_D	P29973	0.069	-24.080	0.250
	11	4fsl_E	P56817	0.084	-21.800	0.248
	12	1yze_C	Q93009	0.068	-23.881	0.247
	13	1gzq_A	P29016	0.066	-24.006	0.246
	14	8eu3_B	Q16281	0.075	-22.673	0.245
	15	Q8IZD2_F1_d1	Q8IZD2	0.060	-24.585	0.244
	16	7d4p_C,D	Q9UL62, Q9UL62	0.068	-23.371	0.244
	17	4wa9_A	P00519	0.067	-23.486	0.243
	18	3zns_A	Q8WUI4	0.075	-22.341	0.243
	19	5ek0_B,C	Q15858, Q15858	0.069	-22.991	0.242
	20	2fxf_A	P21673	0.065	-23.491	0.241
	21	5z62_A,B,C,H,N	O00483, P00395,<br>P00403, P00414,	0.062	-23.906	0.241

			P14854			
	22	3vo3_A	P35968	0.079	-21.537	0.240
	23	7tj9_A	Q01118	0.069	-22.788	0.240
	24	6m5e_B	P02768	0.061	-23.901	0.240
	25	4guk_B	P62166	0.075	-21.964	0.240
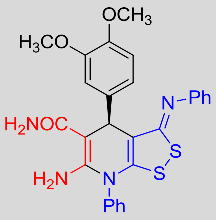 **(*R*)-3e**	1	2xby_A	P00742	0.238	-22.361	0.406
	2	3zlr_B	Q07817	0.221	-24.133	0.402
	3	4jzf_H	P08709	0.158	-21.061	0.316
	4	4r0i_A	Q9Y5Y6	0.161	-20.344	0.314
	5	3khv_A	P00749	0.143	-21.602	0.305
	6	1trn_A	P07477	0.137	-21.295	0.296
	7	1bhx_B,F	P00734, P00734	0.113	-24.375	0.295
	8	3ac8_A	P06239	0.109	-23.601	0.286
	9	5to3_B	P07204	0.122	-21.287	0.282
	10	3lc3_C	P00740	0.123	-21.083	0.281
	11	3vnh_A	Q14145	0.151	-16.614	0.275
	12	1b3d_B	P08254	0.099	-23.438	0.275
	13	8bio_A	P29317	0.095	-23.881	0.274
	14	3gov_B	P48740	0.123	-19.882	0.273
	15	Q9NZQ0_F1_d1	Q9NZQ0	0.117	-20.536	0.271
	16	Q9Y227_F1_d1	Q9Y227	0.109	-21.664	0.271
	17	P29322_F1_d2	P29322	0.090	-24.171	0.271
	18	7rhg_A	P29973	0.091	-23.934	0.270
	19	8evb_C	Q16281	0.096	-23.240	0.270
	20	6o1s_E	P03952	0.123	-19.545	0.270
	21	Q6ZWK6_F1_d2	Q6ZWK6	0.114	-20.807	0.270
	22	6r6y_A	O43570	0.094	-23.276	0.268
	23	P42681_F1_d1	P42681	0.078	-25.370	0.268
	24	2xk7_A	P51955	0.095	-22.682	0.265
	25	Q9BQR3_F1_d1	Q9BQR3	0.107	-21.020	0.265
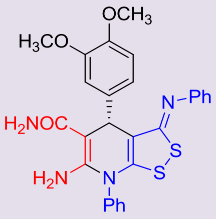 **(*S*)-3e**	1	2xby_A	P00742	0.238	-22.022	0.404
	2	3zlr_B	Q07817	0.221	-21.606	0.383
	3	3khv_A	P00749	0.143	-24.090	0.324
	4	4jzf_H	P08709	0.158	-21.160	0.317
	5	4r0i_A	Q9Y5Y6	0.161	-20.511	0.315
	6	3qtv_H	P00734	0.135	-22.203	0.302
	7	1trn_A	P07477	0.137	-21.415	0.297
	8	3lc3_C	P00740	0.123	-22.795	0.294
	9	1zsk_A	P03951	0.105	-23.921	0.285
	10	3vnh_A	Q14145	0.151	-17.788	0.284
	11	3gov_B	P48740	0.123	-21.110	0.282
	12	8evb_C	Q16281	0.096	-24.168	0.277
	13	Q9BQR3_F1_d1	Q9BQR3	0.107	-22.571	0.277
	14	7yaw_C	Q9NYL2	0.088	-25.157	0.277
	15	5to3_B	P07204	0.122	-20.561	0.276
	16	Q6ZMR5_F1_d2	Q6ZMR5	0.110	-21.928	0.274
	17	6o1s_E	P03952	0.123	-20.045	0.274
	18	1t31_A	P23946	0.105	-22.335	0.273
	19	Q9Y227_F1_d1	Q9Y227	0.109	-21.803	0.272
	20	Q6ZWK6_F1_d2	Q6ZWK6	0.114	-20.579	0.268
	21	Q15569_F1_d1	Q15569	0.074	-25.779	0.267

	22	4j5q_A	Q9Y530	0.074	-25.689	0.267
	23	1rd4_A	P20701	0.095	-22.768	0.266
	24	1b3d_B	P08254	0.099	-22.116	0.265
	25	P29322_F1_d2	P29322	0.090	-23.195	0.264
	1	3zln_A	Q07817	0.195	-21.226	0.354
	2	7ahu_E	P00742	0.168	-18.088	0.304
	3	3ac8_A	P06239	0.114	-23.744	0.293
	4	2g2h_A	P00519	0.125	-21.258	0.284
	5	5za9_U	P00749	0.119	-21.856	0.283
	6	4gu6_A	Q05397	0.104	-23.786	0.282
	7	4mnf_B	P15056	0.106	-23.415	0.281
	8	P29322_F1_d2	P29322	0.102	-23.666	0.279
	9	8eob_A	P08238	0.080	-25.810	0.273
	10	P42681_F1_d1	P42681	0.090	-24.442	0.273
	11	5nk5_A	P29317	0.106	-22.317	0.273
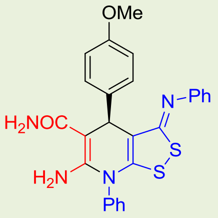	12	4yt6_H	P08709	0.131	-18.778	0.272
	13	1trn_A	P07477	0.116	-20.504	0.270
	14	Q15131_F1_d1	Q15131	0.092	-23.440	0.268
	15	4fst_A	O14757	0.095	-22.766	0.266
**(*R*)-3f**	16	1hxe_H	P00734	0.092	-23.127	0.265
	17	8evc_C	Q16281	0.094	-22.744	0.264
	18	7z5x_B	P08922	0.097	-22.112	0.263
	19	3znr_A	Q8WUI4	0.087	-23.532	0.263
	20	Q15569_F1_d1	Q15569	0.080	-24.303	0.262
	21	4r0i_A	Q9Y5Y6	0.104	-21.063	0.262
	22	1wbv_A	Q16539	0.100	-21.545	0.261
	23	2pzp_A	P21802	0.067	-25.837	0.261
	24	4nh9_A	P14625	0.091	-22.671	0.261
	25	1xbb_A	P43405	0.087	-22.836	0.258
	1	3zln_A	Q07817	0.195	-20.578	0.349
	2	7ahu_E	P00742	0.168	-19.986	0.318
	3	4gu6_A	Q05397	0.104	-25.151	0.292
	4	4yt6_H	P08709	0.131	-21.267	0.291
	5	5za9_U	P00749	0.119	-21.811	0.283
	6	P29322_F1_d2	P29322	0.102	-23.814	0.280
	7	P42681_F1_d1	P42681	0.090	-25.047	0.278
	8	8eob_A	P08238	0.080	-26.222	0.277
	9	3ac8_A	P06239	0.114	-21.467	0.275
	10	2g2h_A	P00519	0.125	-19.971	0.275
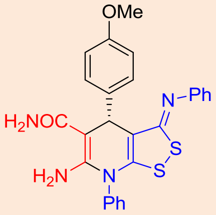	11	Q15569_F1_d1	Q15569	0.080	-25.907	0.274
	12	8evc_C	Q16281	0.094	-23.821	0.272
	13	3gov_B	P48740	0.104	-22.394	0.272
**(*S*)-3f**	14	3znr_A	Q8WUI4	0.087	-24.622	0.271
	15	7yaw_C	Q9NYL2	0.088	-24.029	0.268
	16	6o1s_E	P03952	0.110	-20.993	0.267
	17	Q13882_F1_d1	Q13882	0.074	-25.621	0.266
	18	5nk5_A	P29317	0.106	-21.262	0.265
	19	1wbv_A	Q16539	0.100	-22.032	0.265
	20	6fad_D	Q96SB4	0.078	-24.796	0.264
	21	6sgk_A	P51955	0.096	-22.459	0.264
	22	4nfn_A	Q5TCY1	0.090	-23.156	0.264

	23	4g2f_A	P29320	0.099	-21.959	0.263
	24	1trn_A	P07477	0.116	-19.653	0.263
	25	4lxd_A	P10415	0.096	-22.233	0.263
